# Exploring potential roles of long non-coding RNAs in cancer immunotherapy: a comprehensive review

**DOI:** 10.3389/fimmu.2024.1446937

**Published:** 2024-08-27

**Authors:** Asghar Arshi, Esmaeil Mahmoudi, Farzaneh Raeisi, Masoud Dehghan Tezerjani, Elham Bahramian, Yeasin Ahmed, Chun Peng

**Affiliations:** ^1^ Department of Biology, York University, Toronto, ON, Canada; ^2^ Young Researchers and Elite Club, Islamic Azad University, Shahrekord, Iran; ^3^ Department of bioinformatics, School of Advanced Medical Technologies, Isfahan University of Medical Sciences, Isfahan, Iran; ^4^ Department of Biological Sciences, University of Arkansas, Fayetteville, AR, United States

**Keywords:** cancer immunotherapy, lncRNA, therapeutic target, cancer biomarkers, immune response

## Abstract

Cancer treatment has long been fraught with challenges, including drug resistance, metastasis, and recurrence, making it one of the most difficult diseases to treat effectively. Traditional therapeutic approaches often fall short due to their inability to target cancer stem cells and the complex genetic and epigenetic landscape of tumors. In recent years, cancer immunotherapy has revolutionized the field, offering new hope and viable alternatives to conventional treatments. A particularly promising area of research focuses on non-coding RNAs (ncRNAs), especially long non-coding RNAs (lncRNAs), and their role in cancer resistance and the modulation of signaling pathways. To address these challenges, we performed a comprehensive review of recent studies on lncRNAs and their impact on cancer immunotherapy. Our review highlights the crucial roles that lncRNAs play in affecting both innate and adaptive immunity, thereby influencing the outcomes of cancer treatments. Key observations from our review indicate that lncRNAs can modify the tumor immune microenvironment, enhance immune cell infiltration, and regulate cytokine production, all of which contribute to tumor growth and resistance to therapies. These insights suggest that lncRNAs could serve as potential targets for precision medicine, opening up new avenues for developing more effective cancer immunotherapies. By compiling recent research on lncRNAs across various cancers, this review aims to shed light on their mechanisms within the tumor immune microenvironment.

## 1 Introduction

Cancer is a complex genetic, epigenetic, and environmental disease with an expansive range of tissue, tumor, and cellular levels (1). Additionally, cancer is the second-highest cause of mortality in terms of disability-adjusted life years (DALYs), and it carries the highest clinical, social, and economic burden among human diseases (2). The rate at which cancer progresses depends on a person’s biological, immunological, genetic, and environmental background. Furthermore, the complexity of cancer increases as various gene classes are discovered, particularly tumor-suppressor genes and molecular pathways (3). Cancer cells disregard the rules of cell division by disrupting the order of the body’s cells and resuming their new path. Cancer can stimulate the formation of blood vessels to ensure a continuous supply of nutrients, attack normal tissues, and disrupt their natural processes. Eventually, the cancer cells undergo a process that renders the immune system inoperable (4).

There are many risk factors for developing cancer that fall into two main categories, namely, intrinsic risk factors and non-intrinsic risk factors. Intrinsic risk factors are defined as random errors caused by DNA replication. Then, intrinsic risk factors are classified into two major subgroups: endogenous (biological aging, genetic susceptibility, DNA repair machinery, hormones, growth factors, inflammation, etc.) and exogenous (radiations, chemical carcinogens, tumor-causing viruses, smoking, lack of exercise, nutrient imbalance, etc.) risk factors. Overall, all these risk factors are triggers for cancer (5).

Initially, cancer was considered a genetic disease due to genetic mutations associated with loss of gene function or overexpression, with the assumption that these mutations are the main factor in pathogenesis and disease progression. However, cancer follows accumulative genetic mutations associated with epigenetic changes as well as environmental factors. Many cancer-like pathological conditions can develop and progress with incorrect epigenetic changes, and evidence is emerging that highlights the critical role of epigenetics in carcinogenesis. In this way, epigenetics manages the transcription and posttranscriptional regulation of many genes. These genes control many cellular processes and functions, such as growth, metabolism, immune responses, invasion, and proliferation (6). DNA methylation, histone modification, and regulation by non-coding RNAs (ncRNAs) are the principal epigenetic mechanisms. Furthermore, ncRNA, a functional RNA molecule that transcribes but does not translate into proteins, significantly influences the expression of epigenetic genes.

Long non-coding RNAs (lncRNAs) are notable ncRNA molecules that are major regulators of the epigenetic status of the human genome (6–8). In addition to their involvement in natural physiology, diseases such as cancer are associated with the function and expression of lncRNAs. Studies have demonstrated that numerous disorders in the expression of cancer-specific lncRNAs, such as the misregulation of gene expression that contributes to tumorigenesis, exist. Consequently, the potential role of lncRNAs in regulating multiple biological functions makes them excellent candidates for cancer treatment. To wrap up, controlling the function of lncRNAs in the treatment pathway can effectively contribute to the development of cancer treatments, including cancer immunotherapy (9–13). Recent studies reveal a systematic alteration of RNA levels in cancer. To make these changes happen, the amount of mutations in the genes that code for RNA processing factors, the amount of RNA processing factors, and the type of ncRNAs (like lncRNAs, miRNAs, snRNAs, and circRNAs) all play a role. Indeed, identifying and investigating the mechanisms that are processed by these different RNA subspecies offers opportunities for cancer treatment intervention (9, 10, 13–17).

Different treatments have targeted cancers over the last few decades. Common cancer treatments include surgery, chemotherapy, radiation therapy, hormone therapy, immunotherapy, stem cell therapy, and targeted therapy, each of which varies according to the patient’s clinical parameters. Also, in this study, we discuss cancer immunotherapy in detail. High-potential cancer immunotherapy has caught the attention of the world’s advanced therapies. Then, it aims to create an antitumor effect through the immune system’s response to cancer cells, both preventively and therapeutically (12, 18, 19). Importantly, lncRNAs play a key role in regulating cancer immunity (for example, in the cancer immunity cycle) and immune cells’ transcriptional profiles. The immune system effectively controls the growth of cancer cells through the cancer immunity cycle, which includes seven steps: release of tumor antigens, antigen presentation, immune cell priming and T-cell activation, immune cell migration, immune cell infiltration, recognition, and attack by T cells (10, 11, 13, 17, 19). Then, recent emerging evidence suggests lncRNAs are involved in tumor-stroma overlap and tumor immune microenvironment (TIME) reprogramming. The TIME plays critical roles in cancer development, progression, and control. The TIME has distinct groups of myeloid cells and lymphocytes that influence cancer immune escape, immunotherapy response, and patient survival. On the other hand, understanding the molecular mechanisms and functional roles of lncRNAs in the TIME is helpful for immunotherapy, especially for improving immunotherapy for cancer (11, 12, 18, 20). In this article, we review the advances and challenges in the clinical development of cancer immunotherapy, with a focus on lncRNAs.

In this review, we explore the critical role of lncRNAs in cancer immunotherapy, with a particular focus on their influence on the tumor immune microenvironment and their potential as therapeutic targets. The manuscript is structured to provide a detailed and comprehensive review of the topic. It begins with an overview of cancer immunotherapy, covering various approaches. Following this, the paper delves into the biogenesis and function of lncRNAs, particularly their roles in cancer. It then examines the interaction between lncRNAs and the tumor immune microenvironment, detailing their effects on various immune cells. The discussion then shifts to the potential of lncRNAs as targets for cancer immunotherapy, followed by a discussion of the key findings and their implications. The paper concludes by summarizing the main points and suggesting directions for future research. The primary objective of this paper is to compile and analyze recent research on lncRNAs in various cancers, focusing on their roles within the tumor immune microenvironment, to encourage further exploration and development of precise and effective cancer immunotherapies.

## 2 Cancer immunotherapy

### 2.1 Immune checkpoint inhibitors

Immune checkpoints regulate the immune system by initiating a productive immune response against metastatic cancer cells (**Table 1**). Immune checkpoint inhibitors (ICIs) are possible drugs that stop inhibitory checkpoint molecules from working. This makes the immune system stronger so it can attack a wider range of cells, especially cancer cells that have been hiding from the immune system in the past. One area of intense research is on monoclonal antibodies that target cytotoxic t-lymphocyte-associated protein 4 (CTLA-4), programmed cell death protein 1 (PD-1), and programmed cell death ligand 1 (PD-L1). These antibodies can make T cells more effective at killing cancer cells, which in turn stops the growth of tumors. CTLA-4 is mostly found inside cells and is expressed on CD4+ and CD8+ T cells. It is related to the immunoglobulin-related receptor B7 family. When interacting with CD80 and CD86, CTLA-4 mainly deregulated the immune response of T cells to antigen-presenting cells. The cytoplasm abundantly contains CD28, a homologous receptor that competes with CTLA-4 for affinity binding. CD28 usually enhances the T-cell immune response. The TRIM/LAX/Rab8 pathway is used by CTLA-4 to get out of cells and move to the outer member. This helps T cells mount an immune response (21). PD-1 is another important protein that keeps the T-cell immune response in check upon binding with PD-L1 and PD-L2. PD-1 is a B7 homolog protein encoded by the CD274 gene in humans and expressed in both hematopoietic and non-hematopoietic cells. CD4+ cells express PD-1, a type 1 transmembrane receptor. When PD-1 ligation happened, protein tyrosine phosphates bound to PD-1 phosphorylation motifs. This caused the T-cell signaling molecule of the T-cell receptor (TCR) complex to lose its phosphorylation. This results in inhibition of IL-2 release, one of the major T-cell growth factors. There are several potential immune checkpoints that could serve as potential candidates for immune checkpoint inhibitors (ICIs). NcRNA-RB1 is an lncRNA expressed by the RB1 promotor. It has the potential to impair tumor-inhibiting mechanisms by inhibiting CALR expression in cytotoxic CD8+ T cells. LncRNA ROR is a stress-responsive lncRNA that turns on the TGF-β pathway. This makes more CD-133+ cells proliferate, which makes chemotherapy less effective (22).

**Table 1:** Overview of immune checkpoint inhibitors and their clinical applications.

Drug name	Trade name	Checkpoint target	Approved cancer types	Mechanism of action	References
Nivolumab	Opdivo	PD-1	Melanoma, non-small cell lung cancer, kidney cancer, bladder cancer, head and neck cancer, Hodgkin lymphoma, microsatellite instability-high (MSI-H) or mismatch repair deficient (dMMR) solid tumors	Blocks PD-1 on T cells, allowing them to recognize and attack cancer cells	(23)
Pembrolizumab	Keytruda	PD-1	Melanoma, non-small cell lung cancer, bladder cancer, head and neck cancer, microsatellite instability-high (MSI-H) or mismatch repair deficient (dMMR) solid tumors, Merkel cell carcinoma, triple-negative breast cancer, cervical cancer	Blocks PD-1 on T cells, allowing them to recognize and attack cancer cells	(24)
Ipilimumab	Yervoy	CTLA-4	Melanoma, kidney cancer	Blocks CTLA-4, which helps activate T cells, leading to a broader immune response	(25)
Atezolizumab	Tecentriq	PD-L1	Non-small cell lung cancer, bladder cancer, urothelial carcinoma, melanoma, triple-negative breast cancer, hepatocellular carcinoma	Blocks PD-L1 on cancer cells, preventing them from evading T-cell attack	(26)
Durvalumab	Imfinzi	PD-L1	Non-small cell lung cancer, bladder cancer, head and neck cancer, urothelial carcinoma	Blocks PD-L1 on cancer cells, preventing them from evading T-cell attack	(27)
Avelumab	Bavencio	PD-L1	Merkel cell carcinoma, urothelial carcinoma, non-small cell lung cancer	Blocks PD-L1 on cancer cells, preventing them from evading T-cell attack	(28)
Cemiplimab	Libtayo	PD-1	Cutaneous squamous cell carcinoma	Blocks PD-1 on T-cells, allowing them to recognize and attack cancer cells	(29)
Tislelizumab	Tiguli	PD-1	Hepatocellular carcinoma	Blocks PD-1 on T cells, allowing them to recognize and attack cancer cells	(30)
Camrelizumab	Hemlibra	PD-1	Gastric cancer, esophageal adenocarcinoma	Blocks PD-1 on T cells, allowing them to recognize and attack cancer cells	(31)
Dostarlimab	Jevtana	PD-1	Endometrial cancer	Blocks PD-1 on T cells, allowing them to recognize and attack cancer cells	(32)
Tigelizumab	Tyvyt	TIGIT	Merkel cell carcinoma	Blocks TIGIT on T cells, preventing suppression and enhancing antitumor response	(33)
Fasenra (dupilumab)	Fasenra	IL-4Rα	Severe eosinophilic asthma, chronic rhinosinusitis with nasal polyps	Blocks IL-4 signaling, reducing inflammation and allergy-related symptoms	(34)
Libtayo (cemiplimab-rwlc)	Libtayo	PD-1	Basal cell carcinoma	Blocks PD-1 on T-cells, allowing them to recognize and attack cancer cells	(35)
Tecentriq (atezolizumab pegol)	Tecentriq	PD-L1	Small cell lung cancer	Blocks PD-L1 on cancer cells, preventing them from evading T-cell attack	(36)
Keytruda (pembrolizumab pegol)	Keytruda	PD-1	Microsatellite instability-high (MSI-H) or mismatch repair deficient (dMMR) colorectal cancer	Blocks PD-1 on T-cells, allowing them to recognize and attack cancer cells	(37)

### 2.2 Adoptive cell therapies

Adoptive cell therapy (ACT) is a revolution in immunotherapy, improving the natural T-cell response to tumors (**Supplementary Table 1**). The T-cell response against tumor-specific antigens is too weak. We surgically remove cells from ACT melanoma and culture them *in vitro* with IL-2 to herald T cells infiltrating the tumor. This process allows the resident T cells to get the resources against melanoma-specific antigens and differentiate into effector T cells in the culture. We harvest this pure culture of effector T cells and infuse them into the patient’s circulation. The effector T cells actively bind to residual melanoma and selectively diminish their proliferation. ACT immunotherapy employs multiple approaches, including enhancing the T-cell receptor’s affinity for a specific combination of HLA class-1 and genetically allocating peptides to the chimeric antigen receptor (CAR), engineered to exhibit high binding specificity to the defined ligands of tumor cells. In B-cell tumors, CAR-T cells, which express CAR, exhibit high specificity for the CD19 antigen. Finally, we took dendritic cells (monocytes) and cultured them with the recombinant fusion protein sipuleucel-T to treat prostate cancer that has spread (38). Dendritic cells (DC) are the most common antigen-presenting cells. There is a specific marker named lnc-DC. The lnc-DC solely determines the differentiation of DCs. Inhibition of lnc-DC affects the differentiation, proliferation, and growth of the DC and, most importantly, inhibits the DCs from activating T cells. The transcription factor STAT3 regulates lnc-DC function. Lnc-DC plays a crucial role in STAT 3’s movement into the nucleus and the maintenance of transcription activity, with STAT3 setting an example in antigen presentation (39). The balance between T-regulatory cells and T-effector cells is really important for the immunological response; lncRNAs contribute to the differentiation and activation of lymphocytes. The “CECR-7-miR429-CTL4” network system, which is made up of several regulatory genes working together, controls how lymphocytes differentiate. LncRNA CECR7 targets miR-429 to regulate CTLA-4 expression. SGK-1/JunB signaling pathway helps TH2 and Th17 cells differentiate. Other regulatory genes, like linc-serum and glucocorticoid-inducible kinase-1 (Lnc-SGK-1), do the same thing. Linc-MAF is associated with Th1 and Th2 cell differentiation and proliferation by recruiting the repressor protein enhancer of Zesta homolog (EZH2) and lysine-specific demethylase (LSD1), which helps T-cell differentiation toward the Th2 phenotype. It is evident that lncRNAs play a crucial role not only in antigen presentation but also in immune cell differentiation. These contributions range from regulating the STAT3 pathway to inducing over-maturing of decidual DCs, which in turn influences the differentiation of CD8+ cells into Th1 cells, leading to increased inflammation (40).

### 2.3 Monoclonal antibodies

**Figure 1:**
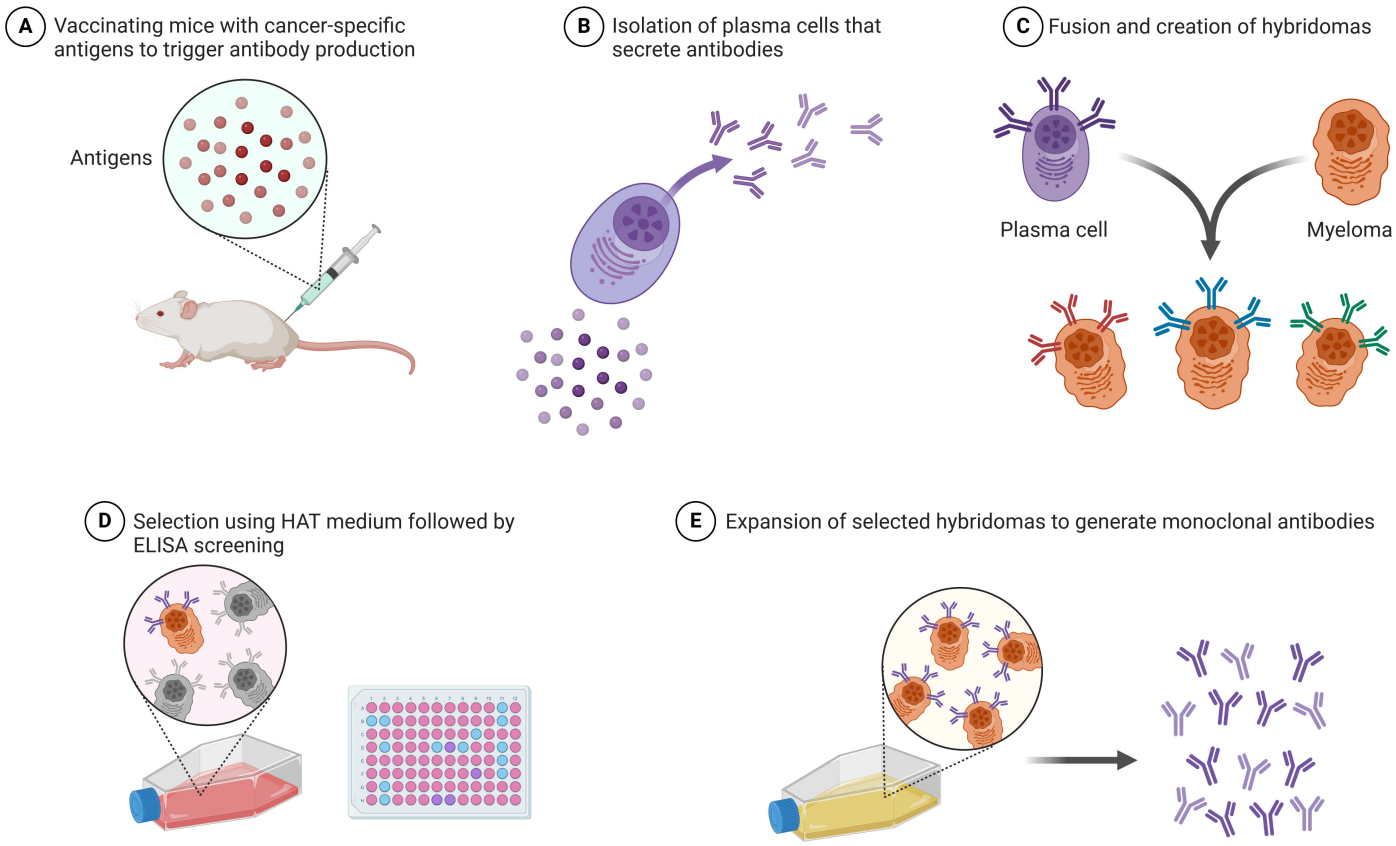
Production of monoclonal antibodies: step-by-step process involved in the production of monoclonal antibodies (this image was created with BioRender).

The most significant advancement in cancer immunotherapy is the development of monoclonal antibodies (**Table 2**). Monoclonal antibodies aid in the diagnosis of specific tumors as well as precise cancer treatment. **Figure 1** illustrates the production process of monoclonal antibodies, from vaccinating mice with cancer-specific antigens to expanding selected hybridomas for antibody generation. CD30 and CD15, expressed by tumor cells, are APC-associated markers. These markers are capable of distinguishing Hodgkin’s lymphoma from other B-cell lymphomas. Conversely, T-cell lymphomas typically display CD30 on their cell surface, serving as a target for therapeutic monoclonal antibodies. The management of Hodgkin’s lymphoma and ALCL involves the use of an anti-CD-30 monoclonal antibody (brentuximab), immunotoxin auristatin (vedotin), and a cleavable linker called cathepsin. On the cell surface, the CD30 conjugate binds to the brentuximab–vedotin conjugate. Cellular cathepsin cleaves off this conjugate upon reaching the cytoplasm. Brentuximab enters the nucleus and inhibits the further division and proliferation of lymphoma cells (41). Human monoclonal antibodies can be administered as “naked antibodies” or as conjugates with cytotoxins or radioisotopes. Naked antibodies reduce tumor growth by adopting a diverse approach, starting with inhibiting the signaling receptor and promoting the opsonization of tumor cells, phagocytosis by NK cells, and most importantly, preventing angiogenesis with antibodies specific to the cell surface of vascular endothelial growth factor (VEGF) (42). Monoclonal antibodies that target inhibitory T-cell response regulators are effective cancer therapies. Advanced melanoma effectively uses ipilimumab, a fully humanized monoclonal anti-CTLA-4 antibody. For this monoclonal antibody, the treatment plan was to stop the checkpoint inhibitor CTLA-4 from working. This stops the normal T-cell response. Ipilimumab stops CTLA-4 from binding to B7 and sending out inhibitory signals that set off the T-cell response by binding to CTLA-4. Nivolumab is an effective treatment for renal cell carcinoma, non-small cell lung cancer, and bladder cancer. Nivolumab is a human IgG4 monoclonal antibody that binds to PD-1 and halts PD-1 from engaging with PD-L1, which attenuates T cells’ antitumor response. T cells, members of the B7 family of co-stimulators, express PD-1 and CD279, which exclusively bind to PD-L1. Engaging T-cell PD-1 with PD-L1 exhausts T cells, triggering an epigenetic program of T-cell exhaustion. Nivolumab inhibits the engagement of PD-L1 in the TIME, thereby enhancing the ability of effector T cells to encounter their tumor antigens (43).

**Table 2:** Monoclonal antibodies for cancer therapy: targets, indications, and pharmacological profiles.

Antibody name	Target	Cancer indication	Mechanism of action	Adverse effects	Dosage form	References
Rituximab (Rituxan)	CD20	Non-Hodgkin lymphoma, chronic lymphocytic leukemia	Kills B cells expressing CD20 through complement activation and immune cell recruitment	Infusion reactions, fatigue, nausea	Intravenous infusion	(44)
Trastuzumab (Herceptin)	HER2	HER2-positive breast cancer, gastric cancer	Inhibits HER2 signaling, triggers immune cell attack on cancer cells	Infusion reactions, diarrhea, rash	Intravenous infusion	(45)
Bevacizumab (Avastin)	VEGF	Colorectal cancer, non-small cell lung cancer, glioblastoma	Inhibits blood vessel growth, starving cancer cells	Hypertension, protein in urine, bleeding	Intravenous infusion	(46)
Cetuximab (Erbitux)	EGFR	Colorectal cancer, head and neck cancer	Blocks EGFR signaling, inhibits cancer cell growth	Skin rash, fatigue, diarrhea	Intravenous infusion	(47)
Pembrolizumab (Keytruda)	PD-1	Melanoma, non-small cell lung cancer, head and neck cancer	Blocks PD-1 checkpoint, activates immune system against cancer cells	Fatigue, rash, nausea, infusion reactions	Intravenous infusion	(48)
Ipilimumab (Yervoy)	CTLA-4	Melanoma	Blocks CTLA-4 checkpoint, unleashes immune attack on cancer cells	Diarrhea, skin rash, fatigue	Intravenous infusion	(49)
Daratumumab (Darzalex)	CD38	Multiple myeloma	Kills myeloma cells expressing CD38 through multiple mechanisms	Infusion reactions, fatigue, low blood counts	Intravenous infusion	(50)
Atezolizumab (Tecentriq)	PD-L1	Bladder cancer, non-small cell lung cancer, melanoma	Blocks PD-L1 checkpoint, activates immune system against cancer cells	Fatigue, nausea, rash, infusion reactions	Intravenous infusion	(51)
Dinutuximab (Dinutuximab Tecmab)	GD2	High-risk neuroblastoma	Targets GD2 antigen on neuroblastoma cells, triggers immune attack	Fever, infusion reactions, pain	Intravenous infusion	(52)
Brentuximab vedotin (Adcetris)	CD30	Hodgkin lymphoma, anaplastic large cell lymphoma	Delivers cytotoxic chemotherapy to CD30-positive cells	Peripheral neuropathy, fatigue, nausea	Intravenous infusion	(53)
Nivolumab (Opdivo)	PD-1	Melanoma, non-small cell lung cancer, head and neck cancer, renal cell carcinoma	Blocks PD-1 checkpoint, activates immune system against cancer cells	Fatigue, rash, nausea, infusion reactions	Intravenous infusion	(54)
Durvalumab (Imfinzi)	PD-L1	Bladder cancer, non-small cell lung cancer, head and neck cancer	Blocks PD-L1 checkpoint, activates immune system against cancer cells	Fatigue, nausea, rash, infusion reactions	Intravenous infusion	(55)
Olaratumab (Lartruvo)	PDGFRα	Gastrointestinal stromal tumors	Inhibits PDGFRα signaling, slows tumor growth	Rash, diarrhea, fatigue	Intravenous infusion	(56)
Enhertu (Polatuzumab vedotin)	HER2, CD3	HER2-positive breast cancer, diffuse large B-cell lymphoma	Delivers cytotoxic chemotherapy to HER2 and CD3 expressing cells	Peripheral neuropathy, nausea, fatigue	Intravenous infusion	(57)
Mosunetuzumab (Lumoxiti)	CD20	Follicular lymphoma, marginal zone lymphoma	Kills B cells expressing CD20 through antibody-dependent cellular cytotoxicity	Infusion reactions, fatigue, nausea	Intravenous infusion	(58)

### 2.4 Oncolytic virus therapy

Significant progress has been made in cancer diagnosis and prevention. Using oncolytic virus infection on tumor cells has brought attention to the field of immunotherapy. Human endogenous retroviral (HERV) reactivation, oncolytic adenovirus in oral squamous cell carcinoma (OSCC) management, and oncolytic vaccina virus (OVV) are some of the most popular treatment strategies in cancer immunotherapy. LncRNA expression is correlated with HERV reactivation. HERV comprises a significant part of the human genome (approximately 8%). HERV proviral DNA is transcribed into dsRNA, which is recognized by pattern recognition receptors as a danger signal or translated into proteins to detect tumor-specific antigens (TSAs). HERV reactivation can be done either by drugs or by inducing cellular changes in the tumor cells. Reactivation of HERV comes up with some possible targets in cancer immunotherapy, such as the production of viral proteins on tumor cells recognized by our immune system, immunoglobulin viral defense responses against tumors, and the expression of mRNA transcripts, which can be used as biomarkers for prognosis. Inherent DNA hypomethylation activates, and overexpression of HERV, which is recognized by the PRRs, induces a viral mimicry state that synergistically shows an antitumor effect. A novel combinational therapy uses the same antitumor strategy based on histone deacetylase inhibitors (HDACIs) (which induce overexpression of HERV) and TLR 7/8 agonists (which induce apoptosis in susceptible HERV de-repressed tumor cells) in human ovarian cancer cells. In testicular germ cells, overexpression of HERV is usually associated with seminomas DNA hypomethylation, which in turn increases the IFN production and infiltration of CD8+ cells, which may potentiate a viral-like immune response against tumors (59). Researchers are investigating the Lentivirus Display System for monoclonal antibody screening, as well as engineered herpes simplex virus, adenovirus, Newcastle virus, and vaccinia virus as potential oncolytic viruses. The entire life cycle of the Vaccinia virus takes place in the cytoplasm, and it does not integrate with the host genome. OVV replicates fast and spreads through the blood, exerting not only a primary antitumor effect but also a metastatic cancer effect through a remote effect (60). LncRNAs are also associated with SNHG1 expression in OSCC and head and neck cancer. Oncolytic adenovirus H101 shows an antitumor effect with high SNHG1 expression in OSCC. SNHG1 expression acts as a biomarker for OSCC prognosis. Poor prognosis associated with SNHG1 lncRNA expression leads to colon cancer tumorigenesis, hepatocellular carcinoma, and the proliferation of non-small cell lung cancers. Knocking down SNHG1 suppresses cell proliferation both *in vivo* and *in vitro* (61).

### 2.5 Cancer vaccines

In 1980, researchers developed a cancer vaccination based on tumor cells and lysates to treat colorectal cancer using autologous cells. Using human tumor antigens as vaccines against cancer started with the discovery of melanoma-associated antigen-1 in the early 1990s (62). Since 2010, DC vaccination has proven effective in treating prostate cancer, demonstrating the potential of cancer vaccination (63). Cancer vaccines use tumor antigens to promote tumor immunity. By overcoming tumor immunological repression, the most powerful cancer vaccines will stimulate humoral and cellular immunity simultaneously. The effectiveness of any cancer vaccination in the clinic greatly depends on the antigen that is selected as its target. The optimal antigen should have immunological function, be essential for cell viability, and only be present in cancer cells with no expression in normal cells. The two main kinds of cancer antigens are tumor-associated antigens (TAAs) and tumor-specific antigens (TSAs) (64). **Supplementary Table 2** presents information on cancer vaccines, distinguishing between preventive and therapeutic vaccines. One possibly beneficial vaccination platform is the nucleic acid vaccine because it elicits robust MHC-I-mediated CD8+ T-cell responses. Several antigens can be delivered concurrently using nucleic acid vaccines, promoting both humoral and cellular protection (65). Researchers primarily use DNA vaccines in clinical studies on cervical, prostate, and breast cancer, whereas melanoma, glioblastoma, and prostate cancer more frequently use mRNA vaccines (18). Small-scale trials investigating mRNA-based immunizations for cancer treatment have been underway for more than a decade, with promising outcomes. In many clinical trials, individuals with melanoma, colorectal cancer, pancreatic cancer, and other malignancies are being investigated for mRNA therapy vaccines (66). In addition, lncRNAs play a role in controlling tumor cell immune evasion and altering the TIME. Researchers can identify and select particular antigens that are crucial for the creation of vaccines using information about lncRNAs. LncRNAs control the activity of immune cells, pathways, and genes. Certain lncRNAs are involved in the regulation of immune response-related genes. Vaccines that elicit a potent and targeted immune response can be developed with the use of knowledge about the epigenetic control of immune-related genes (67). A few of the difficulties encountered in this field are caused by the immunosuppressive properties of cancer, heterogeneity within and between cancer types, inadequate immunogenicity of nucleic acid vaccines, weakened T-cell responses, subpar vaccine formulations and adjuvants, inadequate knowledge of tumor biology and its immune-suppressive TIME, and the selection of suitable tumor-specific antigens. Cancer vaccines have bright future possibilities despite these obstacles. Some of the future prospects comprise mixing different immunotherapies or conventional treatments with cancer vaccines, using personalized mRNA-based cancer vaccines to target neoantigens, combining novel medications with antigen-based therapies, and improving the whole tumor vaccine’s ability to stimulate immune responses that provide protection and improve treatment efficacy against cancers (68).

### 2.6 Immune system modulators

A type of immunotherapy known as immune system modulators works to strengthen the body’s defenses against cancer. They stimulate the immune system to fight cancer cells more vigorously or intelligently (69). These modulators include a variety of forms, such as BCG, cytokines, and immunomodulatory medications. Cytokines produced by white blood cells have vital roles in fighting the immune system against cancer and regulating the body’s immunological responses. Interferons and interleukins, two kinds of cytokines, are utilized in the treatment of several types of cancer. INFs boost the immune system’s capacity to fight tumor cells by stimulating certain white blood cells, such as DC and natural killer cells. They also inhibit cancer development by inducing apoptosis. The body produces more white blood cells, such as killer T cells and NK cells, when exposed to ILs. These cells can then build an immune response against cancer cells (70). Research is ongoing to better understand the immune response to cancer cells and develop drugs that can enhance the immune system’s natural abilities. This entails identifying and clarifying novel immune response-regulating molecules, researching the biology of regulatory T cells, and learning more about the particular biochemical markers that enable a patient’s T cells to target cancer cells only (71).

### 2.7 Epigenetics-related cancer immunotherapy

Epigenetic regulation, encompassing DNA methylation, histone modifications, chromatin remodeling, and non-coding RNAs, plays a pivotal role in gene expression and has significant implications for cancer development and treatment. In cancer, epigenetic dysregulation can lead to aberrant gene expression, contributing to tumor initiation, progression, and metastasis. These epigenetic changes critically influence the TIME, impacting both tumor cells and immune cells (72). For example, epigenetic changes in tumor cells can change the expression of tumor-associated antigens, MHC molecules, and immune checkpoint molecules like PD-L1. This can affect how immune cells recognize and kill cancer cells. Additionally, epigenetic mechanisms regulate the function and differentiation of various immune cell types, including T cells, NK cells, and MDSCs (73). Targeting these epigenetic mechanisms has emerged as a promising approach in cancer therapy, with drugs like DNA methyltransferase (DNMT) inhibitors and HDAC inhibitors showing efficacy in treating various malignancies. However, their use as single agents in solid tumors has been limited, prompting exploration of combination strategies, particularly with ICIs (74).

The intersection of epigenetics and cancer immunotherapy has become an area of intense research, as epigenetic modifications can influence the efficacy of immunotherapy by modulating tumor immunogenicity, enhancing T-cell function and persistence, and reprogramming the immunosuppressive TIME. Combining epigenetic drugs with ICIs has shown promising results in preclinical and clinical studies, aiming to overcome resistance to immunotherapy and improve patient outcomes. Emerging strategies include the development of selective HDAC inhibitors, epigenetic editing using CRISPR-Cas9, targeting epigenetic readers like BET proteins, and exploring the synergistic effects of epigenetic drugs with other therapies. Despite the promise, challenges remain, such as improving drug specificity, identifying reliable biomarkers, understanding resistance mechanisms, determining optimal dosing, and assessing long-term effects. Future research should focus on addressing these challenges and further elucidating the complex interplay between epigenetics and cancer immunology, with the goal of integrating epigenetic regulation to enhance the efficacy of existing immunotherapies and develop novel cancer treatment strategies (75, 76).

Despite the promising advances in epigenetics-related cancer immunotherapy, we still need to address several challenges. Improving the specificity of epigenetic drugs to reduce off-target effects and toxicity is a key area of focus. Identifying reliable biomarkers to predict responses to epigenetic therapies and guide patient selection is critical for optimizing treatment outcomes. Understanding and overcoming resistance mechanisms to epigenetic drugs is another important consideration. Determining the most effective treatment regimens, especially in combination therapies, requires further investigation to establish optimal dosing and scheduling. Assessing the potential long-term consequences of epigenetic modulation is also necessary to ensure the safety and efficacy of these treatments. As research in this field progresses, elucidating the complex interplay between epigenetics and cancer immunology may uncover new therapeutic targets and strategies. The integration of epigenetics and cancer immunotherapy represents a promising frontier in cancer treatment, with the potential to enhance the efficacy of existing immunotherapies and develop innovative approaches to combat cancer (77).

## 3 LncRNAs: biogenesis and function

LncRNAs are a class of RNA molecules that do not encode proteins but play critical roles in regulating gene expression at various levels, including chromatin modification, transcription, and posttranscriptional processing. They are typically longer than 200 nucleotides, distinguishing them from small non-coding RNAs like miRNAs and siRNAs. The biogenesis and function of lncRNAs are complex and multifaceted, involving intricate mechanisms that contribute to their diverse roles in cellular processes and, by extension, in the pathogenesis of diseases, including cancer (15, 78, 79). LncRNAs are defined by their length and lack of an open reading frame (ORF) capable of encoding a protein. Despite their non-coding nature, lncRNAs are involved in crucial regulatory functions within the cell. They are classified based on their proximity to protein-coding genes into several categories: sense, antisense, bidirectional, intronic, and intergenic lncRNAs. Each class has distinct mechanisms of action and functional implications in gene regulation, highlighting the diversity within this group of RNAs (14, 79–83).

The primary components of lncRNAs include regulatory elements that enable their interaction with DNA, RNA, and proteins. These elements facilitate the formation of secondary and tertiary structures that are essential for their function. LncRNAs can act as scaffolds, guides, decoys, or enhancers, depending on their structure and the molecules they interact with. Their ability to fold into specific structures allows them to bind to various targets with high specificity, mediating a wide range of biological processes (14, 84, 85). LncRNAs interact with DNA, RNA, and proteins to exert their regulatory functions. They can bind to DNA to form RNA–DNA hybrids, affecting the accessibility of the DNA to transcription factors and other regulatory proteins. By interacting with RNA molecules, lncRNAs can influence the splicing, stability, and translation of mRNAs. Their interaction with proteins can modulate the activity, localization, and interaction of proteins with other biomolecules. These interactions enable lncRNAs to act as key regulators of gene expression, mediating both activation and repression of gene transcription (86, 87). The impact of lncRNAs on gene expression is profound and varied. They can modulate gene expression at multiple levels, including chromatin remodeling, transcriptional control, and posttranscriptional processing. LncRNAs can recruit chromatin-modifying enzymes to specific genomic loci, leading to alterations in chromatin state and gene expression. At the transcriptional level, they can enhance or inhibit the assembly of transcriptional machinery at gene promoters (88, 89). Posttranscriptionally, lncRNAs can affect mRNA splicing, stability, and translation, further influencing gene expression outcomes. LncRNAs play a pivotal role in the regulation of gene expression, acting through diverse mechanisms to modulate the cellular transcriptome. Their ability to interact with DNA, RNA, and proteins enables them to exert broad regulatory functions, influencing gene expression at multiple levels. The complexity of lncRNA-mediated regulation highlights the intricate network of interactions that govern cellular processes, including those involved in cancer development and progression. In the context of cancer, lncRNAs have been implicated in various aspects of tumor biology, including proliferation, apoptosis, metastasis, and drug resistance. Their involvement in cancer-related pathways underscores the potential of lncRNAs as biomarkers for cancer diagnosis, prognosis, and therapeutic targets (87, 90, 91). LncRNAs are crucial for both innate and adaptive immune responses. The innate immune system serves as the initial barrier against infections, encompassing innate immune molecules and immune cells produced from myeloid cells. The adaptive immune response is a complex system that safeguards the host organism and provides protection against future encounters with the same pathogen. This response involves the activation of B and T cells. **Table 3** contains a compilation of research that has reported the presence of both innate and adaptive lncRNAs.

**Table 3:** Key lncRNAs and their roles in innate and adaptive immune responses.

Immunity type	lncRNA	Function	References
**Innate**	AS-IL-1α	Regulates IL-1α transcription	(92)
	IL-1b-RBT46	Acts in IL-1 and regulates LPS production by proinflammatory agents such as IL-1β and CXCL8	(93)
	IL7-AS	Regulates IL-6 expression	(94)
	Lethe	Binds to the active NF-kB subunit p65 (RelA) and reduces the production of inflammatory proteins like IL-6, IL-8, and superoxide dismutase 2 (SOD2), regulates NF-kB-mediated inflammatory genes	(95)
	FIRRE	Regulate the expression of several inflammatory genes at the posttranscriptional level through interaction with hnRNPU	(96)
	H19	Maintains hematopoietic stem cells quiescence	(97)
	LincRNACOX2	Regulates the expression of inflammatory genes by binding with hnRNP-A/B and A2/B1, regulates the expression of NF-kB and several immune genes, represses the transcription of IL-12b in response to TNF-α stimulation	(98)
	Lnc-DC	Promotes phosphorylation and activation of STAT3 and regulates DC differentiation (blocking dephosphorylation by SHP1)	(99)
	LincRNA-EPS	Inhibits IRG expression and represses the inflammatory response	(100)
	NKILA	Binds to the NF-κB/IkB complex and suppresses NF-κB signaling and cancer-related inflammation by inducing LPS, TNF-α, and IL-1β	(101)
	Lnc-IL7R	Regulates the expression of the inflammatory mediators IL-6, IL-8, E-selectin, and VCAM-1	(102)
	LncITPRIP-1	Regulates the innate immune response through the promotion of oligomerization and activation of MDA5	(103)
	Lnc-Lsm3b	Inactivates RIG-1 innate activity and type I IFN production	(104)
	LncRNA-Mirt2	Regulates macrophage polarization and aberrant inflammatory activity	(105)
	LincRNA-Tnfaip3	Regulates the expression of several NF-kB inflammatory genes	(106)
	MALAT1	Regulates the expression of inflammatory genes Regulates LPS-mediated M1 macrophage activation and IL-4-mediated M2 differentiation and interaction with NF-kB	(107)
	MIR3142HG	Regulates CCL2 and IL-8 mRNA, and downregulates MIR3142HG/mir146a interaction	(108)
	NEAT1	Binds to SFPQ and regulates IL-8 expression	(109)
	LncHSC-1/2	LncHSC-1 regulates myeloid differentiation and LncHSC-2 cell self-renewal and differentiation	(110)
	NRIR	Regulates the expression of several interferon-stimulated genes and protein release of CXCL10 and CCL8	(111)
	PACER	Promotes COX2 expression and interacts with the repressive NF-κB subunit p50	(112)
	THRIL	Regulates the expression of the innate-associated mediators TNF-α, CCL1, IL-8, CSF1, and CXC10; interacts with hnRNPL to establish a practical THRIL-hnRNPL complex	(113)
	HOTAIRM1	Regulates RA-induced HOXA1 and HOXA4 expression during RA-induced NB4 cell granulocyte differentiation and promotes CD11b and CD18 expression	(114)
	lncRNA-CMPK2	Modulation of the IFN response and upregulated in HCV-infected human livers	(115)
	Morrbid	Regulates the lifespan of neutrophils, eosinophils, and monocytes by suppressing transcription of Bcl2l11	(116)
**Adaptive**	lncRNA-CD244	Inhibits the expression of IFN and TNF downstream of CD244	(117)
	Fas-AS1	Increases Fas/FasL-mediated apoptosis in B and T-cells and other cell types	(118)
	lncRNA-RMRP	As a key DDX5-associated RNA required to promote and regulate the function of RORγt transcriptional complexes in the T_H_17 effector program (direct correlation with IL-17a)	(119)
	FLICR	Overlaps with FoxP3 and is specifically expressed in Tregs and negatively regulated by IL-2	(120)
	NRON	Regulates IL-2 expression in T_H_1 cells and is associated with NFAT-dependent genes	(121)
	NTT	Regulates inflammation in monocytes and participates in monocyte/macrophage differentiation	(122)
	GAS5	Involved in the mTOR and GCR pathway	(123)
	GATA3-AS	Coexpressed with GATA3 under T_H_2-polarizing conditions	(124)
	NeST	Promote IFN-γ expression in downstream T_H_1 cells of NF-kB, Ets1, STAT4, and T-bet	(125)
	TH2-LCR	Regulates the expression of the IL-4, IL-5, and IL-13 gene cluster in T_H_2 cell cytokines	(126)
	lincR-Ccr2–5′AS	Controls a T_H_2-specific gene expression program such as Ccr1–2-3–5	(92)
	Linc-MAF-4	Promotes T_H_1 differentiation by repressing expression MAF	(93)

### 3.1 LncRNAs in cancer

LncRNAs have emerged as key players in the regulation of cancer development and progression. Their diverse roles in modulating gene expression, cellular processes, and signaling pathways have been implicated in various aspects of cancer biology, including proliferation, apoptosis, metastasis, and drug resistance. The involvement of lncRNAs in these processes highlights their potential as biomarkers for cancer diagnosis, prognosis, and therapeutic targets. LncRNAs have been shown to modulate cellular proliferation and apoptosis, two critical processes in cancer development. For example, the lncRNA HOTAIR has been implicated in the regulation of tumor growth and metastasis by modulating the expression of tumor suppressor genes, such as PTEN. Another lncRNA, MALAT1, has been linked to the promotion of cell proliferation and inhibition of apoptosis in various cancer types. These findings underscore the potential of lncRNAs as key regulators of cellular processes in cancer. Researchers have also implicated lncRNAs in the regulation of cancer metastasis and drug resistance. For instance, studies have shown that the lncRNA MEG3 inhibits cancer cell migration and invasion by modulating the expression of genes related to metastasis. Similarly, the lncRNA HOTAIR has been associated with the development of drug resistance in cancer cells by regulating the expression of drug resistance-related genes. These findings suggest that lncRNAs play a crucial role in the progression of cancer and the development of therapy resistance (8, 10, 85, 90, 127). The involvement of lncRNAs in various cancer-related processes has led to their consideration as potential therapeutic targets and biomarkers. For example, the lncRNA HOTAIR has been proposed as a potential therapeutic target for the treatment of breast cancer, as its inhibition has been shown to reduce tumor growth and metastasis in preclinical models. Additionally, lncRNAs have been suggested as biomarkers for cancer diagnosis and prognosis, as their expression levels have been found to correlate with cancer progression and patient outcomes (127, 128). LncRNAs involved in different cancers and their various functions are listed in **Supplementary Table 3**.

## 4 LncRNAs and tumor immune microenvironment

The TIME has several constituents, including the extracellular matrix, blood vessels, immune cells, and signaling molecules. Various patterns of immune infiltration and activation are applied in various tumors by the dynamic and varied interactions between these constituents (129). Certain cancers have a high degree of immune cell infiltration and activation, meaning that a large number of immune cells are present to identify and combat the cancer cells. These tumors are referred to as immunological hot or inflammatory tumors, and they typically respond better to immunotherapy. As a result, some types of cancer do not have many or any immune cells that can kill the cancer cells. These tumors are called immunological cold or non-inflammatory tumors, and they do not respond well to immunotherapy. However, they may respond well to other treatments that boost the immune system, such as radiation, chemotherapy, or targeted therapy (129). Numerous elements affect the TIME, including the host immune system, the environment, the kind and location of the tumor, the genetic and epigenetic properties of the cancer cells, and more. Understanding the TIME and its function in tumor advancement and regression is essential for creating efficient and customized cancer therapies (130). LncRNAs have the potential to be both therapeutic targets and prognostic indicators for cancer, and they can also play significant roles in the TIME. Unlike mRNAs, the unique subcellular localizations and activities of lncRNAs are linked to their synthesis. LncRNAs can affect chromatin function, control the stability and translation of cytoplasmic mRNAs, and obstruct signaling pathways, depending on where they are located and how they specifically interact with DNA, RNA, and proteins. LncRNAs play a role in immunological responses, immune cell activation, and antigen presentation, among other immune-related processes (131).

### 4.1 Myeloid cells

Myeloid cells are a particular type of immune cell that are essential to the cancer TIME (132). They comprise granulocytes, macrophages, dendritic cells, and monocytes that can be produced from either monocytes or neutrophils. Myeloid cells may either stimulate or repress the immune system, which can have a beneficial or negative effect on the spread and metastasis of cancer (133). Myeloid cell accumulation is linked to poor outcomes and resistance to treatments like ICIs and chemotherapy (134). Clinical investigations targeting myeloid cells in cancer are currently in process, despite the little achievement made in huge-scale clinical studies employing myeloid cell modulators. Nonetheless, novel approaches are being developed to target tumor-resident myeloid cells with cancer immunotherapy. In both preclinical and clinical settings, several tactics aimed at the myeloid compartment consist of various strategies (132). One strategy is to modify the myeloid population around a tumor by increasing myeloid cell recruitment, differentiation, and proliferation inside the TIME. The CCL2-CCR2 pathway plays a major role in the activation of myeloid cells, such as TAMs and MDSCs, and is associated with inflammation monocytes (135).

Researchers have identified several lncRNAs as regulators of MDSC function and differentiation. MDSCs from lung cancer patients upregulate the lncRNA HOTAIR, which stimulates MDSC expansion and immunosuppressive activity by sponging miR-124. Retinal non-coding RNA3 (RNCR3) is highly expressed in MDSCs from colorectal cancer. It works with miR-185–5p to improve their immune-suppressing effects. In AML-derived MDSCs, LncRNA RUNXOR is overexpressed and contributes to their expansion by regulating RUNX1 expression (136). Key lncRNAs influencing TAM polarization and function include lncRNA NIFK-AS1, which is upregulated in M2-like TAMs and promotes their polarization by sponging miR-146a. In colorectal cancer, LINC00662 enhances M2 polarization of TAMs by regulating the miR-17–5p/STAT3 axis (136). LncRNA GAS5 blocks the miR-222–3p/PTEN axis, which stops M2 macrophage polarization and tumor metastasis (137). Research has also revealed lncRNAs that affect other myeloid cell types within the TIME. LncRNA NEAT1 regulates the differentiation and function of dendritic cells by modulating miR-3076–3p expression. By controlling the expression of CXCL1, HOTAIR affects the migration and activation of neutrophils in hepatocellular carcinoma (136, 137).

A preclinical study indicates that polarized macrophages and elevating the M1 to M2 ratio can reduce myeloid-driven immunosuppression by causing DC stimulation, CD8+ T-cell triggering, and increased phagocytosis when the CD47/SIRPα pathway is inhibited (138). Turning myeloid cells into pro-inflammatory ones by reprogramming them is another way. As an example, TLR9 appears constitutively in the endosomes of pDCs and B cells, but it is also expressed by other myeloid categories in response to immunological stimuli, such as infection. Both innate and adaptive immune cells are highly stimulated by MyD88 signaling when TLR9 identifies unmethylated cytosine-phosphate guanine (CpG) oligodeoxynucleotides (ODNs) on modified or uncommon DNA. Scientists have used this finding to design TLR9 agonists that are based on CpG ODNs. The aforementioned stimulants have shorter half-lives by nature because they are physiologic derivatives; however, by adding a nuclease-resistant phosphonothioate foundation, the half-lives of these agents have been extended from minutes to days (SD-10, CPG 7909, ISS 1018, S-540956, IMO-2055, tilsotolimod, GNKG168, and CpG-28) (139). Another strategy is to manipulate myeloid cells through cytokine activity to enhance antitumor immunity. This tactic affects the function of myeloid cells, which are cells such as TAMs and cells called dendritic cells, by using cytokines. For instance, it has been demonstrated that IL-12 accelerates the growth of dendritic cells and boosts the effectiveness of immunotherapy (140). Utilizing myeloid cells as therapeutic agents to enhance antitumor immunity, referred to as myeloid cell treatments, is also another strategy. For example, vaccines based on DCs have shown promise in enhancing immunity to tumors and enhancing the efficacy of immunotherapy (141). **Table 4** provides information on lncRNAs that affect myeloid cells within the TIME.

**Table 4:** Regulatory roles of lncRNAs in modulating myeloid cells within the tumor immune microenvironment.

LncRNA	Role in TIME	Impact on myeloid cells	Associated cancer types	Mechanism of action	Potential therapeutic targets	References
MALAT1	Promotes tumor progression, metastasis, and angiogenesis	Suppresses T-cell responses, induces MDSC expansion and activity	Lung, colorectal, gastric, breast, bladder	Sponges miR-124a, regulates EMT and stemness pathways	Yes, ongoing research on MALAT1 inhibitors and antisense oligonucleotides	(142)
MEG3	Suppresses tumor growth and invasion, inhibits metastasis	Inhibits MDSC expansion and promotes M1 macrophage polarization	Liver, gastric, bladder, breast	Downregulates TGF-β signaling, interacts with p53	Yes, potential for MEG3 mimics or agonists as cancer therapy	(143)
HOTAIR	Promotes tumor proliferation, migration, and invasion	Induces MDSC expansion and M2 macrophage polarization, suppresses T-cell responses	Breast, liver, colorectal, gastric	Acts as a scaffold for protein complexes, regulates WNT signaling pathway	Yes, potential for HOTAIR inhibitors or siRNA silencing	(144)
GAS5	Suppresses tumor growth and metastasis, promotes apoptosis	Inhibits MDSC expansion and promotes M1 macrophage polarization	Lung, prostate, renal, breast	Binds to glucocorticoid receptor, modulates NF-κB signaling	Yes, potential for GAS5 mimics or activators as cancer therapy	(145)
H19	Promotes tumor growth, invasion, and angiogenesis	Induces MDSC expansion and M2 macrophage polarization, suppresses NK cell activity	Gastric, lung, colorectal, ovarian	Interacts with various proteins, including EZH2 and PTEN, modulates PI3K/AKT signaling	Yes, potential for H19 inhibitors or siRNA silencing	(146)
UCA1	Promotes tumor progression and metastasis, inhibits apoptosis	Induces MDSC expansion and M2 macrophage polarization, suppresses T-cell responses	Bladder, liver, gastric, lung	Sponges miR-1, regulates BCL-2 expression and EMT	Yes, potential for UCA1 inhibitors or antisense oligonucleotides	(147)
SPRY4-IT1	Promotes tumor growth and metastasis, induces chemoresistance	Inhibits dendritic cell maturation and antigen presentation, suppresses T-cell responses	Lung, gastric, pancreatic, colorectal	Interacts with hnRNP-A2B1, modulates alternative splicing events	Yes, potential for SPRY4-IT1 inhibitors or splicing modulators	(148)
PVT1	Promotes tumor proliferation and invasion, inhibits apoptosis	Induces MDSC expansion and M2 macrophage polarization, suppresses NK cell activity	Liver, hepatocellular carcinoma, renal, colon	Activates c-Myc signaling, suppresses p53 expression	Yes, potential for PVT1 inhibitors or antisense oligonucleotides	(149)
SNHG12	Promotes tumor growth and metastasis, inhibits apoptosis	Induces MDSC expansion and M2 macrophage polarization, suppresses T-cell responses	Lung, gastric, esophageal, colorectal	Sponges miR-199a/b-5p, regulates PI3K/AKT signaling pathway	Yes, potential for SNHG12 inhibitors or antisense oligonucleotides	(150)
XIST	Promotes tumor progression and metastasis, induces angiogenesis	Inhibits dendritic cell maturation and antigen presentation, suppresses T-cell responses	Breast, ovarian, endometrial, liver	Silences tumor suppressor genes through X chromosome inactivation	Yes, potential for XIST inhibitors or reactivation of silenced genes	(151)
LINC00673	Promotes tumor growth and metastasis	Induces MDSC expansion and M2 macrophage polarization	Lung, liver, breast	Interacts with EZH2, regulates WNT signaling	Yes, potential for LINC00673 inhibitors or siRNA silencing	(152)
SNHG16	Promotes tumor invasion and angiogenesis	Suppresses T-cell responses, induces MDSC recruitment	Gastric, colorectal, pancreatic	Sponges miR-200 family, regulates EMT and stemness pathways	Yes, potential for SNHG16 inhibitors or antisense oligonucleotides	(153)
BANCR	Promotes tumor proliferation and migration	Induces MDSC expansion and M2 macrophage polarization	Breast, liver, lung	Interacts with STAT3, regulates JAK/STAT signaling pathway	Yes, potential for BANCR inhibitors or siRNA silencing	(154)
NEAT1	Promotes tumor progression and metastasis	Suppresses T-cell responses, induces MDSC expansion	Lung, colorectal, breast	Sponge various miRNAs, regulates multiple signaling pathways	Yes, potential for NEAT1 inhibitors or miRNA mimics	(155)
ZFAS1	Promotes tumor growth and invasion	Inhibits dendritic cell maturation and antigen presentation	Liver, gastric, pancreatic	Interacts with hnRNPA1, modulates alternative splicing events	Yes, potential for ZFAS1 inhibitors or splicing modulators	(156)
SOX2OT	Promotes tumor stemness and chemoresistance	Induces MDSC expansion and M2 macrophage polarization	Breast, ovarian, lung	Regulates SOX2 expression, interacts with WNT signaling pathway	Yes, potential for SOX2OT inhibitors or siRNA silencing	(157)
CRNDE	Promotes tumor proliferation and metastasis	Suppresses T-cell responses, induces MDSC recruitment	Liver, colorectal, gastric	Sponges miR-34a, regulates cell cycle and apoptosis pathways	Yes, potential for CRNDE inhibitors or antisense oligonucleotides	(158)
Linc-ROR	Promotes tumor invasion and metastasis	Inhibits NK cell activity, suppresses T-cell responses	Lung, breast, colorectal	Interacts with various proteins, including YAP1 and TGF-β receptor	Yes, potential for Linc-ROR inhibitors or interfering peptides	(159)
MIR155HG	Promotes tumor growth and angiogenesis	Induces MDSC expansion and M2 macrophage polarization	Liver, lung, gastric	Sponges miR-145 and miR-152, regulates NF-κB signaling pathway	Yes, potential for MIR155HG inhibitors or miRNA mimics	(160)
HOTAIRM1	Promotes tumor proliferation and migration	Induces MDSC expansion and M2 macrophage polarization	Breast, liver, colorectal	Interacts with EZH2 and PRC2 complex, regulates gene expression	Yes, potential for HOTAIRM1 inhibitors or EZH2 inhibitors	(161)

### 4.2 Macrophages

One subset of white blood cells that are a component of the body’s innate immune system are macrophages. They are essential for the process of phagocytosis, which involves engulfing and breaking down infections, cancer cells, cellular debris, and foreign objects without proteins unique to healthy body cells on their surface. Macrophages are incredibly malleable cells that may take on a range of activation states in reaction to environmental cues. They play a role in the development of allergy and autoimmune illnesses, non-infectious inflammatory diseases, and the genesis and resolution of sterile inflammation (162). Developing efficient therapies requires an understanding of the function of macrophages in cancer immunotherapy. Macrophages are intricately involved in the immune system’s reaction to malignancies, and a high number of macrophages in tumors are frequently linked to a bad prognosis (163). Macrophages have the ability to affect carcinogenesis in two ways: either by strengthening immune cell responses against tumors or by opposing their cytotoxic effects. Clinical research and experimental models have investigated the targeting of macrophages in cancer, indicating its promise as a therapeutic approach (164). Advances in cancer nano-immunotherapies have also highlighted the function of macrophages within the TIME. An analysis demonstrated the critical function of M1 macrophages in the TIME proinflammatory processes and antitumor immunity. The importance of TAMs in medication resistance, including immunotherapies and cancer prognosis, is emphasized in the study. It also emphasizes how nanoparticles are beginning to be used to target TAMs in order to safely and effectively boost immune responses and sensitize tumors to immunotherapies (165). The distinct functions that M1 and M2 macrophages play in the development of cancer have been extensively studied. These functions are crucial to comprehending the evolution of the TIME. M2 macrophages have a tendency to promote angiogenesis, neovascularization, stromal activation, and remodeling, which can have a favorable or negative influence on cancer development and patient prognosis. M1 and M2 macrophages are both engaged in tumor-related inflammation (166). An investigation explores the functions of M1-like and M2-like macrophages in BC. It highlights that whereas M2-like macrophages are linked to angiogenesis, immunological suppression, and tissue repair, which promote tumor growth, M1-like macrophages exhibit antitumor action by causing inflammation. The study also highlights the M1/M2 macrophage ratio’s clinical significance and the possibility of transforming M2-like macrophages into antitumoral M1-like macrophages, demonstrating the adaptability of macrophages in the TIME (167). The effects of M1 and M2 macrophages on the development of lung cancer were discovered in another investigation. The study comes to the conclusion that M1 and M2 macrophages affect lung cancer cells’ ability to regulate their biological activities and gene expression differently. The M1/M2 macrophage balance in the TIME is important for both patient survival and cancer development, and the study indicates that this balance may be used as a prognostic indication for patients with lung cancer (168). It has been determined that lncRNAs, such as p21 lncRNA, play a crucial role in mammography as they regulate TAMs. This highlights the possibility of using lncRNAs as targets for future cancer immunotherapy biomarkers and therapies. In addition, it emphasizes how lncRNAs influence the TIME and regulate the immune escape of tumor cells, suggesting a bright future for lncRNA-based targeted cancer immunotherapy (169).

### 4.3 Myeloid-derived suppressor cells

Myeloid-derived suppressor cells (MDSCs) promote immunosuppression, stimulate tumor development, and establish a pre-metastatic niche, all of which are important factors in the course of cancer. These cells are pathologically activated neutrophils and monocytes with strong immunosuppressive activity, and they are produced from hematopoietic stem cells in the bone marrow (170). MDSCs stop T cells from activating and working in a number of ways, such as by making ligands for negative regulators of T-cell activity. It has been noted that they inhibit both innate and adaptive immunity and that those with cancer have higher concentrations of them in their bloodstreams and bone marrow. MDSCs have also been shown to make cancer cells more stem-like by increasing miR-101 and decreasing the activity of the corepressor CtBP2 (171). To develop new approaches for tumor immunotherapy, it is essential to comprehend the mechanisms underlying MDSC generation, expansion, recruitment, and activation (172). Therapeutic interventions targeting MDSCs could be possible, as recent research has confirmed that these cells suppress the immune system and boost cancer through multiple pathways (173). The variety of MDSCs, such as their monocytic (M-MDSCs), granulocytic (PMN-MDSCs), and early forms, emphasizes their distinct position within the myeloid lineage and the intricacy of their function in the advancement of cancer (170). It has also been shown that MDSCs actively take part in immune evasion, angiogenesis, the creation of pre-metastatic niches, the EMT, and additional facets of tumor growth. Their non-immunological actions enhance tumor invasion, and they contribute as well to generating an immunosuppressive environment that affects their own biology and function (174).

The following strategies are the major focus of current clinical therapies for MDSCs (1). Inhibition of MDSCs: This approach, either independently or in conjunction with other treatments, including radiation, chemotherapy, surgery, or immunotherapy, has demonstrated effectiveness in preclinical studies as well as clinical ones. Reversing immunological escape and optimizing immunological-based therapies are the goals of MDSC inactivation (175). One approach that shows promise for improving the effectiveness of cancer immunotherapy is focused on the reduction of MDSCs. In a number of cancer types, including melanoma, breast, colorectal, lung, and hematologic malignancies, high concentrations of MDSCs have been associated with poor prognoses, an increase in malignancy, and decreased therapeutic efficacy of immunotherapies (174, 175). To improve the therapeutic efficiency of cancer immunotherapy, one tactic being investigated is blocking the suppressive actions of MDSCs. It is well recognized that MDSCs suppress antigen-specific CD8+ T-cell activity, which results in a reduction in IFN-γ production (176). Another way to improve the therapeutic efficiency of cancer immunotherapy is to block the suppressive actions of MDSCs. MDSCs obstruct the efficacy of cancer immunotherapies by actively fostering an immune-tolerant TIME (177). MDSCs become stimulated in the TIME, and, through a variety of methods, such as blocking T cells’ ability to eradicate cancer, they encourage immunosuppression and tumor development. The expression of negative immune checkpoint molecules like PD-L1, a reduction of amino acids necessary for the activation of T lymphocytes, the generation of reactive oxygen species and nitric oxide, and the release of immune-suppressive cytokines like TGF-β and IL-10 are the main mechanisms by which MDSCs prevent T cells (178). The function of lncRNAs in the control of MDSCs in the TIME has been the subject of several investigations. In lung cancer patients, MALAT1 lncRNA has been demonstrated to adversely regulate MDSCs (179). *In vitro* and *in vivo* MDSC differentiation and function are both inhibited by the knockout of RNCR3lncRNA, which has been shown to be upregulated in MDSCs by both inflammatory and tumor-associated factors (20).

### 4.4 Neutrophils

White blood cells, known as neutrophils, are vital to the body’s immune system’s reactions. They comprise 50% to 70% of all human white blood cells and are among the most prevalent forms of granulocytes (180). Through a process known as phagocytosis, neutrophils are recognized for their capacity to absorb particles or germs. When bacterial infection, environmental exposure, or some malignancies occur, they are the first inflammatory cells to react and move toward the site of inflammation (181). Neutrophils have a variety of tasks, and their functional variability has been emphasized by a recent study. Numerous subpopulations of neutrophils have been identified so far, and studies have demonstrated that they display morphological and functional variation. This variety shows that neutrophils are a heterogeneous population that responds differently to diverse clinical situations and tissues. Moreover, data indicate the flexibility of mature neutrophils in circulation, suggesting that they can be reprogrammed in response to outside stimuli (182). Recent research has shed light on the role of neutrophils in cancer immunotherapy. Neutrophils accumulate in solid tumors, and their abundance correlates with a poor prognosis.

Neutrophils associated with poor prognosis in solid tumors and PMN-MDSCs are both important players in cancer progression, but they function differently within the tumor microenvironment. Both cell types suppress immune responses and promote tumor growth by infiltrating tumors and secreting growth factors. However, typical neutrophils can switch between tumor-inhibiting and promoting roles, whereas PMN-MDSCs are exclusively immunosuppressive, marked by specific surface molecules that regular neutrophils do not express. The primary difference between these cells is in their development and function. Neutrophils are a standard component of the immune system, potentially adopting antitumor or protumor roles (termed N1 and N2, respectively), influenced by the TIME. In contrast, PMN-MDSCs are aberrantly activated neutrophils with distinct markers like CD11b+, CD14−, CD66b+, and LOX-1, specifically arising under pathological conditions such as cancer, and are uniformly supportive of tumor growth due to their strong immunosuppressive capabilities (183).

According to research, interferon-elicited neutrophils developed an interferon gene signature, which is also observed in human patients. This signature was necessary for therapy to be successful because immunotherapy failed when neutrophils’ interferon-responsive transcription factor (IRF1) was lost. Important elements of antitumor immunity, such as IL-12, IFNγ, and DCs reliant on BATF3, were also necessary for the neutrophil response. Furthermore, they discovered that in lung cancer patients, a systemic neutrophil response induced by medication had a favorable correlation with the course of the illness (184). The treatment and prevention of cancer are being revolutionized by immune-mediated checkpoint blockade-based immunotherapies. Recent research has shown that tumor-associated neutrophils (TANs) play a critical role in regulating the TIME and the immune system’s fight against the tumor. However, these cells were mostly overlooked whereas ligand-1, the cell death program receptor, CTLA-4, and ICIs were being developed as treatment targets. Recent findings about the multifunctional variety of neutrophils in tumors have sparked a lot of debate and may open up new therapeutic avenues when it comes to the emergence of ICIs. Research also showed that a significant change in neutrophil biogenesis and function is linked to the development of cancer and has the potential to both anticipate and disrupt the immune system’s reaction to ICIs. Important characteristics of ICI resistance are also linked to neutrophil infiltration in malignancies. When taken with ICIs, TANs contribute significantly to antiangiogenic drug resistance, decreasing their therapeutic efficacy (185). LncRNAs have been studied recently in relation to cancer immunotherapy and neutrophils. An analysis of BC research, for instance, discovered that an lncRNA profile associated with neutrophil extracellular traps (NETs) might be used to predict prognosis as well as the immunological microenvironment and responsiveness to immunotherapy in BC (186). A further investigation on lung cancer found a lncRNA profile linked to NETs that connected with the TIME and anticipated patient clinical results. SIRLNT, AL365181.3, FAM83A-AS1, and AJ003147.2 are the four NET-associated lncRNAs that were discovered to be highly correlated with patient survival outcomes in the risk model (187). Working with neutrophils in immunotherapy has a number of difficulties and restrictions. Several obstacles and restrictions exist because, due to their immunosuppressive characteristics, neutrophils can be a factor in treatment resistance in a variety of solid tumors. This is a major obstacle to developing potent immunotherapies capable of overcoming this resistance (188). Also, both inflammation and immunological responses depend heavily on neutrophils. In order to effectively manipulate neutrophils for immunotherapy, it is important to strike a careful balance between maximizing their anticancer potential and preventing side effects from exaggerated inflammation or immune response. Additionally, different health and illness situations have been linked to the occurrence of many subpopulations of neutrophils. However, it is difficult to create focused medicines that can successfully modulate neutrophils since the variations in plasma membrane proteins and functional responses of neutrophils under various circumstances are not well known (181).

### 4.5 Lymphocytes

The production of antigen-specific lymphocytes by host organisms in response to subsequent exposure to comparable antigens or reinfection with the same pathogen is called adaptive immune responses. B cells and T cells constitute the adaptive immune system (189).

### 4.6 B cells

**Figure 2:**
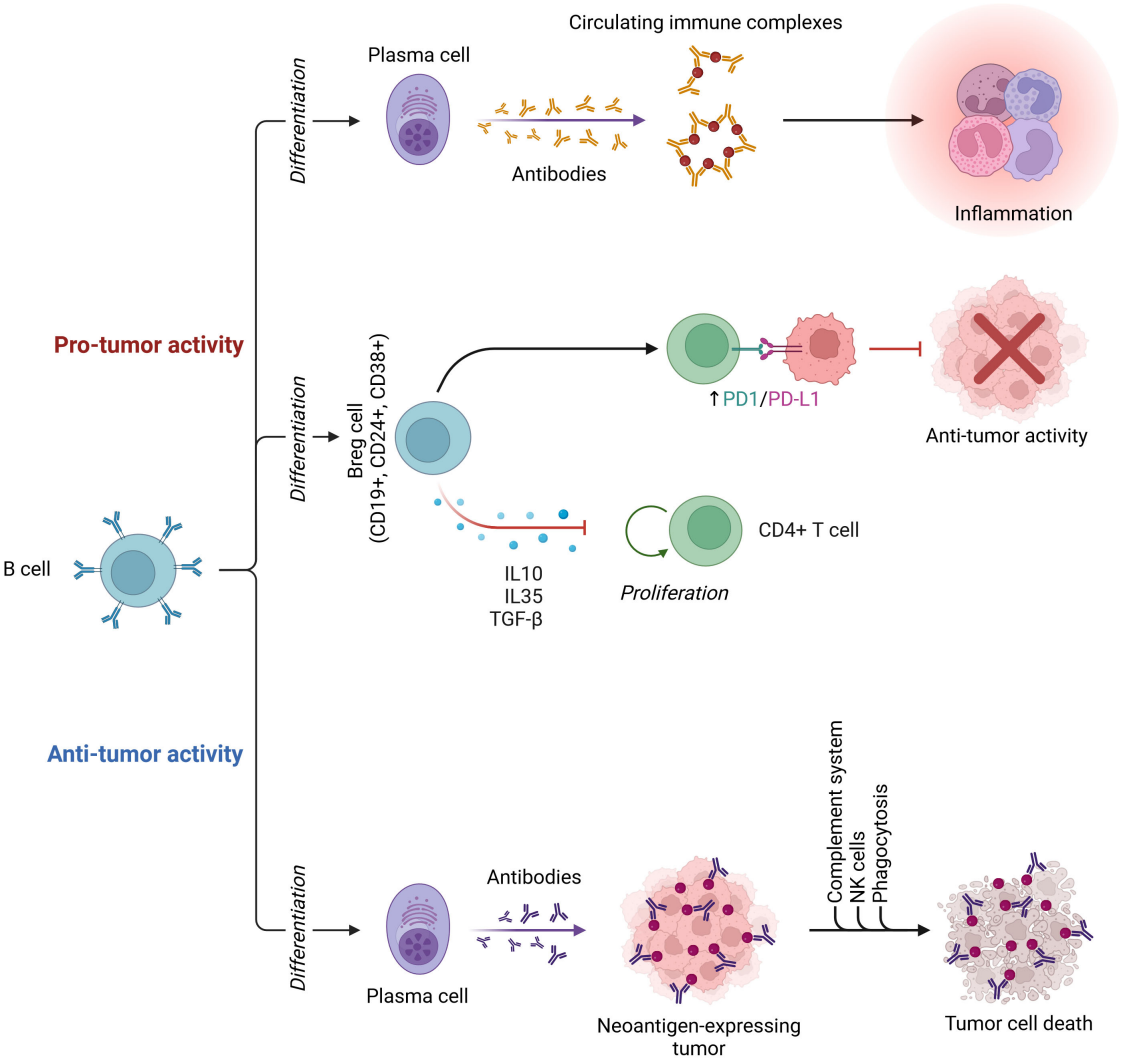
Antitumor and protumor activity of B-cells in the cancer tumor microenvironment: B cells can differentiate into plasma cells, which produce antibodies that target neoantigen-expressing tumors, leading to tumor cell death through the complement system, NK cells, and phagocytosis, thus exhibiting antitumor activity. Conversely, B cells can differentiate into regulatory B cells (Bregs, CD19+, CD24+, CD38+), which secrete IL-10, IL-35, and TGF-β, promoting CD4+ T-cell proliferation and upregulating PD-1/PD-L1, ultimately inhibiting antitumor activity. Additionally, plasma cells can contribute to protumor activity by producing antibodies that form circulating immune complexes, leading to inflammation (this image was created with BioRender).

B cells are essential in the activation of humoral immune responses. B cells are also engaged in initiating T-cell immunological responses, which are required for the control of immune homeostasis (190). Tumor-infiltrating immune cells serve a crucial function in promoting or suppressing tumor growth. Tumor-infiltrating B lymphocytes (TIBs) are found throughout all stages of cancer and contribute to tumor formation as part of the TIME (191). **Figure 2** depicts the dual roles of B cells in the cancer tumor microenvironment, highlighting their capacity for both antitumor activity through plasma cell differentiation and tumor cell death, and protumor activity via regulatory B cells and inflammation. TIBs have been linked to tumor immunity in a variety of ways. On the one hand, B cells have been seen invading several malignancies and producing class-switched affinity-matured anticancer antibodies *in situ* (192). TIBs also operate as antigen-presenting cells, promoting T-cell-mediated immune responses and reinforcing antitumor immunity (193). TIBs, on the other hand, have been identified as regulatory B cells (B-regs) and have been found to enhance cancer progression. B-regs showed their immunosuppressive properties in human and mouse studies by secreting death ligands (TRAIL, FasL) or anti-inflammatory molecules (IL-10, TGF), which can disrupt NK and T-cell responses and promote the protumoral actions of Tregs, MDSCs, and TAMs, reducing antitumor immune responses (193). As measured by the TIME, many factors influence B-cell homeostasis, including intratumoral vascularity, inflammatory degree, cytokines, hypoxia, and cellular infiltration. B cells may be split toward pro- or antitumor orientation once that balance is disrupted. Unfortunately, there have been few studies on lncRNAs and B cells in the tumor environment to date. The majority of them have focused on B-cell lymphomas triggered by lncRNA dysfunction during development and maturation, such as Burkitt lymphoma (BL), classical Hodgkin lymphoma (cHL), and diffuse large B-cell lymphoma (DLBCL) (194). A systematic analysis of the poly-adenylated transcriptome of 116 primary DLBCL specimens identified 2,632 novel lncRNAs in DLBCL, expanding the lymphoma transcriptome (195). This suggests that lncRNAs may play a role in lymphoma development. By modulating the miR-497–5p-mediated expression of the target gene PIM, LncRNASNHG16 promotes lymphoma cell proliferation and inhibits apoptosis (196). TUG1 lncRNA depletion inhibits tumor growth both *in vitro* and *in vivo* by inducing MET ubiquitination and subsequent elimination (197). Numerous lncRNAs can influence tumor development by promoting immune evasion. By regulating the PD-1/PD-L1 checkpoint, SNHG14/miR-5590–3p/ZEB1 generates a positive feedback loop that supports immune evasion and lymphoma development (198). MALAT1 promotes lymphoma immune evasion by targeting miR-195. MALAT1 silencing promotes CD8+ T-cell proliferation while inhibiting EMT-like processes via the Ras/ERK signaling pathway (199). MYC, a well-known transcription factor that is considered to be the primary driving force in the development of lymphoma, is activated and regulated by many of them (200). For example, MINCR lncRNA controls the expression of cell cycle genes such as AURKA, AURKB, and CDT1 by regulating the MYC transcriptional pathway (201). The miR-34b-5p/GLI1 axis promotes lymphomagenesis and B-cell proliferation through MYC-induced NEAT1 lncRNA (202). MYC-triggered FIRRE lncRNA promotes lymphoma progression by controlling β-catenin nuclear translocation, which activates the Wnt/-catenin pathway (203). MYC is not the only modulator that interacts with a lncRNA’s promoter region to boost expression. The MAPK/ERK signaling pathway is inactivated by P53-induced PANDA lncRNA, which reduces tumor growth in human lymphoma (204). By activating the Wnt/-catenin signaling pathway, the FOXM1-regulated overexpression of OR3A4 lncRNA controls cell death and proliferation (205). Aside from OR3A4 and FIRRE, the SMAD5-AS1 lncRNA inhibits lymphoma growth via the classic Wnt/-catenin pathway; however, APC expression is required (206). In patients with B-cell lymphoma, a range of lncRNAs are connected with prognosis and have an impact on therapeutic treatment. HULC, HOTAIR, LUNAR1, and PEG10 are lncRNAs that potentially predict a poor clinical outcome, indicate neoplastic activity, and have potential diagnostic value in DLBCL (207). MALAT1 affects DLBCL chemotherapy sensitivity by influencing autophagy-related proteins such as LC3-II/LC3-I, p62, and ATG5 (208). An artificially designed i-lncRNA was described that targeted 13 oncogenic miRNAs based on complementary sequences. *In vitro* and *in vivo*, the i-lncRNA inhibited tumor growth by consuming a significant number of oncogenic miRNAs, primarily targeting a set of regulatory genes such as PTEN, TIMP3, p38/MAPK, and c-MYC. Furthermore, the soluble Fas decoy receptor (sFas) can block Fas-ligand-induced apoptosis. FAS-AS1 lncRNA, which is regulated by EZH2-mediated histone methylation, is a new regulator of sFas expression that influences cell apoptosis in non-lymphomas Hodgkin’s by functioning as a decoy for RBM5 (117).

### 4.7 T cells

T lymphocytes boost immune cell activation to tackle malignant cells. T cells also signal other immune cells to engage in immunological responses. Numerous types of T cells cause cancer cells to die and stop them from spreading. CTLs are immune cells that have CD8+ surface expression on their surface. These CTLs participate in the apoptosis of specific cells by delivering digestive enzymes or cytotoxic cytokines in granules (TNF and IFN) (209). Cancer cells have been shown to resist the effects of CTLs by rearranging cellular function via the expression of coding RNAs and lncRNAs, resulting in a change in the cellular response to the native immune system. LncRNAs have also been shown to have a role in cancer resistance to CTL activity in recent studies (88, 210). NEAT1 is a nuclear paraspeckle-localized lncRNA found in a variety of cancers (211). *In vivo* studies revealed that inhibiting NEAT1 reduces CD8+ T-cell death and promotes active cytolytic function via the miR-155/Tim-3 pathway, resulting in increased immunological activity (212). Ji et al. published a paper in 2018 about the involvement of Lnc-Tim-3 in the regulation of Tim-3 protein expression in HCC. According to the researchers, an increase in Lnc-Tim-3 specifically binds to Tim-3 protein and blocks its interaction with Bat-3, suppressing downstream Lck/NFAT1/AP-1 signaling, resulting in nuclear localization of Bat-3 and increased p300-dependent p53 and RelA transcriptional activation of antiapoptotic genes such as MDM2 and Bcl2, allowing exhausted T cells to survive and proliferate (213). This research suggests that Lnc-TIM-3 lncRNA can be exploited as a target for T-cell exhaustion and proliferation. Increased expression of nuclear factor B (κB)-interacting NKILA lncRNA in cytotoxic T cells has also been linked to a poor prognosis in breast and lung cancer. However, knocking down NKILA inhibited tumor growth by boosting CTL-mediated activation-induced cell death, HDAC-mediated P300 nuclear localization, and calmodulin activation after calcium influx (214). The lincRNA MAF-4 is also involved in the development of the Thl response, which is mediated by the epigenetic silencing of the transcriptional factor MAF-4, which is mediated by the recruitment of chromatin modifiers such as polycomb group proteins (PRC1 and PRC2), which facilitates the deposition of H3K27me3 (transcriptional repression) by using the catalytic component partner, primarily EZH2. Lysine-specific demethylase 1 facilitates H3K4me3 (transcriptional activation) and H3K27me3 (gene regulatory regions) in a similar way (LSD1). As a result, lincRNA-MAF-4 interacts with the transcriptional repressors LSD1 and EZH2 to allow the H3K27me3 trimethylation mark at MAF’s promoter, effectively silencing its production (126). One way cancer cells impact immune suppression is by increasing the number of immunosuppressive tumor-infiltrating lymphocytes in the TIME (215). Tregs are one of the most potent and well-studied suppressive characteristics identified in the TIME. Treg cells may potentially play a key role in cancer’s immune evasion strategies, according to mounting data. Furthermore, new data suggest that tumor-derived lncRNA is essential for Treg maintenance and differentiation (216). For example, in HCC, lnc-EGFR plays a role in distinguishing Tregs and suppressing CTLs (217). Lnc-EGFR expression in Tregs was found to be strongly related to EGFR/Foxp3 expression and tumor development but inversely related to IFN-γ expression. Despite the fact that several studies have demonstrated that FOXp3 Tregs play a role in tumor growth, a recent study discovered that the two unique populations of Tregs, FOXp3 low and FOXp3 high, play different roles in tumor promotion (119). Another study discovered the involvement of FLICR lncRNA in controlling FOXp3 expression in Treg. The activation of the conserved noncoding sequence-2 (CNS-2) enhancer by suppressing FLICR has been linked to the enhancement of Foxp3-suppressive actions in Tregs via increased IL-2 production, followed by TCR signaling. Pei et al. (2018) established the interaction of SNHG1 lncRNA with miR-448 in the regulation of IDO expression in TILs to further understand the role lncRNA and miR interplay in IDO synthesis. The study found that miR-448 inhibits IDO expression, particularly in CD4+ TILs, but not in CD4+ cells in circulating peripheral blood (218). In contrast, SNHG1 sponges miR-448, resulting in an elevation of IDO expression in HCC cells. This implies that SNHG1 might be exploited as a possible therapeutic target for improving immunotherapy by decreasing lncRNA expression. Furthermore, it was shown that increased expression of SOX5 and SOX3 in the tongue and colon cancer inhibited T-cell function via upregulating IDO1 (219). A previous study also demonstrated that IDO1-mediated immunosuppression promotes tumor growth by increasing the number of Treg and MDSC regulatory cells.

### 4.8 Natural killer cells

Natural killer (NK) cells are innate immune lymphocytes that play a vital role in the host’s defense against viral infection and cancer monitoring (220). There is evidence of NK cell cytotoxicity toward adjacent cells displaying oncogenic transformation-associated surface markers (221). Furthermore, NK cells improve antibody response and T-cell activation, demonstrating that they play a significant role in tumor immunotherapy (222). Zhang et al. have discovered the lncRNA expression profile in human primary lymphocyte cells. They revealed that unique NK-specific lncRNAs are involved in the formation and function of NK cells (223). They discovered that the expression of an NK-specific lncRNA, lnc-CD56, correlates with the expression of the NK cell surface marker CD56. Further investigation has revealed that lnc-CD56 may significantly enhance CD56 expression and NK cell development in human CD34+ hematopoietic progenitor cells (223). Another study found that MEG3, GAS5, and several miRNAs influence NK cell killing effectiveness in various cancer types (224). Natural cytotoxicity receptors trigger the production of the IFNG-AS1 lncRNA, and IFNG-AS1 enhances IFN secretion in human NK cells (225). As a result, IFNG-AS1 is a type I immune response modulator in general.

LncRNA expression is tightly linked to the course of NK-related cancers. In cancer patients’ NK cells, the level of GAS5 lncRNA is lower. By sponging miR-544 to target RUNX3, GAS5 enhances NK cell cytotoxicity, IFN production, and the fraction of CD107a+ NK cells, enhancing the killing function of NK cells (224). Furthermore, by modifying miR-18a, GAS5 enhances the secretion of IFN and TNF and cytotoxicity of NK cells against GC (226). Exosomes from tumors include functional lncRNAs that NK cells can pick up and use for intercellular communication. Through miR-4485–5p driven upregulation of NKp46, a crucial natural cytotoxicity receptor, IFNβ-induced exosomal linc-EPHA6–1, strengthens NK cell cytotoxicity against tumor cells and Zika virus-infected tumor cells (227). As a result, the findings from previous research imply that lncRNAs play a significant role in the control of NK cells invading the TIME.

### 4.9 Dendritic cells

Dendritic cells (DCs) are antigen-presenting cells that help to initiate and modulate both innate and adaptive immune responses. As a result, interest is increasing in controlling DC activity to improve cancer immunotherapy efficacy (228). Tumor-associated typical DCs (cDCs) phagocytose apoptotic cancer cell debris and transport cancer-related antigens to the draining lymph node, exposing them to naive CD4+ or CD8+ T cells, priming and activating T cells (229). Even though DCs have a high potential for anticancer immunity, the TIME causes significant challenges because it disturbs normal DC functions to avoid immune surveillance and therefore obstructs the formation of protective immune responses (230). Neoplastic cells can develop a variety of mechanisms that allow them to thrive in extreme conditions. Numerous cytokines discovered in the tumor environment, for instance, can have a direct impact on the activity of infiltrating DCs, promoting malignant progression. For example, the STAT3-related pathway can be stimulated by IL-6, IL-10, and VEGF, commonly overexpressed in the TIME, causing an immature and tolerogenic phenotype in tumor-associated dendritic cells (TADCs) and thus promoting cancer progression (231). Indeed, immunosuppressive and tolerogenic DC populations are frequently found in the TIME of aggressive malignancies. LncRNAs modulate posttranslational modifications and interact with signaling molecules in the cytoplasm to influence cellular function and differentiation (99). The Lnc-DC lncRNA (gene symbol LOC645638) is only found in human Lin MHC-II+ CD11c+ conventional DCs. DC differentiation is inhibited *in vitro* and *in vivo* when Lnc-DC is silent, and DC’s ability to stimulate T-cell activation is reduced. By preventing STAT3 from binding to and dephosphorylating SHP1, Lnc-DC binds to STAT3 in the cytoplasm and stimulates tyrosine-705 phosphorylation (99). Some studies have found that Lnc-DC promotes DC maturation and inhibits trophoblast invasion without the involvement of CD4+ T cells. The p-STAT3 inhibitor can restore lnc-DC function by mediating MMP and TIMP expression (232). In preeclampsia, Lnc-DC causes excessive maturation of decidual DCs and an increase in Th1 cells (233). In addition, the TLR9/STAT3 pathway in DCs modulates the HBV-induced immune response and cellular turnover (234). According to recent research, lncRNAs can delicately regulate the local immune system by regulating DC infiltration, differentiation, and metabolism, as well as influencing other immune cells such as T cells. By using the NLRP3 inflammasome as a molecular decoy for miR-3076–3p, NEAT1 induces a tolerogenic phenotype in DCs (235). MiRNA let-7i modulates NEAT1 expression in the nucleus by interacting with the transcription factor E2F1, which affects the H3K27ac distribution in the NEAT1 promoter. Infusions of NEAT1-depleted DCs reduced the infiltration of inflammatory cells, increased the number of Tregs, and slowed the proliferation of T cells in mouse models (235). Immune tolerance is generally induced as a result of these changes. HOTAIRM1 lncRNA is essential for myeloid development. When monocytes become DCs, their expression of HOTAIRM1 decreases (236). The level of some monocyte differentiation markers, such as B7H2 and CD14, is altered when HOTAIRM1 is knocked out. Furthermore, targeting HOTAIRM1 and HOXA1, a DC differentiation repression gene, miR-3960 acts as a competing endogenous RNA to regulate DC differentiation (236). The chemokine receptor CCR7 induces Lnc-Dpf3, which inhibits DC migration by preventing HIF-1-mediated glycolysis (237). CCR7 stimulation increased lncDpf3 by removing the m6 A modification that prevents RNA degradation. Lnc-Dpf3 knockdown promotes CCR7-mediated DC migration, which worsens inflammation and adaptive immune responses. Lnc-Dpf3 suppresses DC glycolytic metabolism and migration by preventing HIF-1-dependent transcription of the glycolytic gene LDHA (237). As a result, there is a hypothesis that decreasing lnc-Dpf3 in DCs can boost DC recruitment into the hypoxic TIME via a similar mechanism, enhancing adaptive immune responses and improving the TIME. In human colon cancer tissues, tumor-infiltrating DCs release a considerable amount of CCL5 (238). The MALAT1 lncRNA deficiency inhibits CCL5-mediated invasion and migration by lowering Snail levels (238). These results show that MALAT1/Snail signaling is required for TADC-mediated tumor development.

## 5 LncRNAs as potential cancer immunotherapeutic targets

Cancer comprises a diverse set of life-threatening diseases caused by abnormal and invasive cell proliferation. Cancer patients around the world are getting treatment with traditional methods, which are surgery, radiation, and cytotoxic drugs. While some patients have achieved cures, a significant number are experiencing remission as a result of either incomplete cancer cell elimination or harmful treatment side effects. The immune response is usually capable of identifying, controlling, and eliminating the cancer cells in the same manner used by the immune system for viral infection, as most of the cancer cells are byproducts of the viral infection. Nk cells and CD8+ cytotoxic T cells play a major role in the elimination of cancer cells (19). A healthy cell needs to go through malignant transformation to become a cancerous cell, which means it needs to accumulate multiple mutations and, in several genes, maintain cell division, proliferation, and survival. Tumor oncogenes encode the protein that prevents the proliferation of mutant cells. p53 is a well-known tumor oncogene that regulates apoptosis in cells with damaged DNA. Loss of p53 or substitution of p53 is the vital reason for compromising the protective function of this protein. In this review paper, we are stressing the contribution of lncRNAs as a target for cancer immunotherapy (239). The p53 protein regulates the lincRNA p-21 locus, which is responsible for gene expression both at the transcriptional and posttranscriptional levels and is associated with the RNA-binding protein HuR. LncRNA also has a role in cell apoptosis, with PANDA being a monoaxenic lncRNA. Targeting lncRNA can be a useful tool for encountering PARP-associated drug resistance, p53 reactivation, and finally tumor suppression. Oncogene suppressor lncRNA is also deemed to inhibit the PD-1/PD-L1 pathway, which is associated with immune invasion of cancer cells, to provide antitumor immunity. Targeting lncRNA could also be associated with regulating other molecular pathways such as STAT, ZEB, and P13k/Akt (19). LncRNA can be targeted in cancer immunotherapy with regard to its specificity in directing the immune response toward the targeted MDC population and function in cancer. LncRNA expression is an important regulator of several biological processes; hence, it is targeted for modulating complications other than cancer (240). TAMs have two classes: proinflammatory, antitumorigenic M1 and anti-inflammatory, pro-tumorigenic M2. LncRNA promotes M2 polarization by regulating TCF-4 and GNAS-AS1/MIR4319/NECAB3. GNAS-AS1 and XIST lncRNAs have a key role in M2 macrophage polarization in NSCLC and clinical tumor cells. In osteosarcoma, tumor progression and proliferation are associated with Lifr-AS1 lncRNA. In contrast, COX2 lncRNA inhibits M2 polarization and thus antagonizes HCC immune escape and tumor growth. In endometrial cancer, M2-macrophage is inhibited by NIFK-AS1 targeting miR-146a. In a nutshell, lncRNA is contributing as a potential biological target for cancer therapy (241). Aptamers are single-stranded oligonucleotides that can bind to a diverse range of molecular targets with high affinity and specificity (239). Designing aptamers for specific upregulated lncRNA is one of the most common strategies for lncRNA-based targeted therapy (242). For instance, knocking out NEAT1 and MALAT1 from the promoter region inhibits cancer metastasis. Aptamers can be used with mAbs or alone, which are capable of inhibiting pro-metastatic miR-214 and simultaneously upregulating anti-metastatic miR-148b, thus inhibiting metastasis (243). LncRNAs play a crucial role in a immunosuppressive TIME. A complex network of fibroblasts, endothelial cells, and adaptive and innate immune cells supports tumor cells by secreting cytokines, chemokines, growth factors, and metabolites that promote tumor growth and proliferation. Cancer-associated fibroblasts (CAFs) induce angiogenesis by upregulating CXCL12 lncRNA and downregulating tumor-suppressor miR-101. Regulatory T cells are CD4+ lymphocytes characterized by the expression of Foxp-3. Flatr lncRNA is the key regulator of Foxp-3, which is responsible for secreting chemokines (CCL1, CCL22, and CCL17), cytokines like IL-10, growth factors TGF-β, and co-inhibitory receptors PD-1 and CTLA-4. To conclude, lncRNA expression is a major player in poor prognosis and malignancies; hence, it could be a novel tool in cancer immunotherapy with high affinity and specificity toward the respective cancer-producing cells (239).

## 6 Discussion

LncRNAs have been identified as multifunctional regulators that play crucial roles in development, immunological regulation, cellular physiology, and the pathogenesis of various diseases, including cancer (20). Their potential as useful therapeutic targets in the therapy of cancer has been demonstrated by their diverse roles. However, unlike their RNA siblings, mRNA and miRNA, it is clear that the therapeutic targeting of lncRNAs, as well as the effective and safe delivery of therapeutic drugs, have been much neglected. A noteworthy example of the significant gaps that still exist in this developing discipline is the small number of clinical studies employing lncRNAs (20, 244). Numerous studies have previously demonstrated that many lncRNAs can function as oncogenes, and numerous others serve as tumor suppressors. Regarding protein tumor suppressors and oncogenes, this offers new opportunities for targeted cancer treatments by reinstating and boosting lost lncRNA tumor-suppressor behavior and blocking lncRNA oncogenic activity that promotes tumor development along with treatment resistance (244). LncRNAs have been found to be connected with resistance to cancer treatment advancement and oncogenesis. For instance, it has been demonstrated that the GAS5 lncRNA controls cell longevity and development, exhibits tumor suppressor function in a variety of malignancies, and regains sensitivity to chemotherapy medications. Numerous studies, like this one, highlight how oligonucleotide-based treatment may induce extremely targeted control of lncRNA expression, which has obvious applications in cancer therapy (122, 244). Nevertheless, lncRNA-based therapeutics have drawbacks, including how they transmit certain compounds to target cells and safety issues during treatment. Using lncRNA as a therapeutic target is not without its difficulties, such as issues with molecular transport and inappropriate consequences (20). To guarantee that therapeutic drugs are transported precisely and effectively to the targeted lncRNAs, novel delivery mechanisms must be created. Reaching this degree of accuracy is essential to maximizing the effects of therapy (245).

LncRNAs must also be functionally evaluated and validated *in vivo* in order to be used in therapeutic contexts. Numerous methods have been devised to verify the functional links between lncRNAs using experimental techniques (246–248). These strategies comprise techniques like capture hybridization analysis of RNA targets (CHART) for lncRNA target gene identification, chromatin isolation by RNA purification-sequencing (ChIRP), and RNA antisense purification (RAP) (246). Furthermore, an enormous increase in the quantity of lncRNAs annotated in the human genome has been made possible by advances in transcriptomics; however, only a minor portion of these have been functionally characterized (247). LncRNA expression necessitates the examination of intricate connections between lncRNA molecules and their corresponding target genes and proteins in model organisms. Nevertheless, the conservation of lncRNAs across different species is limited, posing a challenge to the advancement of therapeutic approaches aimed at addressing lncRNAs. Hence, it is imperative to thoroughly investigate the barriers hindering the progress of lncRNA-targeting therapeutics by means of the utilization of bioinformatics, extensive databases, and high-throughput advances in technology. This approach will facilitate the acquisition of a profound comprehension of lncRNA localization, structures, functional motifs, mechanisms of action, and their intricate associations with other molecules in biology (248).

Innovative therapeutic approaches aim to manipulate lncRNAs, either by knocking down oncogenic lncRNAs or by restoring tumor-suppressor lncRNAs. Techniques like RNAi, ASOs, and CRISPR/Cas9-mediated gene editing are employed to modulate the function of lncRNAs specifically. These strategies hold the potential to halt cancer progression, enhance the efficacy of existing treatments, and reduce unwanted side effects by targeting the molecular peculiarities of cancer cells directly (249).

Despite the potential of lncRNA-targeted therapies, the secure and effective delivery of these therapeutic agents poses a significant challenge. Nucleic acid-based drugs, including those targeting lncRNAs, are inherently unstable in the physiological environment and are susceptible to rapid degradation by nucleases. Moreover, they need to overcome various biological barriers to reach their target cells effectively. Researchers are developing advanced delivery systems like lipid nanoparticles (LNPs) and polymer-based carriers to address these issues. These systems protect the therapeutic molecules from enzymatic degradation, improve their half-life in the bloodstream, and facilitate targeted delivery to tumor cells. The development of such delivery vehicles is crucial for the clinical success of lncRNA-targeting therapies, as they ensure that the therapeutic agents are delivered efficiently to the site of action while minimizing off-target effects and toxicity. The targeted delivery of lncRNA therapies might be improved by making nanoparticles better and looking into other new ways to deliver drugs, like exosome-based systems. To make cancer treatments even better, combining lncRNA-targeting strategies with other methods like immunotherapy and chemotherapy could create more complete and effective plans (250). **Table 5** outlines the difficulties encountered in immunotherapy directed toward lncRNA.

**Table 5:** Overcoming challenges in developing lncRNA-targeted immunotherapies.

Challenge category	Specific issue	Impact on therapy	Current solutions	Limitations of current solutions	Research gaps	Future directions	References
Delivery	Stability in bloodstream	Reduced therapeutic efficacy due to degradation	Lipid nanoparticles, polymeric nanoparticles	Limited stability, potential for off-target effects	Develop more biocompatible and stable delivery systems	Biodegradable nanoparticles, targeted delivery systems	(251)
Delivery	Intracellular delivery to target cells	Inefficient delivery to specific cell types	Viral vectors, cell-penetrating peptides	Limited specificity, potential for insertional mutagenesis	Develop cell-specific targeting strategies	Nanotechnology-based delivery systems, cellular barcoding techniques	(252)
Off-target effects	Lack of specificity for target lncRNA	Increased toxicity and unintended immune responses	CRISPR-Cas9, antisense oligonucleotides	Limited efficiency, potential for off-target edits	Develop high-fidelity targeting tools, personalized medicine approaches	Base editing technologies, single-cell RNA sequencing for target identification	(253)
Off-target effects	Difficulty distinguishing isoforms of lncRNA	Reduced efficacy and potential for off-targeting	Functional assays, allele-specific targeting	Limited sensitivity and specificity	Develop isoform-specific targeting strategies, better functional characterization of lncRNAs	Machine learning-based target identification, single-molecule imaging techniques	(254)
Immune tolerance	Tumor-induced immunosuppression	Reduced efficacy of lncRNA-targeted immune response	Checkpoint inhibitors, combination therapies	Limited efficacy in certain tumor types, potential for immune-related adverse events	Develop strategies to overcome immunosuppressive microenvironment, personalized immune profiling	CAR-T cell therapy, bispecific antibodies	(255)
Immune tolerance	Regulatory T-cell (Treg) suppression	Treg-mediated suppression of antitumor immune response	Treg depletion, immune checkpoint blockade	Potential for autoimmune side effects, lack of long-term efficacy	Develop strategies to selectively target Tregs in the TIME	Treg-specific CAR-T cells, bispecific T-cell engagers	(245)
Clinical trial design	Defining optimal dose and treatment schedule	Lack of standardized protocols	Phase I/II clinical trials, biomarker development	Limited understanding of lncRNA dynamics, inter-patient variability	Develop personalized dosing strategies based on lncRNA expression and immune response	Liquid biopsies, pharmacodynamic modeling	(256)
Safety and regulatory concerns	Long-term safety profile of lncRNA-targeted therapies	Lack of established safety guidelines	Preclinical safety studies, early-phase clinical trials	Limited understanding of potential off-target effects, difficulty monitoring long-term safety	Develop comprehensive safety assessment protocols, personalized risk stratification	Advanced imaging techniques, long-term follow-up studies	(20)
Cost and accessibility	High cost of development and manufacturing	Limited access to lncRNA-targeted therapies for all patients	Streamlined manufacturing processes, cost-effective delivery systems	Lack of reimbursement pathways, inequities in healthcare access	Develop cost-effective production methods, advocate for equitable access to novel therapies	Public–private partnerships, innovative financing models	(251)

## 7 Concluding remarks

Emerging evidence suggests that lncRNAs play an important regulatory role in the immune system and contribute to resistance to cancer immunotherapies. Therefore, lncRNAs are promising cancer immunotherapeutic targets. Many lncRNAs are aberrantly expressed in cancer, and they also affect angiogenesis, metastasis, apoptosis, and tumor growth (257). Recent research has identified immune-related lncRNAs as gene expression regulators specific to immune cells, which mediate immune stimulatory and immunological response repression. This suggests that targeting lncRNAs could enhance the effectiveness of immunotherapy (250). However, there have been a limited number of documented clinical trials using lncRNAs, and little attention has been paid to targeting lncRNAs therapeutically in cancer as well as the effective and secure delivery of the reagents (256).
