# Supplementary material for: Exploring potential roles of long non-coding RNAs in cancer immunotherapy: a comprehensive review
Source: Front Immunol. 2024 Aug 27;15:1446937. doi: 10.3389/fimmu.2024.1446937 (PMC11384988; doi:10.3389/fimmu.2024.1446937)
Supplement: Supplementary file 1 [file DataSheet1.pdf]

Supplementary Material

Exploring Potential Roles of Long Non-Coding RNAs in Cancer Immunotherapy: A Comprehensive Review

Asghar Arshi[1,5], Esmaeil Mahmoudi[2,5], Farzaneh Raeisi[1], Masoud Dehghan Tezerjani[3], Elham Bahramian[4], Yeasin Ahmed[4], Chun Peng[1*]

1. Department of Biology, York University, Toronto, ON, M3J 1P3, Canada.

2. Young Researchers and Elite Club, Shahrekord Branch, Islamic Azad University, Shahrekord, Iran.

3. Department of bioinformatics, School of Advanced Medical Technologies, Isfahan University of Medical Sciences, Isfahan, Iran.

4. Department of Biological Sciences, University of Arkansas, Fayetteville, AR, 72701, USA.

5. These authors contributed equally to this work.

Corresponding author

Dr. Chun Peng, Full Professor,

Department of Biology,

York University,

Toronto, ON, M3J 1P3.

Email: cpeng@yorku.ca

Tell: (416)736-2100

Supplementary Table 1: Types of adoptive cell therapies: comparing CAR-T cells, TCR-engineered T-cells, and TILs.

Type of Therapy	Target Diseases	Mechanism of Action	Key Examples	Clinical Status	Efficacy and Outcomes	References
CAR-T cells	Hematological malignancies (acute lymphoblastic leukemia, lymphoma)	Engineered T-cells with chimeric antigen receptors (CARs) recognizing specific cancer antigens	Kymriah (tisagenlecleucel), Yescarta (axicabtagene ciloleucel)	FDA-approved for specific indications	High response rates in some cancers, improved survival, but potential for relapse	(1)
TCR-engineered T-cells	Solid tumors (melanoma, synovial sarcoma, mesothelioma)	Engineered T-cells with T cell receptors (TCRs) recognizing specific tumor antigens	No commercially available products, several investigational therapies in clinical trials	Early clinical trials, promising results in some patients, but limited data on efficacy	Potential for CRS, neurotoxicity, graft-versus-host disease (GVHD)	(2)
Tumor-infiltrating lymphocytes (TILs)	Solid tumors (melanoma, head and neck cancer, sarcoma)	Expansion of tumor-specific T-cells isolated from patient's tumor	No commercially available products, individual patient-specific therapies in clinical trials	Early clinical trials, encouraging results in some patients, but complex manufacturing process	Limited data on efficacy, potential for CRS, GVHD, and other immune-related adverse events	(3)
CAR-NK cells	Hematological malignancies and solid tumors	Engineered natural killer (NK) cells with CARs	Investigational therapies in early clinical trials	Preclinical data suggests potential efficacy, safety profile still under investigation	Potential for CRS, off-target toxicities	(4)
TCR-gamma delta T-cells	Solid tumors and hematological malignancies	Engineered gamma delta T-cells with TCRs recognizing tumor antigens	Investigational therapies in early clinical trials	Preclinical data suggests potential for wider antigen recognition, safety profile still under investigation	Potential for CRS, GVHD	(5)
CAR-Macrophages	Solid tumors	Engineered macrophages with CARs recognizing tumor antigens	Preclinical research stage	Early research suggests potential for tumor targeting and immune activation, safety profile unknown	Potential for off-target toxicities, immune suppression	(6)
CAR-Dendritic cells	Solid tumors	Engineered dendritic cells with CARs to activate T cell responses	Preclinical research stage	Early research suggests potential for antigen presentation and T cell priming, safety profile unknown	Potential for off-target toxicities, autoimmune reactions	(7)

TCR-engineered regulatory T-cells (Tregs)	Autoimmune diseases and inflammatory conditions	Engineered Tregs with TCRs recognizing specific antigens to suppress immune response	Preclinical research stage	Early research suggests potential for targeted immune suppression, safety profile unknown	Potential for off-target immune suppression, loss of immune control	(8)
TCR gene editing	Various diseases, including cancer and genetic disorders	Editing genes within T-cells to create new TCRs recognizing specific antigens	Preclinical research stage	Early research suggests potential for personalized therapy, safety profile and long-term effects unknown	Potential for off-target effects, genotoxicity	(9)
Universal off-the-shelf cell therapies	Various diseases	Developing standardized, allogeneic T cell or NK cell products for broader patient access	Preclinical research stage	Early research suggests potential for wider accessibility and reduced manufacturing time, safety profile and long-term effects unknown	Potential for GVHD, rejection, and limited efficacy	(10)

Supplementary Table 2: Cancer vaccines: differentiating between preventive and therapeutic vaccines.

Vaccine Type	Vaccine Name	Target Cancer	Mechanism of Action	Key Findings/Efficacy	References
Preventive	Gardasil	Cervical, vulvar, vaginal, anal, and head and neck cancers	Triggers immune response against HPV types 16 and 18	Reduces risk of HPV-related cancers by up to 90%	(11)
Preventive	Cervarix	Cervical cancer	Triggers immune response against HPV types 16 and 18	Reduces risk of cervical cancer by up to 80%	(12)
Therapeutic	Sipuleucel-T (Provenge)	Prostate cancer	Uses modified patient's own immune cells to attack cancer cells	Improves overall survival in advanced prostate cancer by a few months	(13)
Therapeutic	Pembrolizumab (Keytruda)	Various cancers (melanoma, lung, bladder, etc.)	Blocks immune checkpoint to activate T-cells against cancer	Significant tumor shrinkage and improved survival in various cancers	(14)
Therapeutic	CAR T-cell therapy (e.g., Kymriah)	Leukemia and lymphoma	Genetically modifies T-cells to recognize and attack specific cancer cells	High remission rates in some blood cancers	(15)
Therapeutic	mRNA cancer vaccines (e.g., Moderna mRNA-4157)	Various cancers	Delivers tumor-specific mRNA to instruct immune cells to attack cancer	Early promising results in inducing immune response and tumor regression	(16)
Therapeutic	Dendritic cell vaccines (e.g., DCVax-L)	Various cancers	Uses patient's own dendritic cells loaded with tumor antigens to stimulate immune response	Mixed results, with some trials showing potential benefit	(17)
Preventive	Hepatitis B vaccine	Liver cancer	Prevents hepatitis B infection, reducing risk of liver cancer	Reduces liver cancer risk by over 70%	(18)
Preventive	Human papillomavirus (HPV) vaccines (Gardasil 9, Cervarix)	Cervical, vulvar, vaginal, anal, and head and neck cancers	Triggers immune response against multiple HPV types (including 16, 18, 31, 33, 45, 52, and 58)	Further reduces risk of HPV-related cancers compared to Gardasil/Cervarix	(19)
Therapeutic	Oncolytic virus therapy (e.g., T-VEC)	Various cancers	Uses genetically modified viruses to infect and kill cancer cells	Shows promising results in treating some cancers, but further research needed	(20)

Supplementary Table 3: Dysregulated expression of lncRNAs and their potential clinical implications in different cancers.

Cancer Type	lncRNA Name	Expression Pattern	Targeted Gene(s)	Functional Role	Ref.
**Breast**	TTN-AS1	Upregulated	DGCR8	Promotes migration and invasion	(21)
	LINC01787	Upregulated	pre-miR-125b	Promotes proliferation and migration	(22)
	LINC00899	Upregulated	miR-425	Inhibits proliferation, migration, and invasion	(23)
	NBAT1	Upregulated	DKK1/EZH2/PRC2	Promotes migration and invasion	(24)
	OTAIR	Upregulated	PRC2	Promotes invasiveness and metastasis	(25)
	ARNILA	Upregulated	Sox4	Promotes EMT, invasion and metastasis	(26)
	LINC00628	Upregulated	Bcl-2/Bax	Inhibits proliferation, invasion, and migration/Promotes apoptosis	(27)
	XIST	Upregulated	miR-155	Inhibits growth, migration, and invasion	(28)
	HOST2	Upregulated	let-7b	Promotes cell motility, migration, and invasion	(29)
	CASC2	Upregulated	miR-96-5p/SYVN1	Inhibits viability, migration and invasion/Promotes apoptosis	(30)
	LINC00115	Upregulated	miR-7	Promotes metastasis	(31)
	H19	Downregulated	miR-138	Inhibits proliferation, invasion, and migration	(32)
	Linc-ITGB1	Downregulated	N-cadherin/vimentin	Inhibits EMT, invasion and migration	(33)
	LINC01296	Downregulated	Bcl-2	Inhibits cell proliferation, metastasis	(34)
	SNHG14	Downregulated	miR-193a-3p	Inhibits invasion	(35)
	GHET1	Downregulated	N-cadherin/Vimentin	Inhibits cell proliferation, invasion, and migration	(36)
	LINC00673	Downregulated	B7-H6	Inhibits proliferation and metastasis	(37)
**Lung**	MALAT1	Upregulated	SR/PC2/hnRNP C	Induces proliferation and invasion	(38)
	HOTAIR	Upregulated	PRC2/LSD1	Induces proliferation and invasion	(39)
	H19	Upregulated	c-Myc/p53/miR-675	Induces NSCLC cell proliferation	(40)
	PVT1	Upregulated	miR-200a/miR-200b/MMP9	Induces proliferation	(41)
	ANRIL	Upregulated	P15/P21/KLF2	Induces proliferation and invasion	(42)
	LINC00473	Upregulated	CRTC/CREB	Induces growth of LKB1-inactivated NSCLC cells	(43)
	LINC00963	Upregulated	PGK1/CRTC/CREB	Promotes metastasis	(44)
	SOX2OT	Upregulated	EZH2	Promotes cell proliferation	(45)
	CAR10	Upregulated	YB-1/miR-203/30/SNAI	Induces proliferation	(46)
	BANCR	Downregulated	p38	Induces proliferation and migration	(47)
	PANDAR	Downregulated	PANDAR/NF-YA/Bcl-2	Induces apoptosis	(48)
	MIR22HG	Downregulated	YBX1/MET/p21	Suppresses proliferation and invasion	(49)
	MEG3	Downregulated	p53	Cisplatin sensitivity	(50)
	GAS5	Downregulated	P53, E2F1, miR-21	EGFR-TKIs sensitivity	(51)

**Gastric**	MALAT1	Upregulated	miR-23B-3P/ATG12/miR-30b/ATG5/SOX2	Induces autophagy	(52)
	AK022798	Upregulated	MRP1	Inhibits cell apoptosis	(53)
	CRAL	Upregulated	miR-505/CYLD	Promotes DNA damage and apoptosis	(54)
	ROR	Upregulated	MRP1	Inhibits cell apoptosis	(55)
	XLOC_006753	Upregulated	Vimentin/Snail	Inhibits cell apoptosis/Promots EMT	(56)
	MACC1-AS1	Upregulated	miR-145-5p/CD133/OCT4/SOX2/LIN28	Increases cancer stemness	(57)
	D63785	Upregulated	miR-422a/MEF2D	Inhibits cell apoptosis	(58)
	LINC01433	Upregulated	YAP/USP9X	Inhibits cell apoptosis	(59)
	HOXD-AS1	Upregulated	EZH2/PDCD4	Epigenetically silencing PDCD4 via recruiting EZH2	(60)
	HULC	Upregulated	FOXM1	Suppresses autophagy/Promotes cell apoptosis	(61)
	PCAT-1	Upregulated	miR-128/ZEB1/EZH2	Promotes EMT	(60)
	HOTAIR	Upregulated	miR-17-5p/PTEN/miR-217/miR-34a/miR-126	Promotes EMT and cell proliferation/Inhibitis cell apoptosis	(62)
	THOR	Upregulated	SOX9	Increases cancer stemness	(63)
	BLACAT1	Upregulated	miR-361/ABCB1	Inhibits apoptosis/Promotes invasion	(64)
	GHET1	Upregulated	BAK/BCL-2/MDR1/MRP1	Inhibits cell apoptosis	(65)
	ANRIL	Upregulated	MDR1/MRP1	Regulates MDR-related genes	(66)
	UCA1	Upregulated	PARP/BCL-2/miR-27b	Inhibits cell apoptosis	(67)
	PVT1	Upregulated	BCL-2/MDR1/MRP1	Inhibits cell apoptosis	(68)
	MRUL	Upregulated	ABCB1	Inhibits cell apoptosis	(69)
	SNHG5	Upregulated	BCL-2/BAX/MDR1/MRP1	Inhibits cell apoptosis	(70)
	DANCR	Upregulated	MDR1/MRP1	Inhibits cell apoptosis	(71)
	FAM84B-AS	Upregulated	FAM84B/BCL2/BCL-xL	Inhibits cell apoptosis	(72)
	BCAR4	Upregulated	OCT3/4/SOX2/c-Myc/KLF4	Increases cancer stemness	(70)
	NEAT1	Upregulated	N/A	Inhibits cell apoptosis/Promotes invasion	(73)
	CASC9	Upregulated	MDR1	Inhibits cell apoptosis/Promotes cell proliferation	(74)
	HOTTIP	Upregulated	Vimentin/ZEB1/Twist	Promotes EMT	(75)
	HCP5	Upregulated	MiR-3619-5p/SOX2/OCT4/LIN28/CD1331	Increases cancer stemness	(76)
	CASC2	Downregulated	miR-19a	Promotes cell apoptosis	(77)
	LEIGC	Downregulated	Vimentin/Twist/Slug/ZEB1/Snail	Inhibits EMT	(78)
**Thyroid**	H19	Upregulated	miR-17-5p/YES1	Promotes proliferation, migration, and invasion	(79)
	HCP5	Upregulated	miR-22-3p/miR-186-5p/miR-216a-5p/ST6GAL2	Promotes proliferation, migration, invasiveness	(80)
	HOTTIP	Upregulated	miR-637	Promotes proliferation, invasion, and migration	(81)
	MALAT1	Upregulated	miR-21	Inhibits cell proliferation and invasion	(82)
	SNHG15	Upregulated	miR-200a-3p	Promotes cell growth and migration	(83)

	SPRY4-IT	Upregulated	TGF-β1/Smad	Promotes proliferation	(84)
	OIP5-AS1	Upregulated	FXR1/YY1/CTNNB1	Promotes cell proliferation and migration	(85)
	Linc00210	Upregulated	miR-195-5p/IGF1R/Akt	Promotes proliferation, migration, and invasion	(86)
	UCA1	Upregulated	UCA1/miR-15a	Promotes cell proliferation	(87)
	CCAT1	Upregulated	miR-143	Promotes cell proliferation, migration, and invasion	(88)
	XIST	Upregulated	miR-34a	Promotes cell proliferation and tumor growth	(89)
	NEAT1	Upregulated	miRNA-214	Promotes cell growth and metastasis	(90)
	NR2F1-AS1	Upregulated	miR-423-5p/SOX12	Promotes proliferation and invasion	(91)
	LINC00152	Upregulated	miR-497/BDNF	Promotes cell growth and invasion	(92)
	MCM3AP-AS1	Upregulated	MCM3AP-AS1/miR-211-5p/ SPARC	Promotes proliferation, migration, and invasion	(93)
	AB074169	Downregulated	CDKN1a	Inhibits cell growth	(94)
	ASMTL-AS1	Downregulated	miR-93-3p/miR-660/FOXO1	Inhibits cell proliferation and glycolysis	(95)
	GAS8-AS1	Downregulated	ATG5	Inhibits proliferation	(96)
	MEG3	Downregulated	miR-182	Inhibits iodine-resistant cell viability/Promotes apoptosis and DNA damage	(97)
	PAR5	Downregulated	EZH2	Inhibits proliferation and migration	(98)
	HOTAIRM1	Downregulated	TDG	Inhibits cell proliferation, invasion, and migration	(99)
	PAPAS	Downregulated	HOTTIP	Inhibits cell proliferation	(100)
**Brain**	HOXA11-AS	Upregulated	Tpl2-MEK1/2-ERK1/2	Sensitizes glioblastoma cells to ROS, drastically impairing tumor growth and prolonging survival of mouses	(101)
	LINC01018	Upregulated	KNG1	Suppresses the migration, invasion, and proliferation of glioma cells	(102)
	LINC01018	Upregulated	ADRA2C/ RAB6B/RAB27B/ RAPGEF5/STEAP2/TAGLN3/UNC13C	Inhibits cell proliferation and metastasis	(103)
	TUSC7	Upregulated	BDNF/ERK	Suppresses glioma cell proliferation and migration	(104)
	DANCR	Upregulated	DLX6/ATG7	Promotes intracellular autophagy and proliferation and reduce apoptosis	(105)
	LINC01426	Downregulated	Mdm2	Inhibits proliferation and induces apoptosis	(106)
	MIR210HG	Downregulated	LMX1A	Suppresses glioma cell proliferation	(107)
	PDCD4-AS1	Downregulated	METTL7B	Inhibits glioma cell proliferation, invasion, migration, and induces cell cycle arrest	(108)
	GSCAR	Downregulated	SRSF1	Promotes tumor cell responses to TMZ	(109)
	SPRY4-IT1	Downregulated	EZH2/VEGFA	Achieves cell proliferation and angiogenesis	(110)

**Liver**	ATB	Upregulated	miR-200	Promotes metastasis	(111)
	UFC1	Upregulated	β-catenin/HuR	Promotes proliferation	(112)
	PXN-AS1	Upregulated	PXN	Promotes proliferation	(113)
	ANRIL	Upregulated	EZH2	Promotes proliferation	(114)
	TUG1	Upregulated	EZH2	Promotes proliferation and glycolysis	(115)
	lnc-β-Catm	Upregulated	EZH2/β-catenin	Promotes stemness	(116)
	GIHCG	Upregulated	EZH2	Promotes proliferation and metastasis	(117)
	DANCR	Upregulated	β-catenin	Promotes stemness	(118)
	MALAT1	Upregulated	miR-143-3p	Promotes metastasis and proliferation	(119)
	HULC	Upregulated	miR-186/miR-9	Promotes proliferation and lipogenesis	(120)
	XIST	Upregulated	miR-92b	Inhibits metastasis and proliferation	(121)
	PVT1	Upregulated	NOP2	Promotes stemness	(122)
	HOTTIP	Upregulated	miR-192/miR-204	Promotes glutaminolysis	(123)
	DILC	Upregulated	IL-6	Inhibits stemness	(124)
	ICR	Upregulated	ICAM-1	Promotes stemness	(125)
	ZFAS1	Upregulated	miR-150	Promotes metastasis	(126)
	MVIH	Upregulated	PGK1	Promotes metastasis	(127)
	CASC9	Upregulated	HNRNPL	Promotes proliferation	(128)
	Dreh	Downregulated	Vimentin	Inhibits metastasis and proliferation	(129)
**Kidney**	HOTAIR	Upregulated	EZH2/miR-141/Ago2	Promotes proliferation, invasion, cell cycle	(130)
	lncRNA-ATB	Upregulated	EMT	Promotes proliferation, apoptosis, migration, invasion	(131)
	MALAT1	Upregulated	Ezh2/miR-205	Promotes proliferation, migration, invasion	(132)
	HEIRCC	Upregulated	EMT	Promotes proliferation, apoptosis, migration, invasion	(133)
	PVT1	Upregulated	MYC	Promotes promoter hypomethylation	(134)
	CADM1-AS1	Downregulated	CADM1	Promotes cell proliferation, apoptosis and migration	(135)
	MEG3	Downregulated	Bcl-2/rocaspase-9/leaved caspase-9/cytochrome c	Promotes apoptosis, mitochondrial pathway	(136)
	BX357664	Downregulated	EMT/MMP2/MMP9/TGF-β1/p38/HSP27	Promotes proliferation, migration, invasion, cell cycle	(137)
	CASC2	Downregulated	miR-21	Promotes proliferation, migration	(138)
**Colorectal**	ATB	Upregulated	E-cadherin/ZO-1/ZEB1/N-cadherin	Promotes invasion, induces EMT	(139)
	BC200	Upregulated	STAT3/β-catenin	Promotes proliferation, invasion, EMT/Inhibits apoptosis, regulates cell cycle	(140)
	CASC15	Upregulated	miR-4310/LGR5	Promotes proliferation, migration, invasion	(141)
	CCAT1	Upregulated	c-Myc	Promotes proliferation, invasion, drug resistance	(142)

	CCAT2	Upregulated	miR-145	Promotes growth and metastasis	(143)
	CYTOR	Upregulated	β-catenin/TCF complex	Promotes migration, invasion, EMT	(144)
	DMTF1v4	Upregulated	p-ERK/p-JNK/p-p38	Increases proliferation, migration/Inhibits apoptosis	(145)
	ENST00000455974	Upregulated	JAG2	Promotes proliferation and migration	(146)
	FAL1	Upregulated	STAT3/TGF-β1/Bcl-2/p65/PCNA	Promotes proliferation, invasion/Inhibits apoptosis	(147)
	FAM83H-AS1	Upregulated	TGF-β	Promotes tumorigenesis	(148)
	GSEC	Upregulated	DHX36	Promotes migration	(149)
	HNF1A-AS1	Upregulated	miR-34a/p53	Promotes proliferation, migration, invasion	(150)
	HOTAIR	Upregulated	E-cadherin/vimentin/MMP-9	Promotes migration, invasion	(151)
	HULC	Upregulated	miR-613/RTKN/vimentin/N-cadherin/E-cadherin	Promotes proliferation, migration, invasion	(152)
	H19	Upregulated	miR138/HMGA1/miR-675-5p	Promotes invasion, migration, drug resistance	(153)
	Linc01106	Upregulated	miR-449b-5p/Gli	Promotes proliferation, migration, stemness	(154)
	Linc01234	Upregulated	miR-642a-5p/SHMT2	Promotes proliferation	(155)
	Linc01567	Upregulated	miR-93	Promotes proliferation, invasion, migration	(156)
	Linc01578	Upregulated	NF-κB/YY1	Promotes metastasis	(157)
	Linc02418	Upregulated	miR-34b-5p/Bcl-2	Promotes growth, mobility, invasion/Inhibits apoptosis	(158)
	Lnc34a	Upregulated	Dnmt3a/PHB2/HDAC1	Promotes proliferation, stem cells	(159)
	Loc441461	Upregulated	PhoA/ROCK	Promotes growth, motility/Inhibits apoptosis	(160)
	LincDUSP	Upregulated	ATR/p53/E2F/c-Myc	Promotes proliferation, stem cells, modulates DNA damage response and cell cycle/Inhibits apoptosis	(161)
	MALAT1	Upregulated	miR-129-5p/HMGB1/miR-663a	Promotes proliferation, invasion, migration	(162)
	MYU	Upregulated	hnRNP-K/CDK6	Promotes proliferation, controls cell cycle	(163)
	PCAT6	Upregulated	EZH2	Promotes proliferation and apoptosis	(164)
	PVT1	Upregulated	miR-26b/miR-30d-5p/RUNX2	Promotes proliferation, migration, invasion	(165)
	ROR	Upregulated	miR-145	Promotes proliferation, migration, invasion	(166)
	RP11-619 L19.2	Upregulated	miR-1271-5p/CD164	Promotes proliferation, migration, invasion, EMT	(167)
	SBDSP1	Upregulated	p21/cyclin D1	Promotes proliferation, migration, invasion, regulates cell cycle	(168)
	SNHG1	Upregulated	c-Myc/cyclin D	Promotes proliferation, invasion, migration/Inhibits apoptosis	(169)
	SNHG7	Upregulated	miR-193b/K-ras/ERK/cyclinD1	Promotes proliferation/Inhibits apoptosis	(170)
	SNHG15	Upregulated	Slug	Promotes proliferation and migration	(171)
	SNHG17	Upregulated	miR-375/CBX3	Promotes proliferation, migration, invasion	(172)

	TUG1	Upregulated	miR-26a-5p/MMP-14/VEGF/MAPK/Hsp27	Promotes proliferation, migration, invasion, EMT/Inhibits apoptosis,	(173)
	UPAT	Upregulated	UHRF1	Promotes survival	(174)
	USP2-AS1	Upregulated	Phosph-YAP	Promotes proliferation and metastasis	(175)
	XIST	Upregulated	miR-34a/β-catenin cyclin D1/c-Myc/MMP-7	Promotes proliferation	(176)
	ZEB1-AS1	Upregulated	miR-455-3p/PAK2	Promotes proliferation, migration, invasion	(177)
	ZFAS1	Upregulated	ZEB1/E-cadherin/ZO-1/vimentin/N-cadherin	Promotes proliferation, invasion, EMT/Inhibits apoptosis,	(178)
	Pint	Downregulated	PRC2	Inhibits proliferation	(179)
	Loc285194	Downregulated	miR-211	Inhibits proliferation	(180)
	Linc00261	Downregulated	β-catenin	Inhibits proliferation, invasion, migration, drug resistance/Promotes apoptosis	(181)
	Linc00657	Downregulated	CAPN7	Inhibits proliferation, invasion/Promotes apoptosis	(182)
	DACOR1	Downregulated	Cystathionine β-synthase	Inhibits proliferation/Promotes DNA methylation	(183)
	B3GALT5-AS1	Downregulated	miR-203/ZEB2/Snail	Inhibits proliferation/Promotes migration, invasion, EMT	(184)
**Prostate**	CASC11	Upregulated	YBX1/miR-145	Promotes proliferation/Inhibits miR-145	(185)
	SNHG17	Upregulated	TCF1/TCF4/LEF1/c-myc/cyclin D1/axin2	Promotes proliferation/Inhibits apoptosis	(186)
	SNHG16	Upregulated	miR-373-3p	Promotes proliferation and migration	(187)
	SNHG14	Upregulated	miR-5590-3p/YY1/Cyclin D1/Bcl-2/N cadherin/Bax/Caspase-3/E cadherin	Promotes proliferation and invasion	(188)
	SNHG12	Upregulated	CCNE1/miR-195	Promotes apoptosis and autophagy	(189)
	SNHG11	Upregulated	miR-184	Promotes progression	(190)
	SNHG8	Upregulated	miR-384/HOXB7	Promotes proliferation, migration and invasion	(191)
	SNHG7	Upregulated	c-Myc	Inhibits proliferation and glycolysis	(192)
	SNHG3	Upregulated	miR-152-3p/miR-214-3p	Promotes proliferation, invasion, and migration/Inhibits metastasis	(193)
	SNHG1	Upregulated	EZH2	Promotes proliferation, apoptosis, migration, invasion	(194)
	lncHUPC1	Upregulated	FOXA1/SDCCAG3/miR-133b	Promotes metastasis and growth	(195)

	MNX1-AS1	Upregulated	miR-2113	Promotes proliferation, migration and invasion	(196)
	CERS6-AS1	Upregulated	miR-16-5p	Inhibits proliferation and migration	(197)
	DANCR	Upregulated	miR-33b-5p	Promotes proliferation, migration, and taxol resistance	(198)
	MALAT1	Upregulated	MYBL2	Inhibits p-mTOR	(199)
	PCGEM1	Upregulated	miR-129-5p	Promotes progression	(200)
	NEAT1	Upregulated	LDHA/miR-766-5p	Promotes progression	(201)
	DLEU2	Upregulated	miR-582-5p	Promotes proliferation invasion, and migration	(202)
	EIF3J-AS1	Upregulated	MAFG	Promotes progression	(203)
	HOXA-AS2	Upregulated	miR-509-3p/PBX3	Inhibits proliferation and migration	(204)
	LncAY927529	Upregulated	CXCL14	Promotes proliferation and invasion	(205)
	HCG18	Upregulated	miR-370-3p	Promotes cell proliferation, invasion, and migration	(206)
	LINC00893	Downregulated	miR-3173-5p	Tumor suppressor	(207)
	MIR22HG	Downregulated	miR-9-3p	Inhibits cell proliferation/Promotes apoptosis	(208)
	MAGI2-AS3	Downregulated	miR-424-5p/COP1	Inhibits cell proliferation	(209)
	ADAMTS9-AS1	Downregulated	miR-142-5p	Inhibits progression	(210)
	GAS5	Downregulated	miR-320a/RAB21	Inhibits viability and migration	(211)
	EMX2OS	Downregulated	FUS/TCF12	Inhibits tumor growth	(212)
	Erbb4-IR	Downregulated	miR-21	Promotes proliferation and apoptosis	(213)
	FER1L4	Downregulated	FBXW7/miR-92a-3p	Inhibits cell proliferation/Promotes cell apoptosis	(212)
	BLACAT1	Downregulated	DNMT1/HDAC1/ EZH2/MDM2/miR-361	Inhibits cells growth/Promotes cell death	(214)
	LINC00908	Downregulated	miR-483-5p/TSPYL5	Inhibits progression.	(215)
	DGCR5	Downregulated	TGF-b1	Inhibits cells stemness	(216)
	MAGI2-AS3	Downregulated	miR-142-3p	Inhibits proliferation, migration, and invasion	(217)
**Bladder**	UCA1	Upregulated	CDKN2B/EP300/TGFβ-2	Proliferation, migration, invasion, cell cycle, apoptosis, glycolysis, mitochondria and cisplatin resistance	(218)
	H19	Upregulated	miR-29b-3p/DNMT3B/miR-675/p53	Proliferation, migration, invasion, cell cycle and apoptosis	(219)

	Lnc-MUC20-9	Upregulated	ROCK1	Proliferation, migration, invasion and apoptosis	(220)
	LncARSR	Upregulated	miR-129-5p/SOX4	Proliferation, migration, invasion and EMT	(221)
	lncRNA ROR	Upregulated	ZEB1	Proliferation, migration, apoptosis and cell cycle	(222)
	AWPPH	Upregulated	EZH2/SMAD4	Proliferation, migration and invasion	(223)
	LSINCT5	Upregulated	NCYM	Migration and invasion	(224)
	AATBC	Upregulated	cyclinD1/CDK4/p18/Rb	Proliferation, cell cycle and apoptosis	(225)
	DUXAP8	Upregulated	PTEN	Proliferation	(226)
	SOX2OT	Upregulated	SOX2	Proliferation and apoptosis	(227)
	OXCT1-AS1	Upregulated	miR-455-5p/JAK1	Proliferation, migration and stemness	(228)
	XIST	Upregulated	TET1/p53	Proliferation, migration and apoptosis	(229)
	CASC8	Upregulated	FGFR1/LDHA	Proliferation and invasion	(230)
	LINC01638	Upregulated	ROCK2	Proliferation and glycolysis	(231)
	LNMAT1	Upregulated	hnRNPL/CCL2	EMT	(232)
	LNMAT2	Upregulated	hnRNPA2B1/PROX1	Lymphatic metastasis	(233)
	MST1P2	Upregulated	miR-133b/Sirt1/p53	Prognostic factor	(234)
	MIR503HG	Downregulated	ZEB1/Snail/N-cadherin/vimentin/E-cadherin	Proliferation, migration, invasion, cell cycle and apoptosis	(235)
	MEG3	Downregulated	miR-96/TPM1/miR-494/PTEN/LC3-I/II/miR-27a/PHLPP2	Proliferation, migration, invasion, cell cycle, apoptosis and autophagy	(236)
	lncRNA miR143HG	Downregulated	miR-1275/AXIN2	Proliferation, migration, apoptosis and cell cycle	(237)
	CASC2	Downregulated	β-catenin	Proliferation, migration, invasion and apoptosis	(238)
	PTENP1	Downregulated	miR-17/PTEN	Proliferation, migration, invasion and apoptosis	(239)
	LINC00312	Downregulated	miR-197-3p/MMP2/9/TIMP2	Proliferation, migration and invasion	(240)
	LINC00641	Downregulated	miR-197-3p/KLF10/PTEN/PI3K	Proliferation, migration and invasion	(241)
	LOC572558	Downregulated	MDM2/p53	Proliferation, migration and cell cycle	(242)
	NBAT1	Downregulated	miR-21/SOCS6	Proliferation, migration and cell cycle	(243)
	MT1JP	Downregulated	miR-214-3p	Proliferation, invasion and cell cycle	(244)
	DGCR5	Downregulated	ARID1A/P21	Proliferation and cell cycle	(245)
	GAS5	Downregulated	CCL1/Bcl2/CDK6	Proliferation and apoptosis	(246)
	MBNL1-AS1	Downregulated	miR-135a/PHLPP2/FOXO1	Proliferation and apoptosis	(247)
	TP73-AS1	Downregulated	Vimentin/MMP-2/9/snail/E-cadherin	Proliferation	(248)
	lncRNA-LBCS	Downregulated	hnRNPK/EZH2/SOX2/H3K27	Proliferation	(249)
**Cervical**	HOTAIR	Upregulated	miR-143-3p/miR-23b/miR-17-5p/MAPK1/BCL-2	Cell growth, proliferation, survival, EMT metastasis and invasion	(250)
	H19	Upregulated	miR-106/miR-194/miR-138-5p/miR-675/miR-140/miR-200/MILK	Cell migration and proliferation	(251)
	MALAT1	Upregulated	miR-145	Cell migration, proliferation, vascular invasion, EMT and metastasis	(252)

	CCHE1	Upregulated	PCNA	Cell proliferation and metastasis	(253)
	ANRIL	Upregulated	miR-186	Cell proliferation and metastasis	(254)
	BLACAT1	Upregulated	miR-143/miR-424	Cell proliferation and migration	(255)
	XIST	Upregulated	miR-140-5p	Cell proliferation, invasion, EMT and migration	(256)
	SPRY4-IT1	Upregulated	miR-101-3p/ZEB1	Cell proliferation and migration	(257)
	DLX6-AS1	Upregulated	miR-16-5p/miR-199a/ARPP19/FUS	Cell proliferation and invasion	(258)
	HOXD-AS1	Upregulated	miR-130a-3p/ZEB1	Cell growth, migration and invasion	(259)
	CRNDE	Upregulated	miR-183/CCNB1/PUMA/P53	Cell proliferation and apoptosis	(260)
	PVT1	Upregulated	Smad3/miR-140-5p/miR-424/miR-195/miR-200b/EZH2/TGF-β1	Cell growth, proliferation and metastasis	(261)
	MEG3	Downregulated	miR-21-5p/BCL-2/Bax/P21/P-STAT3	Metastasis and apoptosis	(262)
	GAS5	Downregulated	miR-196a/miR-205	Cell proliferation, invasion and apoptosis	(263)
**Ovarian**	LncRNA-ATB	Upregulated	miR-204-3p/NID1	Promotes invasion and tumorigenesis	(264)
	CASC9	Upregulated	miR-758-3p/LIN7A	Promotes proliferation, migration and invasion	(265)
	CCAT1	Upregulated	miR-152/ADAM17/miR-130b/STAT3/ZEB1	Promotes migration, invasion and metastasis	(266)
	CCAT2	Upregulated	miR-424	Promotes proliferation and progression	(267)
	CDKN2B-AS1	Upregulated	miR-411-3p/HIF1A	Promotes migration, invasion and metastasis	(268)
	DANCR	Upregulated	miR-145/VEGF	Promotes invasion	(269)
	DLEU1	Upregulated	miR-490-3p/CDK1	Promotes migration and invasion	(270)
	DLX6-AS1	Upregulated	miR-195-5p/FHL2	Promotes migration, invasion and EMT	(271)
	DQ786243	Upregulated	miR-506/CREB1	Promotes migration, invasion and EMT	(272)
	EMX2OS	Upregulated	miR-654/AKT3	Promotes invasion	(273)
	FLVCR1-AS1	Upregulated	miR-513/YAP1	Promotes migration, invasion and EMT	(274)
	GIHCG	Upregulated	miR-429	Promotes cell cycle	(275)
	H19	Upregulated	miR-370-3p	Promotes TGF-β-induced EMT	(276)
	HAGLROS	Upregulated	miR-100/ZNRF2	Poor prognosis	(277)
	CREB1-HAS2-AS1	Upregulated	miR-466/RUNX2	Promotes invasion and tumor growth	(278)
	HOST2	Upregulated	let-7b	Promotes migration, invasion and metastasis	(279)
	HOTAIR	Upregulated	miR-1/miR-214-3p/miR-330-5p/MAPK1/miR-214/miR-217/PIK3R3	Promotes proliferation, migration and invasion	(280)
	HOXD-AS1	Upregulated	miR-608/FZD4	Promotes migration and invasion	(281)
	HULC	Upregulated	miR-125a-3p	Promotes migration and invasion	(282)
	KCNQ1OT1	Upregulated	miR-212-3p/LCN2/miR-142-5p/CAPN10	Promotes migration and invasion	(283)
	LEF1-AS1	Upregulated	miR-1285-3p	Promotes migration, invasion and metastasis	(284)
	LINC00152	Upregulated	miR-125b/MCL-1	Poor prognosis	(285)
	LINC00161	Upregulated	miR-128/MAPK1	Drug resistance	(286)
	LINC00319	Upregulated	miR-423-5p/NACC1	Promotes proliferation, migration and invasion	(287)
	LINC00339	Upregulated	miR-148a-3p/ROCK1	Promotes migration and invasion	(288)

	LINC00460	Upregulated	miR-338-3p	Promotes migration, invasion and metastasis	(289)
	ESR1-LINC00511	Upregulated	miR-424/miR-370	Promotes proliferation and invasion	(290)
	LINC00963	Upregulated	miR-378/CHI3L1	Promotes migration	(291)
	LINC01118	Upregulated	miR-134/ABCC1	Promotes migration and invasion	(292)
	LUCAT1	Upregulated	miR-612/HOXA13	Promotes metastasis	(293)
	MALAT1	Upregulated	miR-506/miR-200c/miR-143-3p/CMPK/miR-211/PHF19/miR-503-5p	Promotes proliferation, migration, invasion and metastasis	(294)
	MLK7-AS1	Upregulated	miR-375/YAP1	Promotes invasion and metastasis	(295)
	MIR4435-2HG	Upregulated	miR-128-3p/CDK14	Promotes migration and invasion	(296)
	NCK1-AS1	Upregulated	miR-137/NCK1	Promotes migration and invasion	(297)
	Hur>NEAT1	Upregulated	miR-124-3p	Promotes migration, invasion and metastasis	(298)
	NEAT1	Upregulated	miR-382-3p/ROCK1/miR-770-5p/PARP1	Promotes migration, invasion and metastasis	(299)
	LIN28B>NEAT1	Upregulated	miR-506	Promotes migration and invasion	(300)
	NORAD	Upregulated	miR-199a-3p	Promotes proliferation, migration and invasion	(301)
	Lnc-OC1	Upregulated	miR-34a, miR-34c	Promotes migration and invasion	(302)
	OIP5-AS1	Upregulated	miR-137/ZNF217	Promotes migration and invasion	(303)
	PCAT-1	Upregulated	miR-129-5p	Promotes proliferation/Inhibits apoptosis	(304)
	PTAF(LINC00922)	Upregulated	miR-25/SNAI2	Promotes invasion and metastasis	(305)
	PTAR (AP000695.4)	Upregulated	miR-101/ZEB1	Promotes migration and metastasis	(306)
	PTAL	Upregulated	miR-101/FN1	Promotes invasion and metastasis	(307)
	PVT1	Upregulated	miR-133a/miR-214/FOXO4/miR-140	Promotes proliferation, migration and invasion	(308)
	RHPN1-AS1	Upregulated	miR-1299	Promotes migration and invasion	(309)
	LncRNA-ROR	Upregulated	miR-145/FLNB	Promotes migration and invasion	(310)
	SCAMP1	Upregulated	miR-137/CXCL12	Promotes invasion	(311)
	SNHG12	Upregulated	miR-129/SOX4	Promotes migration and metastasis	(312)
	TDRG1	Upregulated	miR-93/RhoC	Promotes migration and invasion	(313)
	TMPO-AS1	Upregulated	miR-200c/TMEFF2	Promotes invasion	(314)
	TTN-AS1	Upregulated	miR-15b-5p/FBXW7	Inhibits proliferation/Promotes apoptosis	(315)
	TUG1	Upregulated	miR-29b-3p/MDM2	Promotes migration and invasion	(316)
	UCA1	Upregulated	miR-129/ABCB1	Promotes proliferation, migration and invasion	(317)
	ZFAS1	Upregulated	miR-150-5p/Sp1	Promotes proliferation and migration	(318)
	ADAMTS9-AS2	Downregulated	miR-182-5p/FOXF2	Inhibits invasion and EMT	(319)
	EPB41L4A-AS2	Downregulated	miR-103a/RUNX1T1	Inhibits migration and invasion	(320)
	GAS5	Downregulated	miR-21/SPRY2/miR-196a-5p/HOXA5	Inhibits proliferation, migration and invasion	(321)
	HAND2-AS1	Downregulated	miR-340-5p/BCL2L11	Inhibits migration and invasion	(322)
	HOTAIRM1	Downregulated	miR-106a-5p/ARHGAP24	Inhibits migration, invasion and metastasis	(323)
	LINC01088	Downregulated	miR-24-1-5p/PAK4	Inhibits proliferation	(324)

	LINC01125	Downregulated	miR-1972	Inhibits proliferation/Promotes cisplatin sensitivity	(325)
	LINC01133	Downregulated	miR-205/LRRK2	Inhibits invasion and migration	(326)
	MAGI2-AS3	Downregulated	miR-525-5p/MXD1/miR-15-5p/PTEN	Inhibits cell cycle, migration and invasion	(327)
	MIR503HG	Downregulated	miR-31-5p	Inhibits invasion and migration	(328)
	MORT	Downregulated	miR-21	Inhibits proliferation	(329)
	WDFY3-AS2	Downregulated	miR-18a/RORA	Inhibits migration, invasion and EMT	(330)
**Pancreatic**	C9orf139	Upregulated	miR-663a/Sox12	Poor clinicopathological feature	(331)
	CRNDE	Upregulated	miR-384	Oncogene	(332)
	HOTAIR	Upregulated	miR-34a	Promotes proliferation and invasion	(333)
	HOTTIP	Upregulated	HOXA10/HOXB2/ HOXA11/HOXA9/HOXA1	Promotes proliferation, apoptosis and migration	(334)
	HOXA-AS2	Upregulated	EZH2/LSD1	Oncogene	(335)
	L INC 00976	Upregulated	miR-137/OTUD7B	Promotes invasion, migration and proliferation	(336)
	LINC00462	Upregulated	miR-665/TGFBR1/TGFBR2	Promotes EMT and cell proliferation/Inhibits cell apoptosis	(337)
	LINC01559	Upregulated	miR-1343-3p/RAF1	Promotes proliferation and metastasis	(338)
	LINC00152	Upregulated	miR-150	Promotes cells progressions	(339)
	LINC00958	Upregulated	miR-330-5p	Promotes EMT process and metastasis	(340)
	LUCAT1	Upregulated	miR-539	Promotes invasion	(341)
	LINC00994	Upregulated	miR-765-3p/RUNX2	Oncogene	(342)
	LINC01207	Upregulated	miR-143-5p	Promotes apoptosis and autophagy activity	(343)
	MACC1-AS1	Upregulated	PAX8	Promotes proliferation and metastasis	(344)
	OIP5-AS1	Upregulated	miR-429/FOXD1	Promotes EMT process, invasion and proliferation	(345)
	PVT1	Upregulated	miR-519d-3p	Promotes progression	(346)
	SBF2-AS1	Upregulated	miR-122-5p	Oncogene	(347)
	SNHG7	Upregulated	miR-146b-5/Robo1	Promotes progression	(348)
	SNHG12	Upregulated	miR-320b	Promotes invasion, EMT and proliferation	(349)
	SNHG14	Upregulated	miR-613	Promotes progression	(350)
	SNHG15	Upregulated	zeste homolog 2	Oncogene	(351)
	SPRY4-IT1	Upregulated	Cdc20	Promotes progression	(352)
	TP73 -AS1	Upregulated	miR-141	Promotes migration, invasion and proliferation	(353)
	UCA1	Upregulated	miR-96/FOXO3	Oncogene	(354)
	XIST	Upregulated	miR-133a/EGFR	Promotes proliferation	(355)
	ZEB2-AS1	Upregulated	miR-204/HMGB1	Promotes proliferation and invasion	(356)
	ENST00000480739	Downregulated	OS-9	Inhibits tumor metastasis and progression	(357)
	LINC01111	Downregulated	miR-3924	Tumor-suppressor	(358)
	LINC01963	Downregulated	miR-641/TMEFF2	Inhibits progression	(359)

	DGCR5	Downregulated	miR-27a-3p/BNIP3	Promotes apoptosis	(360)
	MEG3	Downregulated	PI3K protein	Tumor-suppressor	(361)
	GAS5	Downregulated	miR-32-5p	Tumor-suppressor	(362)
	LINC00261	Downregulated	miR-23a-3p	Promotes progression	(363)

References

1. Haslauer T, Greil R, Zaborsky N, Geisberger R. CAR T-Cell Therapy in Hematological Malignancies. *Int J Mol Sci*. (2021) 22(16). doi: 10.3390/ijms22168996.
2. Baulu E, Gardet C, Chuvin N, Depil S. TCR-engineered T cell therapy in solid tumors: State of the art and perspectives. *Sci Adv*. (2023) 9(7):eadf3700. doi: 10.1126/sciadv.adf3700.
3. Zhao Y, Deng J, Rao S, Guo S, Shen J, Du F, et al. Tumor Infiltrating Lymphocyte (TIL) Therapy for Solid Tumor Treatment: Progressions and Challenges. *Cancers (Basel)*. (2022) 14(17). doi: 10.3390/cancers14174160.
4. Moscarelli J, Zahavi D, Maynard R, Weiner LM. The Next Generation of Cellular Immunotherapy: Chimeric Antigen Receptor-Natural Killer Cells. *Transplant Cell Ther*. (2022) 28(10):650-6. doi: 10.1016/j.jtct.2022.06.025.
5. Zhang Y, Liu Z, Wei W, Li Y. TCR engineered T cells for solid tumor immunotherapy. *Exp Hematol Oncol*. (2022) 11(1):38. doi: 10.1186/s40164-022-00291-0.
6. Reiss KA, Yuan Y, Ueno NT, Johnson ML, Gill S, Dees EC, et al. A phase 1, first-in-human (FIH) study of the anti-HER2 CAR macrophage CT-0508 in subjects with HER2 overexpressing solid tumors. *J Clin Oncol*. (2022) 40(16_suppl):2533-. doi: 10.1200/JCO.2022.40.16_suppl.2533.
7. Daei Sorkhabi A, Mohamed Khosroshahi L, Sarkesh A, Mardi A, Aghebati-Maleki A, Aghebati-Maleki L, et al. The current landscape of CAR T-cell therapy for solid tumors: Mechanisms, research progress, challenges, and counterstrategies. *Front Immunol*. (2023) 14:1113882. doi: 10.3389/fimmu.2023.1113882.
8. Bluestone JA, McKenzie BS, Beilke J, Ramsdell F. Opportunities for Treg cell therapy for the treatment of human disease. *Front Immunol*. (2023) 14:1166135. doi: 10.3389/fimmu.2023.1166135.
9. Bhokisham N, Laudermilch E, Traeger LL, Bonilla TD, Ruiz-Estevez M, Becker JR. CRISPR-Cas System: The Current and Emerging Translational Landscape. *Cells*. (2023) 12(8). doi: 10.3390/cells12081103.
10. Perez C, Gruber I, Arber C. Off-the-Shelf Allogeneic T Cell Therapies for Cancer: Opportunities and Challenges Using Naturally Occurring "Universal" Donor T Cells. *Front Immunol*. (2020) 11:583716. doi: 10.3389/fimmu.2020.583716.
11. Zhou JZ, Jou J, Cohen E. Vaccine Strategies for Human Papillomavirus-Associated Head and Neck Cancers. *Cancers (Basel)*. (2021) 14(1). doi: 10.3390/cancers14010033.
12. Monie A, Hung CF, Roden R, Wu TC. Cervarix: a vaccine for the prevention of HPV 16, 18-associated cervical cancer. *Biologics*. (2008) 2(1):97-105. doi: 10.2147/btt.s12160259.
13. Anassi E, Ndefo UA. Sipuleucel-T (provenge) injection: the first immunotherapy agent (vaccine) for hormone-refractory prostate cancer. *P T*. (2011) 36(4):197-202.
14. Cowey CL, Liu FX, Black-Shinn J, Stevinson K, Boyd M, Frytak JR, et al. Pembrolizumab Utilization and Outcomes for Advanced Melanoma in US Community Oncology Practices. *J Immunother*. (2018) 41(2):86-95. doi: 10.1097/cji.0000000000000204.
15. Chen YJ, Abila B, Mostafa Kamel Y. CAR-T: What Is Next? *Cancers (Basel)*. (2023) 15(3). doi: 10.3390/cancers15030663.
16. He Q, Gao H, Tan D, Zhang H, Wang JZ. mRNA cancer vaccines: Advances, trends and challenges. *Acta Pharm Sin B*. (2022) 12(7):2969-89. doi: 10.1016/j.apsb.2022.03.011.
17. Fan T, Zhang M, Yang J, Zhu Z, Cao W, Dong C. Therapeutic cancer vaccines: advancements, challenges, and prospects. *Signal Transduct Target Ther*. (2023) 8(1):450. doi: 10.1038/s41392-023-01674-3.

18. Blumberg BS. Primary and secondary prevention of liver cancer caused by HBV. *Front Biosci (Schol Ed)*. (2010) 2(2):756-63. doi: 10.2741/s98.
19. Cheng L, Wang Y, Du J. Human Papillomavirus Vaccines: An Updated Review. *Vaccines (Basel)*. (2020) 8(3). doi: 10.3390/vaccines8030391.
20. Garmaroudi GA, Karimi F, Naeini LG, Kokabian P, Givtaj N. Therapeutic Efficacy of Oncolytic Viruses in Fighting Cancer: Recent Advances and Perspective. *Oxid Med Cell Longev*. (2022) 2022:3142306. doi: 10.1155/2022/3142306.
21. Gholami M, Klashami ZN, Ebrahimi P, Mahboobipour AA, Farid AS, Vahidi A, et al. Metformin and long non-coding RNAs in breast cancer. *J Transl Med*. (2023) 21(1):155. doi: 10.1186/s12967-023-03909-x.
22. Li Y, Song Y, Wang Z, Zhang Z, Lu M, Wang Y. Long Non-coding RNA LINC01787 Drives Breast Cancer Progression via Disrupting miR-125b Generation. *Front Oncol*. (2019) 9:1140. doi: 10.3389/fonc.2019.01140.
23. Zhou W, Gong J, Chen Y, Chen J, Zhuang Q, Cao J, et al. Long noncoding RNA LINC00899 suppresses breast cancer progression by inhibiting miR-425. *Aging*. (2019) 11(22):10144-53. doi: 10.18632/aging.102426.
24. Hu P, Chu J, Wu Y, Sun L, Lv X, Zhu Y, et al. NBAT1 suppresses breast cancer metastasis by regulating DKK1 via PRC2. *Oncotarget*. (2015) 6(32):32410-25. doi: 10.18632/oncotarget.5609.
25. Gupta RA, Shah N, Wang KC, Kim J, Horlings HM, Wong DJ, et al. Long non-coding RNA HOTAIR reprograms chromatin state to promote cancer metastasis. *Nature*. (2010) 464(7291):1071-6. doi: 10.1038/nature08975.
26. Yang F, Shen Y, Zhang W, Jin J, Huang D, Fang H, et al. An androgen receptor negatively induced long non-coding RNA ARNILA binding to miR-204 promotes the invasion and metastasis of triple-negative breast cancer. *Cell Death Differ*. (2018) 25(12):2209-20. doi: 10.1038/s41418-018-0123-6.
27. Chen DQ, Zheng XD, Cao Y, He XD, Nian WQ, Zeng XH, et al. Long non-coding RNA LINC00628 suppresses the growth and metastasis and promotes cell apoptosis in breast cancer. *Eur Rev Med Pharmacol Sci*. (2017) 21(2):275-83.
28. Zheng R, Lin S, Guan L, Yuan H, Liu K, Liu C, et al. Long non-coding RNA XIST inhibited breast cancer cell growth, migration, and invasion via miR-155/CDX1 axis. *Biochem Biophys Res Commun*. (2018) 498(4):1002-8. doi: 10.1016/j.bbrc.2018.03.104.
29. De Santis C, Götte M. The Role of microRNA Let-7d in Female Malignancies and Diseases of the Female Reproductive Tract. *Int J Mol Sci*. (2021) 22(14). doi: 10.3390/ijms22147359.
30. Gao Z, Wang H, Li H, Li M, Wang J, Zhang W, et al. Long non-coding RNA CASC2 inhibits breast cancer cell growth and metastasis through the regulation of the miR-96-5p/SYVN1 pathway. *Int J Oncol*. (2018) 53(5):2081-90. doi: 10.3892/ijo.2018.4522.
31. Yuan C, Luo X, Duan S, Guo L. Long noncoding RNA LINC00115 promotes breast cancer metastasis by inhibiting miR-7. *FEBS Open Bio*. (2020) 10(7):1230-7. doi: 10.1002/2211-5463.12842.
32. Si H, Chen P, Li H, Wang X. Long non-coding RNA H19 regulates cell growth and metastasis via miR-138 in breast cancer. *Am J Transl Res*. (2019) 11(5):3213-25.
33. Bukhari I, Khan MR, Hussain MA, Thorne RF, Yu Y, Zhang B, et al. PINTology: A short history of the lncRNA LINC-PINT in different diseases. *Wiley Interdiscip Rev RNA*. (2022) 13(4):e1705. doi: 10.1002/wrna.1705.

34. Jiang M, Xiao Y, Liu D, Luo N, Gao Q, Guan Y. Overexpression of long noncoding RNA LINC01296 indicates an unfavorable prognosis and promotes tumorigenesis in breast cancer. *Gene*. (2018) 675:217-24. doi: 10.1016/j.gene.2018.07.004.
35. Shen S, Wang Y, Zhang Y, Dong Z, Xing J. Long Non-coding RNA Small Nucleolar RNA Host Gene 14, a Promising Biomarker and Therapeutic Target in Malignancy. *Front Cell Dev Biol*. (2021) 9:746714. doi: 10.3389/fcell.2021.746714.
36. Song R, Zhang J, Huang J, Hai T. Long non-coding RNA GHET1 promotes human breast cancer cell proliferation, invasion and migration via affecting epithelial mesenchymal transition. *Cancer Biomark*. (2018) 22(3):565-73. doi: 10.3233/cbm-181250.
37. Xia E, Shen Y, Bhandari A, Zhou X, Wang Y, Yang F, et al. Long non-coding RNA LINC00673 promotes breast cancer proliferation and metastasis through regulating B7-H6 and epithelial-mesenchymal transition. *Am J Cancer Res*. (2018) 8(7):1273-87.
38. Guo F, Guo L, Li Y, Zhou Q, Li Z. MALAT1 is an oncogenic long non-coding RNA associated with tumor invasion in non-small cell lung cancer regulated by DNA methylation. *Int J Clin Exp Pathol*. (2015) 8(12):15903-10.
39. Jiang C, Yang Y, Yang Y, Guo L, Huang J, Liu X, et al. Long Noncoding RNA (lncRNA) HOTAIR Affects Tumorigenesis and Metastasis of Non-Small Cell Lung Cancer by Upregulating miR-613. *Oncol Res*. (2018) 26(5):725-34. doi: 10.3727/096504017x15119467381615.
40. Fu Y, Wang W, Li X, Liu Y, Niu Y, Zhang B, et al. LncRNA H19 interacts with S-adenosylhomocysteine hydrolase to regulate LINE-1 Methylation in human lung-derived cells exposed to Benzo[a]pyrene. *Chemosphere*. (2018) 207:84-90. doi: 10.1016/j.chemosphere.2018.05.048.
41. Guo D, Wang Y, Ren K, Han X. Knockdown of LncRNA PVT1 inhibits tumorigenesis in non-small-cell lung cancer by regulating miR-497 expression. *Exp Cell Res*. (2018) 362(1):172-9. doi: 10.1016/j.yexcr.2017.11.014.
42. Naemura M, Murasaki C, Inoue Y, Okamoto H, Kotake Y. Long Noncoding RNA ANRIL Regulates Proliferation of Non-small Cell Lung Cancer and Cervical Cancer Cells. *Anticancer Res*. (2015) 35(10):5377-82.
43. Chen Z, Li JL, Lin S, Cao C, Gimbrone NT, Yang R, et al. cAMP/CREB-regulated LINC00473 marks LKB1-inactivated lung cancer and mediates tumor growth. *J Clin Invest*. (2016) 126(6):2267-79. doi: 10.1172/jci85250.
44. Yu T, Zhao Y, Hu Z, Li J, Chu D, Zhang J, et al. MetaLnc9 Facilitates Lung Cancer Metastasis via a PGK1-Activated AKT/mTOR Pathway. *Cancer Res*. (2017) 77(21):5782-94. doi: 10.1158/0008-5472.Can-17-0671.
45. Hou Z, Zhao W, Zhou J, Shen L, Zhan P, Xu C, et al. A long noncoding RNA Sox2ot regulates lung cancer cell proliferation and is a prognostic indicator of poor survival. *Int J Biochem Cell Biol*. (2014) 53:380-8. doi: 10.1016/j.biocel.2014.06.004.
46. Ge X, Li GY, Jiang L, Jia L, Zhang Z, Li X, et al. Long noncoding RNA CAR10 promotes lung adenocarcinoma metastasis via miR-203/30/SNAI axis. *Oncogene*. (2019) 38(16):3061-76. doi: 10.1038/s41388-018-0645-x.
47. Jiang W, Zhang D, Xu B, Wu Z, Liu S, Zhang L, et al. Long non-coding RNA BANCR promotes proliferation and migration of lung carcinoma via MAPK pathways. *Biomed Pharmacother*. (2015) 69:90-5. doi: 10.1016/j.biopha.2014.11.027.
48. Han L, Zhang EB, Yin DD, Kong R, Xu TP, Chen WM, et al. Low expression of long noncoding RNA PANDAR predicts a poor prognosis of non-small cell lung cancer and affects cell apoptosis by regulating Bcl-2. *Cell Death Dis*. (2015) 6(2):e1665. doi: 10.1038/cddis.2015.30.

49. Su W, Feng S, Chen X, Yang X, Mao R, Guo C, et al. Silencing of Long Noncoding RNA MIR22HG Triggers Cell Survival/Death Signaling via Oncogenes YBX1, MET, and p21 in Lung Cancer. *Cancer Res*. (2018) 78(12):3207-19. doi: 10.1158/0008-5472.Can-18-0222.
50. Liu J, Wan L, Lu K, Sun M, Pan X, Zhang P, et al. The Long Noncoding RNA MEG3 Contributes to Cisplatin Resistance of Human Lung Adenocarcinoma. *PLoS One*. (2015) 10(5):e0114586. doi: 10.1371/journal.pone.0114586.
51. Wang H, Wang L, Zhang G, Lu C, Chu H, Yang R, et al. MALAT1/miR-101-3p/MCL1 axis mediates cisplatin resistance in lung cancer. *Oncotarget*. (2018) 9(7):7501-12. doi: 10.18632/oncotarget.23483.
52. Xi Z, Si J, Nan J. LncRNA MALAT1 potentiates autophagy-associated cisplatin resistance by regulating the microRNA-30b/autophagy-related gene 5 axis in gastric cancer. *Int J Oncol*. (2019) 54(1):239-48. doi: 10.3892/ijo.2018.4609.
53. Hang Q, Sun R, Jiang C, Li Y. Notch 1 promotes cisplatin-resistant gastric cancer formation by upregulating lncRNA AK022798 expression. *Anticancer Drugs*. (2015) 26(6):632-40. doi: 10.1097/cad.0000000000000227.
54. Wang Z, Wang Q, Xu G, Meng N, Huang X, Jiang Z, et al. The long noncoding RNA CRAL reverses cisplatin resistance via the miR-505/CYLD/AKT axis in human gastric cancer cells. *RNA Biol*. (2020) 17(11):1576-89. doi: 10.1080/15476286.2019.1709296.
55. Wang S, Chen W, Yu H, Song Z, Li Q, Shen X, et al. lncRNA ROR Promotes Gastric Cancer Drug Resistance. *Cancer Control*. (2020) 27(1):1073274820904694. doi: 10.1177/1073274820904694.
56. Zeng L, Liao Q, Zou Z, Wen Y, Wang J, Liu C, et al. Long Non-Coding RNA XLOC_006753 Promotes the Development of Multidrug Resistance in Gastric Cancer Cells Through the PI3K/AKT/mTOR Signaling Pathway. *Cell Physiol Biochem*. (2018) 51(3):1221-36. doi: 10.1159/000495499.
57. He W, Liang B, Wang C, Li S, Zhao Y, Huang Q, et al. MSC-regulated lncRNA MACC1-AS1 promotes stemness and chemoresistance through fatty acid oxidation in gastric cancer. *Oncogene*. (2019) 38(23):4637-54. doi: 10.1038/s41388-019-0747-0.
58. Zhou Z, Lin Z, He Y, Pang X, Wang Y, Ponnusamy M, et al. The Long Noncoding RNA D63785 Regulates Chemotherapy Sensitivity in Human Gastric Cancer by Targeting miR-422a. *Mol Ther Nucleic Acids*. (2018) 12:405-19. doi: 10.1016/j.omtn.2018.05.024.
59. Zhang C, Qian H, Liu K, Zhao W, Wang L. A Feedback Loop Regulation Of LINC01433 And YAP Promotes Malignant Behavior In Gastric Cancer Cells. *Onco Targets Ther*. (2019) 12:7949-62. doi: 10.2147/ott.S222903.
60. Li H, Ma X, Yang D, Suo Z, Dai R, Liu C. PCAT-1 contributes to cisplatin resistance in gastric cancer through epigenetically silencing PTEN via recruiting EZH2. *J Cell Biochem*. (2020) 121(2):1353-61. doi: 10.1002/jcb.29370.
61. Xin L, Zhou Q, Yuan YW, Zhou LQ, Liu L, Li SH, et al. METase/lncRNA HULC/FoxM1 reduced cisplatin resistance in gastric cancer by suppressing autophagy. *J Cancer Res Clin Oncol*. (2019) 145(10):2507-17. doi: 10.1007/s00432-019-03015-w.
62. Jia J, Zhan D, Li J, Li Z, Li H, Qian J. The contrary functions of lncRNA HOTAIR/miR-17-5p/PTEN axis and Shenqifuzheng injection on chemosensitivity of gastric cancer cells. *J Cell Mol Med*. (2019) 23(1):656-69. doi: 10.1111/jcmm.13970.
63. Song H, Xu Y, Shi L, Xu T, Fan R, Cao M, et al. LncRNA THOR increases the stemness of gastric cancer cells via enhancing SOX9 mRNA stability. *Biomed Pharmacother*. (2018) 108:338-46. doi: 10.1016/j.biopha.2018.09.057.

64. Wu X, Zheng Y, Han B, Dong X. Long noncoding RNA BLACAT1 modulates ABCB1 to promote oxaliplatin resistance of gastric cancer via sponging miR-361. *Biomed Pharmacother*. (2018) 99:832-8. doi: 10.1016/j.biopha.2018.01.130.
65. Zhang X, Bo P, Liu L, Zhang X, Li J. Overexpression of long non-coding RNA GHET1 promotes the development of multidrug resistance in gastric cancer cells. *Biomed Pharmacother*. (2017) 92:580-5. doi: 10.1016/j.biopha.2017.04.111.
66. Lan WG, Xu DH, Xu C, Ding CL, Ning FL, Zhou YL, et al. Silencing of long non-coding RNA ANRIL inhibits the development of multidrug resistance in gastric cancer cells. *Oncol Rep*. (2016) 36(1):263-70. doi: 10.3892/or.2016.4771.
67. Shang C, Guo Y, Zhang J, Huang B. Silence of long noncoding RNA UCA1 inhibits malignant proliferation and chemotherapy resistance to adriamycin in gastric cancer. *Cancer Chemother Pharmacol*. (2016) 77(5):1061-7. doi: 10.1007/s00280-016-3029-3.
68. Du P, Hu C, Qin Y, Zhao J, Patel R, Fu Y, et al. LncRNA PVT1 Mediates Antiapoptosis and 5-Fluorouracil Resistance via Increasing Bcl2 Expression in Gastric Cancer. *J Oncol*. (2019) 2019:9325407. doi: 10.1155/2019/9325407.
69. Wang Y, Zhang D, Wu K, Zhao Q, Nie Y, Fan D. Long noncoding RNA MRUL promotes ABCB1 expression in multidrug-resistant gastric cancer cell sublines. *Mol Cell Biol*. (2014) 34(17):3182-93. doi: 10.1128/mcb.01580-13.
70. Li M, Zhang YY, Shang J, Xu YD. LncRNA SNHG5 promotes cisplatin resistance in gastric cancer via inhibiting cell apoptosis. *Eur Rev Med Pharmacol Sci*. (2019) 23(10):4185-91. doi: 10.26355/eurrev_201905_17921.
71. Xu YD, Shang J, Li M, Zhang YY. LncRNA DANCR accelerates the development of multidrug resistance of gastric cancer. *Eur Rev Med Pharmacol Sci*. (2019) 23(7):2794-802. doi: 10.26355/eurrev_201904_17554.
72. Zhang Y, Li Q, Yu S, Zhu C, Zhang Z, Cao H, et al. Long non-coding RNA FAM84B-AS promotes resistance of gastric cancer to platinum drugs through inhibition of FAM84B expression. *Biochem Biophys Res Commun*. (2019) 509(3):753-62. doi: 10.1016/j.bbrc.2018.12.177.
73. Zhang J, Zhao B, Chen X, Wang Z, Xu H, Huang B. Silence of Long Noncoding RNA NEAT1 Inhibits Malignant Biological Behaviors and Chemotherapy Resistance in Gastric Cancer. *Pathol Oncol Res*. (2018) 24(1):109-13. doi: 10.1007/s12253-017-0233-3.
74. Shang C, Sun L, Zhang J, Zhao B, Chen X, Xu H, et al. Silence of cancer susceptibility candidate 9 inhibits gastric cancer and reverses chemoresistance. *Oncotarget*. (2017) 8(9):15393-8. doi: 10.18632/oncotarget.14871.
75. Mao Z, Wu Y, Zhou J, Xing C. Salinomycin reduces epithelial-mesenchymal transition-mediated multidrug resistance by modifying long noncoding RNA HOTTIP expression in gastric cancer cells. *Anticancer Drugs*. (2019) 30(9):892-9. doi: 10.1097/cad.0000000000000786.
76. Wu H, Liu B, Chen Z, Li G, Zhang Z. MSC-induced lncRNA HCP5 drove fatty acid oxidation through miR-3619-5p/AMPK/PGC1α/CEBPB axis to promote stemness and chemo-resistance of gastric cancer. *Cell Death Dis*. (2020) 11(4):233. doi: 10.1038/s41419-020-2426-z.
77. Li Y, Lv S, Ning H, Li K, Zhou X, Xv H, et al. Down-regulation of CASC2 contributes to cisplatin resistance in gastric cancer by sponging miR-19a. *Biomed Pharmacother*. (2018) 108:1775-82. doi: 10.1016/j.biopha.2018.09.181.
78. Han Y, Ye J, Wu D, Wu P, Chen Z, Chen J, et al. LEIGC long non-coding RNA acts as a tumor suppressor in gastric carcinoma by inhibiting the epithelial-to-mesenchymal transition. *BMC Cancer*. (2014) 14:932. doi: 10.1186/1471-2407-14-932.

79. Liu L, Yang J, Zhu X, Li D, Lv Z, Zhang X. Long noncoding RNA H19 competitively binds miR-17-5p to regulate YES1 expression in thyroid cancer. *Febs j*. (2016) 283(12):2326-39. doi: 10.1111/febs.13741.
80. Liang L, Xu J, Wang M, Xu G, Zhang N, Wang G, et al. LncRNA HCP5 promotes follicular thyroid carcinoma progression via miRNAs sponge. *Cell Death Dis*. (2018) 9(3):372. doi: 10.1038/s41419-018-0382-7.
81. Yuan Q, Liu Y, Fan Y, Liu Z, Wang X, Jia M, et al. LncRNA HOTTIP promotes papillary thyroid carcinoma cell proliferation, invasion and migration by regulating miR-637. *Int J Biochem Cell Biol*. (2018) 98:1-9. doi: 10.1016/j.biocel.2018.02.013.
82. Wang KC, Yang YW, Liu B, Sanyal A, Corces-Zimmerman R, Chen Y, et al. A long noncoding RNA maintains active chromatin to coordinate homeotic gene expression. *Nature*. (2011) 472(7341):120-4. doi: 10.1038/nature09819.
83. Wu DM, Wang S, Wen X, Han XR, Wang YJ, Shen M, et al. LncRNA SNHG15 acts as a ceRNA to regulate YAP1-Hippo signaling pathway by sponging miR-200a-3p in papillary thyroid carcinoma. *Cell Death Dis*. (2018) 9(10):947. doi: 10.1038/s41419-018-0975-1.
84. Zhou H, Sun Z, Li S, Wang X, Zhou X. LncRNA SPRY4-IT was concerned with the poor prognosis and contributed to the progression of thyroid cancer. *Cancer Gene Ther*. (2018) 25(1-2):39-46. doi: 10.1038/s41417-017-0003-0.
85. Huang J, Pan B, Xia G, Zhu J, Li C, Feng J. LncRNA SNHG15 regulates EGFR-TKI acquired resistance in lung adenocarcinoma through sponging miR-451 to upregulate MDR-1. *Cell Death Dis*. (2020) 11(7):525. doi: 10.1038/s41419-020-2683-x.
86. Du P, Liu F, Liu Y, Shao M, Li X, Qin G. Linc00210 enhances the malignancy of thyroid cancer cells by modulating miR-195-5p/IGF1R/Akt axis. *J Cell Physiol*. (2020) 235(2):1001-12. doi: 10.1002/jcp.29016.
87. Li D, Hao S, Zhang J. Long non-coding RNA UCA1 exerts growth modulation by miR-15a in human thyroid cancer TPC-1 cells. *Artif Cells Nanomed Biotechnol*. (2019) 47(1):1815-22. doi: 10.1080/21691401.2019.1606007.
88. Wang N, Yu Y, Xu B, Zhang M, Li Q, Miao L. Pivotal prognostic and diagnostic role of the long non-coding RNA colon cancer-associated transcript 1 expression in human cancer (Review). *Mol Med Rep*. (2019) 19(2):771-82. doi: 10.3892/mmr.2018.9721.
89. Lu HW, Liu XD. UCA1 promotes papillary thyroid carcinoma development by stimulating cell proliferation via Wnt pathway. *Eur Rev Med Pharmacol Sci*. (2018) 22(17):5576-82. doi: 10.26355/eurrev_201809_15821.
90. Li JH, Zhang SQ, Qiu XG, Zhang SJ, Zheng SH, Zhang DH. Long non-coding RNA NEAT1 promotes malignant progression of thyroid carcinoma by regulating miRNA-214. *Int J Oncol*. (2017) 50(2):708-16. doi: 10.3892/ijo.2016.3803.
91. Yang C, Liu Z, Chang X, Xu W, Gong J, Chai F, et al. NR2F1-AS1 regulated miR-423-5p/SOX12 to promote proliferation and invasion of papillary thyroid carcinoma. *J Cell Biochem*. (2020) 121(2):2009-18. doi: 10.1002/jcb.29435.
92. Ghafouri-Fard S, Askari A, Hussen BM, Rasul MF, Taheri M, Kiani A. A review on the role of LINC00152 in different disorders. *Pathol Res Pract*. (2023) 241:154274. doi: 10.1016/j.prp.2022.154274.
93. Liang M, Jia J, Chen L, Wei B, Guan Q, Ding Z, et al. LncRNA MCM3AP-AS1 promotes proliferation and invasion through regulating miR-211-5p/SPARC axis in papillary thyroid cancer. *Endocrine*. (2019) 65(2):318-26. doi: 10.1007/s12020-019-01939-4.

94. Gou Q, Gao L, Nie X, Pu W, Zhu J, Wang Y, et al. Long Noncoding RNA AB074169 Inhibits Cell Proliferation via Modulation of KHSRP-Mediated CDKN1a Expression in Papillary Thyroid Carcinoma. *Cancer Res*. (2018) 78(15):4163-74. doi: 10.1158/0008-5472.Can-17-3766.
95. Feng Z, Chen R, Huang N, Luo C. Long non-coding RNA ASMTL-AS1 inhibits tumor growth and glycolysis by regulating the miR-93-3p/miR-660/FOXO1 axis in papillary thyroid carcinoma. *Life Sci*. (2020) 244:117298. doi: 10.1016/j.lfs.2020.117298.
96. Qin Y, Sun W, Zhang H, Zhang P, Wang Z, Dong W, et al. LncRNA GAS8-AS1 inhibits cell proliferation through ATG5-mediated autophagy in papillary thyroid cancer. *Endocrine*. (2018) 59(3):555-64. doi: 10.1007/s12020-017-1520-1.
97. Liu Y, Yue P, Zhou T, Zhang F, Wang H, Chen X. LncRNA MEG3 enhances (131)I sensitivity in thyroid carcinoma via sponging miR-182. *Biomed Pharmacother*. (2018) 105:1232-9. doi: 10.1016/j.biopha.2018.06.087.
98. Wang XP, Shan C, Deng XL, Li LY, Ma W. Long non-coding RNA PAR5 inhibits the proliferation and progression of glioma through interaction with EZH2. *Oncol Rep*. (2017) 38(5):3177-86. doi: 10.3892/or.2017.5986.
99. Li D, Chai L, Yu X, Song Y, Zhu X, Fan S, et al. The HOTAIRM1/miR-107/TDG axis regulates papillary thyroid cancer cell proliferation and invasion. *Cell Death Dis*. (2020) 11(4):227. doi: 10.1038/s41419-020-2416-1.
100. Xiao J, Bing Z, Xiao G, Guan Y, Luan J. Long non-coding (lnc)RNA PAPAS overexpression inhibits tumor growth in papillary thyroid carcinoma by downregulating lncRNA HOTTIP. *Oncol Lett*. (2020) 19(3):2281-5. doi: 10.3892/ol.2020.11323.
101. Wei C, Wu W, Hou X, Yuan S. Study on Oil Distribution and Oil Content of Oil Bath Lubrication Bearings Based on MPS Method. *TRIBOL T*. (2022) 65(5):942-51. doi: 10.1080/10402004.2022.2113193.
102. Xu J, Wang J, Zhao M, Li C, Hong S, Zhang J. LncRNA LINC01018/miR-942-5p/KNG1 axis regulates the malignant development of glioma in vitro and in vivo. *CNS Neurosci Ther*. (2023) 29(2):691-711. doi: 10.1111/cns.14053.
103. Su H, Hailin Z, Dongdong L, Jiang Y, Shuncheng H, Shun Z, et al. Long non-coding RNA LINC01018 inhibits human glioma cell proliferation and metastasis by directly targeting miRNA-182-5p. *J Neurooncol*. (2022) 160(1):67-78. doi: 10.1007/s11060-022-04113-5.
104. Wang R, Wang J, Wang Y, Yang L. lncRNA TUSC7 sponges miR-10a-5p and inhibits BDNF/ERK pathway to suppress glioma cell proliferation and migration. *Aging*. (2023) 15(8):3021-34. doi: 10.18632/aging.204655.
105. Yu W, Ma L, Li X. DANCR promotes glioma cell autophagy and proliferation via the miR-33b/DLX6/ATG7 axis. *Oncol Rep*. (2023) 49(2). doi: 10.3892/or.2023.8476.
106. Shu B, Gan H, Wang C, Cao C, Tong H, Liang D. LINC01426 aggravates the malignant progression of glioma through miR-661/Mdm2 axis. *Brain Res Bull*. (2022) 188:110-21. doi: 10.1016/j.brainresbull.2022.06.012.
107. Yu Z, Che N, He Y, Zhang B. ceRNA network of lncRNA MIR210HG/miR-377-3p/LMX1A in malignant proliferation of glioma cells. *Genes Genomics*. (2022) 44(12):1445-55. doi: 10.1007/s13258-022-01312-2.
108. Li Z, Song Y, Zhang J. lncRNA PDCD4-AS1 Promotes the Progression of Glioma by Regulating miR-30b-3p/METTL7B Signaling. *Oxid Med Cell Longev*. (2023) 2023:3492480. doi: 10.1155/2023/3492480.

109. Jiang X, Zhang Y, Yuan Y, Jin Z, Zhai H, Liu B, et al. LncRNA GSCAR promotes glioma stem cell maintenance via stabilizing SOX2 expression. *Int J Biol Sci*. (2023) 19(6):1681-97. doi: 10.7150/ijbs.80873.
110. Wang J, Chen Y, Wang Q, Xu H, Jiang Q, Wang M, et al. LncRNA SPRY4-IT1 facilitates cell proliferation and angiogenesis of glioma via the miR-101-3p/EZH2/VEGFA signaling axis. *Cancer Med*. (2023) 12(6):7309-26. doi: 10.1002/cam4.5517.
111. Yuan JH, Yang F, Wang F, Ma JZ, Guo YJ, Tao QF, et al. A long noncoding RNA activated by TGF-β promotes the invasion-metastasis cascade in hepatocellular carcinoma. *Cancer Cell*. (2014) 25(5):666-81. doi: 10.1016/j.ccr.2014.03.010.
112. Cao C, Sun J, Zhang D, Guo X, Xie L, Li X, et al. The long intergenic noncoding RNA UFC1, a target of MicroRNA 34a, interacts with the mRNA stabilizing protein HuR to increase levels of β-catenin in HCC cells. *Gastroenterology*. (2015) 148(2):415-26.e18. doi: 10.1053/j.gastro.2014.10.012.
113. Yuan JH, Liu XN, Wang TT, Pan W, Tao QF, Zhou WP, et al. The MBNL3 splicing factor promotes hepatocellular carcinoma by increasing PXN expression through the alternative splicing of lncRNA-PXN-AS1. *Nat Cell Biol*. (2017) 19(7):820-32. doi: 10.1038/ncb3538.
114. Huang MD, Chen WM, Qi FZ, Xia R, Sun M, Xu TP, et al. Long non-coding RNA ANRIL is upregulated in hepatocellular carcinoma and regulates cell apoptosis by epigenetic silencing of KLF2. *J Hematol Oncol*. (2015) 8:50. doi: 10.1186/s13045-015-0146-0.
115. Lin YH, Wu MH, Huang YH, Yeh CT, Cheng ML, Chi HC, et al. Taurine up-regulated gene 1 functions as a master regulator to coordinate glycolysis and metastasis in hepatocellular carcinoma. *Hepatology*. (2018) 67(1):188-203. doi: 10.1002/hep.29462.
116. Zhu P, Wang Y, Huang G, Ye B, Liu B, Wu J, et al. lnc-β-Catm elicits EZH2-dependent β-catenin stabilization and sustains liver CSC self-renewal. *Nat Struct Mol Biol*. (2016) 23(7):631-9. doi: 10.1038/nsmb.3235.
117. Sui C-j, Zhou Y-m, Shen W-f, Dai B-h, Lu J-j, Zhang M-f, et al. Long noncoding RNA GIHCG promotes hepatocellular carcinoma progression through epigenetically regulating miR-200b/a/429. *J Mol Med (Berl)*. (2016) 94(11):1281-96. doi: 10.1007/s00109-016-1442-z.
118. Yuan SX, Wang J, Yang F, Tao QF, Zhang J, Wang LL, et al. Long noncoding RNA DANCR increases stemness features of hepatocellular carcinoma by derepression of CTNNB1. *Hepatology*. (2016) 63(2):499-511. doi: 10.1002/hep.27893.
119. Chen L, Yao H, Wang K, Liu X. Long Non-Coding RNA MALAT1 Regulates ZEB1 Expression by Sponging miR-143-3p and Promotes Hepatocellular Carcinoma Progression. *J Cell Biochem*. (2017) 118(12):4836-43. doi: 10.1002/jcb.26158.
120. Wang Y, Chen F, Zhao M, Yang Z, Li J, Zhang S, et al. The long noncoding RNA HULC promotes liver cancer by increasing the expression of the HMGA2 oncogene via sequestration of the microRNA-186. *J Biol Chem*. (2017) 292(37):15395-407. doi: 10.1074/jbc.M117.783738.
121. Zhuang LK, Yang YT, Ma X, Han B, Wang ZS, Zhao QY, et al. MicroRNA-92b promotes hepatocellular carcinoma progression by targeting Smad7 and is mediated by long non-coding RNA XIST. *Cell Death Dis*. (2016) 7(4):e2203. doi: 10.1038/cddis.2016.100.
122. Wang F, Yuan JH, Wang SB, Yang F, Yuan SX, Ye C, et al. Oncofetal long noncoding RNA PVT1 promotes proliferation and stem cell-like property of hepatocellular carcinoma cells by stabilizing NOP2. *Hepatology*. (2014) 60(4):1278-90. doi: 10.1002/hep.27239.
123. Ge Y, Yan X, Jin Y, Yang X, Yu X, Zhou L, et al. MiRNA-192 [corrected] and miRNA-204 Directly Suppress lncRNA HOTTIP and Interrupt GLS1-Mediated Glutaminolysis in

Hepatocellular Carcinoma. *PLoS Genet*. (2015) 11(12):e1005726. doi: 10.1371/journal.pgen.1005726.
124. Wang X, Sun W, Shen W, Xia M, Chen C, Xiang D, et al. Long non-coding RNA DILC regulates liver cancer stem cells via IL-6/STAT3 axis. *J Hepatol*. (2016) 64(6):1283-94. doi: 10.1016/j.jhep.2016.01.019.
125. Guo W, Liu S, Cheng Y, Lu L, Shi J, Xu G, et al. ICAM-1-Related Noncoding RNA in Cancer Stem Cells Maintains ICAM-1 Expression in Hepatocellular Carcinoma. *Clin Cancer Res*. (2016) 22(8):2041-50. doi: 10.1158/1078-0432.Ccr-14-3106.
126. Li T, Xie J, Shen C, Cheng D, Shi Y, Wu Z, et al. Amplification of Long Noncoding RNA ZFAS1 Promotes Metastasis in Hepatocellular Carcinoma. *Cancer Res*. (2015) 75(15):3181-91. doi: 10.1158/0008-5472.Can-14-3721.
127. Yuan SX, Yang F, Yang Y, Tao QF, Zhang J, Huang G, et al. Long noncoding RNA associated with microvascular invasion in hepatocellular carcinoma promotes angiogenesis and serves as a predictor for hepatocellular carcinoma patients' poor recurrence-free survival after hepatectomy. *Hepatology*. (2012) 56(6):2231-41. doi: 10.1002/hep.25895.
128. Klingenberg M, Groß M, Goyal A, Polycarpou-Schwarz M, Miersch T, Ernst AS, et al. The Long Noncoding RNA Cancer Susceptibility 9 and RNA Binding Protein Heterogeneous Nuclear Ribonucleoprotein L Form a Complex and Coregulate Genes Linked to AKT Signaling. *Hepatology*. (2018) 68(5):1817-32. doi: 10.1002/hep.30102.
129. Huang JF, Guo YJ, Zhao CX, Yuan SX, Wang Y, Tang GN, et al. Hepatitis B virus X protein (HBx)-related long noncoding RNA (lncRNA) down-regulated expression by HBx (Dreh) inhibits hepatocellular carcinoma metastasis by targeting the intermediate filament protein vimentin. *Hepatology*. (2013) 57(5):1882-92. doi: 10.1002/hep.26195.
130. Wu Y, Liu J, Zheng Y, You L, Kuang D, Liu T. Suppressed expression of long non-coding RNA HOTAIR inhibits proliferation and tumourigenicity of renal carcinoma cells. *Tumour Biol*. (2014) 35(12):11887-94. doi: 10.1007/s13277-014-2453-4.
131. Xiong J, Liu Y, Jiang L, Zeng Y, Tang W. High expression of long non-coding RNA lncRNA-ATB is correlated with metastases and promotes cell migration and invasion in renal cell carcinoma. *Jpn J Clin Oncol*. (2016) 46(4):378-84. doi: 10.1093/jjco/hyv214 %J Japanese Journal of Clinical Oncology.
132. Zhang HM, Yang FQ, Chen SJ, Che J, Zheng JH. Upregulation of long non-coding RNA MALAT1 correlates with tumor progression and poor prognosis in clear cell renal cell carcinoma. *Tumour Biol*. (2015) 36(4):2947-55. doi: 10.1007/s13277-014-2925-6.
133. Xiong J, Liu Y, Luo S, Jiang L, Zeng Y, Chen Z, et al. High expression of the long non-coding RNA HEIRCC promotes Renal Cell Carcinoma metastasis by inducing epithelial-mesenchymal transition. *Oncotarget*. (2017) 8(4):6555-63. doi: 10.18632/oncotarget.14149.
134. Posa I, Carvalho S, Tavares J, Grosso AR. A pan-cancer analysis of MYC-PVT1 reveals CNV-unmediated deregulation and poor prognosis in renal carcinoma. *Oncotarget*. (2016) 7(30):47033-41. doi: 10.18632/oncotarget.9487.
135. Yao J, Chen Y, Wang Y, Liu S, Yuan X, Pan F, et al. Decreased expression of a novel lncRNA CADM1-AS1 is associated with poor prognosis in patients with clear cell renal cell carcinomas. *Int J Clin Exp Pathol*. (2014) 7(6):2758-67.
136. Wang M, Huang T, Luo G, Huang C, Xiao XY, Wang L, et al. Long non-coding RNA MEG3 induces renal cell carcinoma cells apoptosis by activating the mitochondrial pathway. *J Huazhong Univ Sci Technolog Med Sci*. (2015) 35(4):541-5. doi: 10.1007/s11596-015-1467-5.

137. Liu Y, Qian J, Li X, Chen W, Xu A, Zhao K, et al. Long noncoding RNA BX357664 regulates cell proliferation and epithelial-to-mesenchymal transition via inhibition of TGF-β1/p38/HSP27 signaling in renal cell carcinoma. *Oncotarget*. (2016) 7(49):81410-22. doi: 10.18632/oncotarget.12937.
138. Cao Y, Xu R, Xu X, Zhou Y, Cui L, He X. Downregulation of lncRNA CASC2 by microRNA-21 increases the proliferation and migration of renal cell carcinoma cells. *Mol Med Rep*. (2016) 14(1):1019-25. doi: 10.3892/mmr.2016.5337.
139. Yue B, Qiu S, Zhao S, Liu C, Zhang D, Yu F, et al. LncRNA-ATB mediated E-cadherin repression promotes the progression of colon cancer and predicts poor prognosis. *J Gastroenterol Hepatol*. (2016) 31(3):595-603. doi: 10.1111/jgh.13206.
140. Wu K, Xu K, Liu K, Huang J, Chen J, Zhang J, et al. Long noncoding RNA BC200 regulates cell growth and invasion in colon cancer. *Int J Biochem Cell Biol*. (2018) 99:219-25. doi: 10.1016/j.biocel.2018.04.001.
141. Jing N, Huang T, Guo H, Yang J, Li M, Chen Z, et al. LncRNA CASC15 promotes colon cancer cell proliferation and metastasis by regulating the miR-4310/LGR5/Wnt/β-catenin signaling pathway. *Mol Med Rep*. (2018) 18(2):2269-76. doi: 10.3892/mmr.2018.9191.
142. He X, Tan X, Wang X, Jin H, Liu L, Ma L, et al. C-Myc-activated long noncoding RNA CCAT1 promotes colon cancer cell proliferation and invasion. *Tumour Biol*. (2014) 35(12):12181-8. doi: 10.1007/s13277-014-2526-4.
143. Yu Y, Nangia-Makker P, Farhana L, Majumdar APN. A novel mechanism of lncRNA and miRNA interaction: CCAT2 regulates miR-145 expression by suppressing its maturation process in colon cancer cells. *Mol Cancer*. (2017) 16(1):155. doi: 10.1186/s12943-017-0725-5.
144. Yue B, Liu C, Sun H, Liu M, Song C, Cui R, et al. A Positive Feed-Forward Loop between LncRNA-CYTOR and Wnt/β-Catenin Signaling Promotes Metastasis of Colon Cancer. *Mol Ther*. (2018) 26(5):1287-98. doi: 10.1016/j.ymthe.2018.02.024.
145. Zhuang ST, Cai YJ, Gao HC, Qiu JF, Zeng L, Zheng WJ. Study on the function and mechanism of long non-coding RNA DMTF1v4 in the occurrence of colon cancer. *Eur Rev Med Pharmacol Sci*. (2018) 22(12):3779-88. doi: 10.26355/eurrev_201806_15260.
146. Lao Y, Li Q, Li N, Liu H, Liu K, Jiang G, et al. Long noncoding RNA ENST00000455974 plays an oncogenic role through up-regulating JAG2 in human DNA mismatch repair-proficient colon cancer. *Biochem Biophys Res Commun*. (2019) 508(2):339-47. doi: 10.1016/j.bbrc.2018.11.088.
147. Wu K, Zhang N, Ma J, Huang J, Chen J, Wang L, et al. Long noncoding RNA FAL1 promotes proliferation and inhibits apoptosis of human colon cancer cells. *IUBMB Life*. (2018) 70(11):1093-100. doi: 10.1002/iub.1880.
148. Yang L, Cui J, Wang Y, Tan J. FAM83H-AS1 is upregulated and predicts poor prognosis in colon cancer. *Biomed Pharmacother*. (2019) 118:109342. doi: 10.1016/j.biopha.2019.109342.
149. Matsumura K, Kawasaki Y, Miyamoto M, Kamoshida Y, Nakamura J, Negishi L, et al. The novel G-quadruplex-containing long non-coding RNA GSEC antagonizes DHX36 and modulates colon cancer cell migration. *Oncogene*. (2017) 36(9):1191-9. doi: 10.1038/onc.2016.282.
150. Fang C, Qiu S, Sun F, Li W, Wang Z, Yue B, et al. Long non-coding RNA HNF1A-AS1 mediated repression of miR-34a/SIRT1/p53 feedback loop promotes the metastatic progression of colon cancer by functioning as a competing endogenous RNA. *Cancer Lett*. (2017) 410:50-62. doi: 10.1016/j.canlet.2017.09.012.

151. Tatangelo F, Di Mauro A, Scognamiglio G, Aquino G, Lettiero A, Delrio P, et al. Posterior HOX genes and HOTAIR expression in the proximal and distal colon cancer pathogenesis. *J Transl Med*. (2018) 16(1):350. doi: 10.1186/s12967-018-1725-y.
152. Li SP, Xu HX, Yu Y, He JD, Wang Z, Xu YJ, et al. LncRNA HULC enhances epithelial-mesenchymal transition to promote tumorigenesis and metastasis of hepatocellular carcinoma via the miR-200a-3p/ZEB1 signaling pathway. *Oncotarget*. (2016) 7(27):42431-46. doi: 10.18632/oncotarget.9883.
153. Chen S, Bu D, Ma Y, Zhu J, Chen G, Sun L, et al. H19 Overexpression Induces Resistance to 1,25(OH)2D3 by Targeting VDR Through miR-675-5p in Colon Cancer Cells. *Neoplasia*. (2017) 19(3):226-36. doi: 10.1016/j.neo.2016.10.007.
154. Guo K, Gong W, Wang Q, Gu G, Zheng T, Li Y, et al. LINC01106 drives colorectal cancer growth and stemness through a positive feedback loop to regulate the Gli family factors. *Cell Death Dis*. (2020) 11(10):869. doi: 10.1038/s41419-020-03026-3.
155. Kong M, Yu X, Zheng Q, Zhang S, Guo W. Oncogenic roles of LINC01234 in various forms of human cancer. *Biomed Pharmacother*. (2022) 154:113570. doi: 10.1016/j.biopha.2022.113570.
156. Yu X, Mi L, Dong J, Zou J. Long intergenic non-protein-coding RNA 1567 (LINC01567) acts as a "sponge" against microRNA-93 in regulating the proliferation and tumorigenesis of human colon cancer stem cells. *BMC Cancer*. (2017) 17(1):716. doi: 10.1186/s12885-017-3731-5.
157. Liu J, Zhan Y, Wang J, Wang J, Guo J, Kong D. Long noncoding RNA LINC01578 drives colon cancer metastasis through a positive feedback loop with the NF-κB/YY1 axis. *Mol Oncol*. (2020) 14(12):3211-33. doi: 10.1002/1878-0261.12819.
158. Tian J, Cui P, Li Y, Yao X, Wu X, Wang Z, et al. LINC02418 promotes colon cancer progression by suppressing apoptosis via interaction with miR-34b-5p/BCL2 axis. *Cancer Cell Int*. (2020) 20:460. doi: 10.1186/s12935-020-01530-2.
159. Wang L, Bu P, Ai Y, Srinivasan T, Chen HJ, Xiang K, et al. A long non-coding RNA targets microRNA miR-34a to regulate colon cancer stem cell asymmetric division. *Elife*. (2016) 5. doi: 10.7554/eLife.14620.
160. Wang JH, Lu TJ, Kung ML, Yang YF, Yeh CY, Tu YT, et al. The Long Noncoding RNA LOC441461 (STX17-AS1) Modulates Colorectal Cancer Cell Growth and Motility. *Cancers (Basel)*. (2020) 12(11). doi: 10.3390/cancers12113171.
161. Forrest ME, Saiakhova A, Beard L, Buchner DA, Scacheri PC, LaFramboise T, et al. Colon Cancer-Upregulated Long Non-Coding RNA lincDUSP Regulates Cell Cycle Genes and Potentiates Resistance to Apoptosis. *Sci Rep*. (2018) 8(1):7324. doi: 10.1038/s41598-018-25530-5.
162. Tian W, Du Y, Ma Y, Gu L, Zhou J, Deng D. MALAT1-miR663a negative feedback loop in colon cancer cell functions through direct miRNA-lncRNA binding. *Cell Death Dis*. (2018) 9(9):857. doi: 10.1038/s41419-018-0925-y.
163. Kawasaki Y, Komiya M, Matsumura K, Negishi L, Suda S, Okuno M, et al. MYU, a Target lncRNA for Wnt/c-Myc Signaling, Mediates Induction of CDK6 to Promote Cell Cycle Progression. *Cell Rep*. (2016) 16(10):2554-64. doi: 10.1016/j.celrep.2016.08.015.
164. Huang W, Su G, Huang X, Zou A, Wu J, Yang Y, et al. Long noncoding RNA PCAT6 inhibits colon cancer cell apoptosis by regulating anti-apoptotic protein ARC expression via EZH2. *Cell Cycle*. (2019) 18(1):69-83. doi: 10.1080/15384101.2018.1558872.

165. Yu X, Zhao J, He Y. Long non-coding RNA PVT1 functions as an oncogene in human colon cancer through miR-30d-5p/RUNX2 axis. *J buon*. (2018) 23(1):48-54.
166. Zhou P, Sun L, Liu D, Liu C, Sun L. Long Non-Coding RNA lincRNA-ROR Promotes the Progression of Colon Cancer and Holds Prognostic Value by Associating with miR-145. *Pathol Oncol Res*. (2016) 22(4):733-40. doi: 10.1007/s12253-016-0061-x.
167. Zhang XW, Li SL, Zhang D, Sun XL, Zhai HJ. RP11-619L19.2 promotes colon cancer development by regulating the miR-1271-5p/CD164 axis. *Oncol Rep*. (2020) 44(6):2419-28. doi: 10.3892/or.2020.7794.
168. Shi D, Liang L, Zheng H, Cai G, Li X, Xu Y, et al. Silencing of long non-coding RNA SBDSP1 suppresses tumor growth and invasion in colorectal cancer. *Biomed Pharmacother*. (2017) 85:355-61. doi: 10.1016/j.biopha.2016.11.036.
169. Yang H, Wang S, Kang YJ, Wang C, Xu Y, Zhang Y, et al. Long non-coding RNA SNHG1 predicts a poor prognosis and promotes colon cancer tumorigenesis. *Oncol Rep*. (2018) 40(1):261-71. doi: 10.3892/or.2018.6412.
170. Liu KL, Wu J, Li WK, Li NS, Li Q, Lao YQ. LncRNA SNHG7 is an Oncogenic Biomarker Interacting with MicroRNA-193b in Colon Carcinogenesis. *Clin Lab*. (2019) 65(11). doi: 10.7754/Clin.Lab.2019.190501.
171. Jiang H, Li T, Qu Y, Wang X, Li B, Song J, et al. Long non-coding RNA SNHG15 interacts with and stabilizes transcription factor Slug and promotes colon cancer progression. *Cancer Lett*. (2018) 425:78-87. doi: 10.1016/j.canlet.2018.03.038.
172. Liu J, Zhan Y, Wang J, Wang J, Guo J, Kong D. lncRNA-SNHG17 promotes colon adenocarcinoma progression and serves as a sponge for miR-375 to regulate CBX3 expression. *Am J Transl Res*. (2020) 12(9):5283-95.
173. Zhai HY, Sui MH, Yu X, Qu Z, Hu JC, Sun HQ, et al. Overexpression of Long Non-Coding RNA TUG1 Promotes Colon Cancer Progression. *Med Sci Monit*. (2016) 22:3281-7. doi: 10.12659/msm.897072.
174. Taniue K, Kurimoto A, Sugimasa H, Nasu E, Takeda Y, Iwasaki K, et al. Long noncoding RNA UPAT promotes colon tumorigenesis by inhibiting degradation of UHRF1. *Proc Natl Acad Sci U S A*. (2016) 113(5):1273-8. doi: 10.1073/pnas.1500992113.
175. Li D, Bao J, Yao J, Li J. lncRNA USP2-AS1 promotes colon cancer progression by modulating Hippo/YAP1 signaling. *Am J Transl Res*. (2020) 12(9):5670-82.
176. Sun N, Zhang G, Liu Y. Long non-coding RNA XIST sponges miR-34a to promotes colon cancer progression via Wnt/β-catenin signaling pathway. *Gene*. (2018) 665:141-8. doi: 10.1016/j.gene.2018.04.014.
177. Ni X, Ding Y, Yuan H, Shao J, Yan Y, Guo R, et al. Long non-coding RNA ZEB1-AS1 promotes colon adenocarcinoma malignant progression via miR-455-3p/PAK2 axis. *Cell Prolif*. (2020) 53(1):e12723. doi: 10.1111/cpr.12723.
178. Fang C, Zan J, Yue B, Liu C, He C, Yan D. Long non-coding ribonucleic acid zinc finger antisense 1 promotes the progression of colonic cancer by modulating ZEB1 expression. *J Gastroenterol Hepatol*. (2017) 32(6):1204-11. doi: 10.1111/jgh.13646.
179. Marín-Béjar O, Marchese FP, Athie A, Sánchez Y, González J, Segura V, et al. Pint lincRNA connects the p53 pathway with epigenetic silencing by the Polycomb repressive complex 2. *Genome Biol*. (2013) 14(9):R104. doi: 10.1186/gb-2013-14-9-r104.
180. Liu Q, Huang J, Zhou N, Zhang Z, Zhang A, Lu Z, et al. LncRNA loc285194 is a p53-regulated tumor suppressor. *Nucleic Acids Res*. (2013) 41(9):4976-87. doi: 10.1093/nar/gkt182.

181. Wang ZK, Yang L, Wu LL, Mao H, Zhou YH, Zhang PF, et al. Long non-coding RNA LINC00261 sensitizes human colon cancer cells to cisplatin therapy. *Braz J Med Biol Res*. (2017) 51(2):e6793. doi: 10.1590/1414-431x20176793.
182. Sun Y, Cao FL, Qu LL, Wang ZM, Liu XY. MEG3 promotes liver cancer by activating PI3K/AKT pathway through regulating AP1G1. *Eur Rev Med Pharmacol Sci*. (2019) 23(4):1459-67. doi: 10.26355/eurrev_201902_17103.
183. Somasundaram S, Forrest ME, Moinova H, Cohen A, Varadan V, LaFramboise T, et al. The DNMT1-associated lincRNA DACOR1 reprograms genome-wide DNA methylation in colon cancer. *Clin Epigenetics*. (2018) 10(1):127. doi: 10.1186/s13148-018-0555-3.
184. Wang L, Wei Z, Wu K, Dai W, Zhang C, Peng J, et al. Long noncoding RNA B3GALT5-AS1 suppresses colon cancer liver metastasis via repressing microRNA-203. *Aging*. (2018) 10(12):3662-82. doi: 10.18632/aging.101628.
185. Sun X, Xin S, Zhang Y, Jin L, Liu X, Zhang J, et al. Long non-coding RNA CASC11 interacts with YBX1 to promote prostate cancer progression by suppressing the p53 pathway. *Int J Oncol*. (2022) 61(3). doi: 10.3892/ijo.2022.5400.
186. Zhao H, Dong H, Wang P, Zhu H. Long non-coding RNA SNHG17 enhances the aggressiveness of C4-2 human prostate cancer cells in association with β-catenin signaling. *Oncol Lett*. (2021) 21(6):472. doi: 10.3892/ol.2021.12733.
187. Weng W, Liu C, Li G, Ruan Q, Li H, Lin N, et al. Long non--coding RNA SNHG16 functions as a tumor activator by sponging miR-373-3p to regulate the TGF-β-R2/SMAD pathway in prostate cancer. *Mol Med Rep*. (2021) 24(6). doi: 10.3892/mmr.2021.12483.
188. Luo ZF, Peng Y, Liu FH, Ma JS, Hu G, Lai SL, et al. Long noncoding RNA SNHG14 promotes malignancy of prostate cancer by regulating with miR-5590-3p/YY1 axis. *Eur Rev Med Pharmacol Sci*. (2020) 24(9):4697-709. doi: 10.26355/eurrev_202005_21158.
189. Zhang H, Liu Y, Li M, Peng G, Zhu T, Sun X. The Long Non-coding RNA SNHG12 Functions as a Competing Endogenous RNA to Modulate the Progression of Cerebral Ischemia/Reperfusion Injury. *Mol Neurobiol*. (2022) 59(2):1073-87. doi: 10.1007/s12035-021-02648-8.
190. Wu Z, Chen D, Wang K, Cao C, Xu X. Long Non-coding RNA SNHG12 Functions as a Competing Endogenous RNA to Regulate MDM4 Expression by Sponging miR-129-5p in Clear Cell Renal Cell Carcinoma. *Front Oncol*. (2019) 9:1260. doi: 10.3389/fonc.2019.01260.
191. Shi Z, Zhang H, Jie S, Yang X, Huang Q, Mao Y, et al. Long non-coding RNA SNHG8 promotes prostate cancer progression through repressing miR-384 and up-regulating HOXB7. *J Gene Med*. (2021) 23(3):e3309. doi: 10.1002/jgm.3309.
192. Liu J, Yuan JF, Wang YZ. METTL3-stabilized lncRNA SNHG7 accelerates glycolysis in prostate cancer via SRSF1/c-Myc axis. *Exp Cell Res*. (2022) 416(1):113149. doi: 10.1016/j.yexcr.2022.113149.
193. Wang X, Song Y, Shi Y, Yang D, Li J, Yin B. SNHG3 could promote prostate cancer progression through reducing methionine dependence of PCa cells. *Cell Mol Biol Lett*. (2022) 27(1):13. doi: 10.1186/s11658-022-00313-z.
194. Xiong H, Shen J, Chen Z, Yang J, Xie B, Jia Y, et al. H19/let-7/Lin28 ceRNA network mediates autophagy inhibiting epithelial-mesenchymal transition in breast cancer. *Int J Oncol*. (2020) 56(3):794-806. doi: 10.3892/ijo.2020.4967.
195. Ge Y, Liu L, Luo L, Fang Y, Ni T. MIR22HG Aggravates Oxygen-Glucose Deprivation and Reoxygenation-Induced Cardiomyocyte Injury through the miR-9-3p/SH2B3 Axis. *Cardiovasc Ther*. (2022) 2022:7332298. doi: 10.1155/2022/7332298.

196. Liang D, Tian C, Zhang X. lncRNA MNX1-AS1 promotes prostate cancer progression through regulating miR-2113/MDM2 axis. *Mol Med Rep*. (2022) 26(1). doi: 10.3892/mmr.2022.12747.
197. Xiong S, Song H. LncRNA CERS6-AS1 Is a Tumor Promoter in Cervical Cancer by Sponging miR-195-5p. *Ann Clin Lab Sci*. (2023) 53(1):30-41.
198. Wang YY, Chen C. lncRNA-DANCR Promotes Taxol Resistance of Prostate Cancer Cells through Modulating the miR-33b-5p-LDHA Axis. *Dis Markers*. (2022) 2022:9516774. doi: 10.1155/2022/9516774.
199. Mu X, Shen Z, Lin Y, Xiao J, Xia K, Xu C, et al. LncRNA-MALAT1 Regulates Cancer Glucose Metabolism in Prostate Cancer via MYBL2/mTOR Axis. *Oxid Med Cell Longev*. (2022) 2022:8693259. doi: 10.1155/2022/8693259.
200. Liu H, He X, Li T, Qu Y, Xu L, Hou Y, et al. PCGEM1 promotes proliferation, migration and invasion in prostate cancer by sponging miR-506 to upregulate TRIAP1. *BMC Urol*. (2022) 22(1):14. doi: 10.1186/s12894-022-00969-x.
201. Zhao W, Zhu X, Jin Q, Lin B, Ji R. The lncRNA NEAT1/miRNA-766-5p/E2F3 Regulatory Axis Promotes Prostate Cancer Progression. *J Oncol*. (2022) 2022:1866972. doi: 10.1155/2022/1866972.
202. Li P, Xu H, Yang L, Zhan M, Shi Y, Zhang C, et al. E2F transcription factor 2-activated DLEU2 contributes to prostate tumorigenesis by upregulating serum and glucocorticoid-induced protein kinase 1. *Cell Death Dis*. (2022) 13(1):77. doi: 10.1038/s41419-022-04525-1.
203. Ye C, Qin S, Guo F, Yang Y, Wang H, Zhang C, et al. LncRNA EIF3J-AS1 functions as an oncogene by regulating MAFG to promote prostate cancer progression. *J Cancer*. (2022) 13(1):146-52. doi: 10.7150/jca.60676.
204. Yang C, Shen S, Zheng X, Ye K, Ge H, Sun Y, et al. Long non-coding RNA LINC00337 induces autophagy and chemoresistance to cisplatin in esophageal squamous cell carcinoma cells via upregulation of TPX2 by recruiting E2F4. *Faseb j*. (2020) 34(5):6055-69. doi: 10.1096/fj.201900731RR.
205. Cheng Y, Xiong HY, Li YM, Zuo HR, Liu Y, Liao GL. LncRNA HOXA11-AS promotes cell growth by sponging miR-24-3p to regulate JPT1 in prostate cancer. *Eur Rev Med Pharmacol Sci*. (2021) 25(14):4668-77. doi: 10.26355/eurrev_202107_26377.
206. Pan X, Chen G, Hu W. lncRNA HLA Complex Group 18 (HCG18) Facilitated Cell Proliferation, Invasion, and Migration of Prostate Cancer Through Modulating miR-370-3p/DDX3X Axis. *Reprod Sci*. (2021) 28(12):3406-16. doi: 10.1007/s43032-021-00614-2.
207. Yu C, Fan Y, Zhang Y, Liu L, Guo G. LINC00893 inhibits the progression of prostate cancer through miR-3173-5p/SOCS3/JAK2/STAT3 pathway. *Cancer Cell Int*. (2022) 22(1):228. doi: 10.1186/s12935-022-02637-4.
208. Zhang W, Shi C, Xu Q, Chen X, Zhu H, Zheng B. Long non-coding RNA MIR22HG suppresses cell proliferation and promotes apoptosis in prostate cancer cells by sponging microRNA-9-3p. *Bioengineered*. (2022) 13(5):13108-17. doi: 10.1080/21655979.2022.2079244.
209. Wei X, Hou Y, Zhang Y, Zhang H, Sun Z, Meng X, et al. Long non-coding RNA MAGI2-AS3 inactivates STAT3 pathway to inhibit prostate cancer cell proliferation via acting as a microRNA-424-5p sponge. *J Cancer*. (2022) 13(1):343-53. doi: 10.7150/jca.60749.
210. Zhou Z, Wu X, Zhou Y, Yan W. Long non-coding RNA ADAMTS9-AS1 inhibits the progression of prostate cancer by modulating the miR-142-5p/CCND1 axis. *J Gene Med*. (2021) 23(5):e3331. doi: 10.1002/jgm.3331.

211. Ma X, Wang Z, Ren H, Bao X, Zhang Y, Wang B, et al. Long Non-Coding RNA GAS5 Suppresses Tumor Progression and Enhances the Radiosensitivity of Prostate Cancer Through the miR-320a/RAB21 Axis. *Cancer Manag Res*. (2020) 12:8833-45. doi: 10.2147/cmar.S244123.
212. Huo W, Qi F, Wang K. Long non-coding RNA FER1L4 inhibits prostate cancer progression via sponging miR-92a-3p and upregulation of FBXW7. *Cancer Cell Int*. (2020) 20:64. doi: 10.1186/s12935-020-1143-0.
213. Zhou J, Song Q, Liu X, Ye H, Wang Y, Zhang L, et al. lncRNA Erbb4-IR is downregulated in prostate carcinoma and predicts prognosis. *Oncol Lett*. (2020) 19(5):3425-30. doi: 10.3892/ol.2020.11464.
214. Leidal AM, Huang HH, Marsh T, Solvik T, Zhang D, Ye J, et al. The LC3-conjugation machinery specifies the loading of RNA-binding proteins into extracellular vesicles. *Nat Cell Biol*. (2020) 22(2):187-99. doi: 10.1038/s41556-019-0450-y.
215. Fan L, Li H, Zhang Y. LINC00908 negatively regulates microRNA-483-5p to increase TSPYL5 expression and inhibit the development of prostate cancer. *Cancer Cell Int*. (2020) 20:10. doi: 10.1186/s12935-019-1073-x.
216. Li B, Guo Z, Liang Q, Zhou H, Luo Y, He S, et al. lncRNA DGCR5 Up-Regulates TGF-β1, Increases Cancer Cell Stemness and Predicts Survival of Prostate Cancer Patients. *Cancer Manag Res*. (2019) 11:10657-63. doi: 10.2147/cmar.S231112.
217. Ghafouri-Fard S, Khoshbakht T, Hussen BM, Baniahmad A, Taheri M, Rashnoo F. A review on the role of PCA3 lncRNA in carcinogenesis with an especial focus on prostate cancer. *Pathol Res Pract*. (2022) 231:153800. doi: 10.1016/j.prp.2022.153800.
218. Li HJ, Sun XM, Li ZK, Yin QW, Pang H, Pan JJ, et al. LncRNA UCA1 Promotes Mitochondrial Function of Bladder Cancer via the MiR-195/ARL2 Signaling Pathway. *Cell Physiol Biochem*. (2017) 43(6):2548-61. doi: 10.1159/000484507.
219. Lv M, Zhong Z, Huang M, Tian Q, Jiang R, Chen J. lncRNA H19 regulates epithelial-mesenchymal transition and metastasis of bladder cancer by miR-29b-3p as competing endogenous RNA. *Biochim Biophys Acta Mol Cell Res*. (2017) 1864(10):1887-99. doi: 10.1016/j.bbamcr.2017.08.001.
220. Dai R, Zhou Y, Chen Z, Zou Z, Pan Z, Liu P, et al. Lnc-MUC20-9 binds to ROCK1 and functions as a tumor suppressor in bladder cancer. *J Cell Biochem*. (2020) 121(10):4214-25. doi: 10.1002/jcb.29626.
221. Liao C, Long Z, Zhang X, Cheng J, Qi F, Wu S, et al. LncARSR sponges miR-129-5p to promote proliferation and metastasis of bladder cancer cells through increasing SOX4 expression. *Int J Biol Sci*. (2020) 16(1):1-11. doi: 10.7150/ijbs.39461.
222. Chen Y, Peng Y, Xu Z, Ge B, Xiang X, Zhang T, et al. LncROR Promotes Bladder Cancer Cell Proliferation, Migration, and Epithelial-Mesenchymal Transition. *Cell Physiol Biochem*. (2017) 41(6):2399-410. doi: 10.1159/000475910.
223. Yu Y, Zou YF, Hong RQ, Chen WJ, Chen L, Chen WQ, et al. Long non-coding RNA SNHG16 decreased SMAD4 to induce gemcitabine resistance in pancreatic cancer via EZH2-mediated epigenetic modification. *Kaohsiung J Med Sci*. (2022) 38(10):981-91. doi: 10.1002/kjm2.12574.
224. Zhu X, Li Y, Zhao S, Zhao S. LSINCT5 activates Wnt/β-catenin signaling by interacting with NCYM to promote bladder cancer progression. *Biochem Biophys Res Commun*. (2018) 502(3):299-306. doi: 10.1016/j.bbrc.2018.05.076.

225. Zhao F, Lin T, He W, Han J, Zhu D, Hu K, et al. Knockdown of a novel lincRNA AATBC suppresses proliferation and induces apoptosis in bladder cancer. *Oncotarget*. (2015) 6(2):1064-78. doi: 10.18632/oncotarget.2833.
226. Lin MG, Hong YK, Zhang Y, Lin BB, He XJ. Mechanism of lncRNA DUXAP8 in promoting proliferation of bladder cancer cells by regulating PTEN. *Eur Rev Med Pharmacol Sci*. (2018) 22(11):3370-7. doi: 10.26355/eurrev_201806_15158.
227. Zhan Y, Chen Z, He S, Gong Y, He A, Li Y, et al. Long non-coding RNA SOX2OT promotes the stemness phenotype of bladder cancer cells by modulating SOX2. *Mol Cancer*. (2020) 19(1):25. doi: 10.1186/s12943-020-1143-7.
228. Chen JB, Zhu YW, Guo X, Yu C, Liu PH, Li C, et al. Microarray expression profiles analysis revealed lncRNA OXCT1-AS1 promoted bladder cancer cell aggressiveness via miR-455-5p/JAK1 signaling. *J Cell Physiol*. (2019) 234(8):13592-601. doi: 10.1002/jcp.28037.
229. Hu B, Shi G, Li Q, Li W, Zhou H. Long noncoding RNA XIST participates in bladder cancer by downregulating p53 via binding to TET1. *J Cell Biochem*. (2019) 120(4):6330-8. doi: 10.1002/jcb.27920.
230. Hu R, Zhong P, Xiong L, Duan L. Long Noncoding RNA Cancer Susceptibility Candidate 8 Suppresses the Proliferation of Bladder Cancer Cells via Regulating Glycolysis. *DNA Cell Biol*. (2017) 36(9):767-74. doi: 10.1089/dna.2017.3785.
231. Yuan S, Luan X, Han G, Guo K, Wang S, Zhang X. LINC01638 lncRNA mediates the postoperative distant recurrence of bladder cancer by upregulating ROCK2. *Oncol Lett*. (2019) 18(5):5392-8. doi: 10.3892/ol.2019.10924.
232. Chen C, He W, Huang J, Wang B, Li H, Cai Q, et al. LNMAT1 promotes lymphatic metastasis of bladder cancer via CCL2 dependent macrophage recruitment. *Nat Commun*. (2018) 9(1):3826. doi: 10.1038/s41467-018-06152-x.
233. Chen C, Luo Y, He W, Zhao Y, Kong Y, Liu H, et al. Exosomal long noncoding RNA LNMAT2 promotes lymphatic metastasis in bladder cancer. *J Clin Invest*. (2020) 130(1):404-21. doi: 10.1172/jci130892.
234. Chen J, Li Y, Li Z, Cao L. LncRNA MST1P2/miR-133b axis affects the chemoresistance of bladder cancer to cisplatin-based therapy via Sirt1/p53 signaling. *J Biochem Mol Toxicol*. (2020) 34(4):e22452. doi: 10.1002/jbt.22452.
235. Qiu F, Zhang MR, Zhou Z, Pu JX, Zhao XJ. lncRNA MIR503HG functioned as a tumor suppressor and inhibited cell proliferation, metastasis and epithelial-mesenchymal transition in bladder cancer. *J Cell Biochem*. (2019) 120(6):10821-9. doi: 10.1002/jcb.28373.
236. Shan G, Tang T, Xia Y, Qian HJ. MEG3 interacted with miR-494 to repress bladder cancer progression through targeting PTEN. *J Cell Physiol*. (2020) 235(2):1120-8. doi: 10.1002/jcp.29025.
237. Xie H, Huang H, Huang W, Xie Z, Yang Y, Wang F. LncRNA miR143HG suppresses bladder cancer development through inactivating Wnt/β-catenin pathway by modulating miR-1275/AXIN2 axis. *J Cell Physiol*. (2019) 234(7):11156-64. doi: 10.1002/jcp.27764.
238. Pei Z, Du X, Song Y, Fan L, Li F, Gao Y, et al. Down-regulation of lncRNA CASC2 promotes cell proliferation and metastasis of bladder cancer by activation of the Wnt/β-catenin signaling pathway. *Oncotarget*. (2017) 8(11):18145-53. doi: 10.18632/oncotarget.15210.
239. Zheng R, Du M, Wang X, Xu W, Liang J, Wang W, et al. Exosome-transmitted long non-coding RNA PTENP1 suppresses bladder cancer progression. *Mol Cancer*. (2018) 17(1):143. doi: 10.1186/s12943-018-0880-3.

240. Wang YY, Wu ZY, Wang GC, Liu K, Niu XB, Gu S, et al. LINC00312 inhibits the migration and invasion of bladder cancer cells by targeting miR-197-3p. *Tumour Biol*. (2016) 37(11):14553-63. doi: 10.1007/s13277-016-5303-8.
241. Li Z, Hong S, Liu Z. LncRNA LINC00641 predicts prognosis and inhibits bladder cancer progression through miR-197-3p/KLF10/PTEN/PI3K/AKT cascade. *Biochem Biophys Res Commun*. (2018) 503(3):1825-9. doi: 10.1016/j.bbrc.2018.07.120.
242. Zhu Y, Dai B, Zhang H, Shi G, Shen Y, Ye D. Long non-coding RNA LOC572558 inhibits bladder cancer cell proliferation and tumor growth by regulating the AKT-MDM2-p53 signaling axis. *Cancer Lett*. (2016) 380(2):369-74. doi: 10.1016/j.canlet.2016.04.030.
243. Zhang M, Xu Y, Yin S, Qiu F. YY1-induced long non-coding RNA PSMA3 antisense RNA 1 functions as a competing endogenous RNA for microRNA 214-5p to expedite the viability and restrict the apoptosis of bladder cancer cells via regulating programmed cell death-ligand 1. *Bioengineered*. (2021) 12(2):9150-61. doi: 10.1080/21655979.2021.1994907.
244. Shan QL, Chen NN, Meng GZ, Qu F. Overexpression of lncRNA MT1JP Mediates Apoptosis and Migration of Hepatocellular Carcinoma Cells by Regulating miR-24-3p. *Cancer Manag Res*. (2020) 12:4715-24. doi: 10.2147/cmar.S249582.
245. Fang C, He W, Xu T, Dai J, Xu L, Sun F. Upregulation of lncRNA DGCR5 correlates with better prognosis and inhibits bladder cancer progression via transcriptionally facilitating P21 expression. *J Cell Physiol*. (2019) 234(5):6254-62. doi: 10.1002/jcp.27356.
246. Chen D, Guo Y, Chen Y, Guo Q, Chen J, Li Y, et al. LncRNA growth arrest-specific transcript 5 targets miR-21 gene and regulates bladder cancer cell proliferation and apoptosis through PTEN. *Cancer Med*. (2020) 9(8):2846-58. doi: 10.1002/cam4.2664.
247. Wei X, Yang X, Wang B, Yang Y, Fang Z, Yi C, et al. LncRNA MBNL1-AS1 represses cell proliferation and enhances cell apoptosis via targeting miR-135a-5p/PHLPP2/FOXO1 axis in bladder cancer. *Cancer Med*. (2020) 9(2):724-36. doi: 10.1002/cam4.2684.
248. Tuo Z, Zhang J, Xue W. LncRNA TP73-AS1 predicts the prognosis of bladder cancer patients and functions as a suppressor for bladder cancer by EMT pathway. *Biochem Biophys Res Commun*. (2018) 499(4):875-81. doi: 10.1016/j.bbrc.2018.04.010.
249. Chen X, Xie R, Gu P, Huang M, Han J, Dong W, et al. Long Noncoding RNA LBCS Inhibits Self-Renewal and Chemoresistance of Bladder Cancer Stem Cells through Epigenetic Silencing of SOX2. *Clin Cancer Res*. (2019) 25(4):1389-403. doi: 10.1158/1078-0432.Ccr-18-1656.
250. Liu M, Jia J, Wang X, Liu Y, Wang C, Fan R. Long non-coding RNA HOTAIR promotes cervical cancer progression through regulating BCL2 via targeting miR-143-3p. *Cancer Biol Ther*. (2018) 19(5):391-9. doi: 10.1080/15384047.2018.1423921.
251. Roychowdhury A, Samadder S, Das P, Mazumder DI, Chatterjee A, Addya S, et al. Deregulation of H19 is associated with cervical carcinoma. *Genomics*. (2020) 112(1):961-70. doi: 10.1016/j.ygeno.2019.06.012.
252. Ye D, Shen Z, Zhou S. Function of microRNA-145 and mechanisms underlying its role in malignant tumor diagnosis and treatment. *Cancer Manag Res*. (2019) 11:969-79. doi: 10.2147/cmar.S191696.
253. Aalijahan H, Ghorbian S. Long non-coding RNAs and cervical cancer. *Exp Mol Pathol*. (2019) 106:7-16. doi: 10.1016/j.yexmp.2018.11.010.
254. Wang Z, Sha HH, Li HJ. Functions and mechanisms of miR-186 in human cancer. *Biomed Pharmacother*. (2019) 119:109428. doi: 10.1016/j.biopha.2019.109428.

255. Zhu M, Li X, Zhu S, Li P, Min L, Zhang S. Long non-coding RNA BLACAT1, a novel promising biomarker and regulator of human cancers. *Biomed Pharmacother*. (2020) 132:110808. doi: 10.1016/j.biopha.2020.110808.
256. Guo Y, Luo S. miR-140-5p inhibits cervical cancer cell phenotypes via downregulating FEN1 to halt the cell cycle. *Mol Med Rep*. (2020) 22(6):4919-30. doi: 10.3892/mmr.2020.11552.
257. Fan MJ, Zou YH, He PJ, Zhang S, Sun XM, Li CZ. Long non-coding RNA SPRY4-IT1 promotes epithelial-mesenchymal transition of cervical cancer by regulating the miR-101-3p/ZEB1 axis. *Biosci Rep*. (2019) 39(6). doi: 10.1042/bsr20181339.
258. Xie F, Xie G, Sun Q. Long Noncoding RNA DLX6-AS1 Promotes the Progression in Cervical Cancer by Targeting miR-16-5p/ARPP19 Axis. *Cancer Biother Radiopharm*. (2020) 35(2):129-36. doi: 10.1089/cbr.2019.2960.
259. Wang M, Wang X, Liu W. MicroRNA-130a-3p promotes the proliferation and inhibits the apoptosis of cervical cancer cells via negative regulation of RUNX3. *Mol Med Rep*. (2020) 22(4):2990-3000. doi: 10.3892/mmr.2020.11368.
260. Bai X, Wang W, Zhao P, Wen J, Guo X, Shen T, et al. LncRNA CRNDE acts as an oncogene in cervical cancer through sponging miR-183 to regulate CCNB1 expression. *Carcinogenesis*. (2020) 41(1):111-21. doi: 10.1093/carcin/bgz166.
261. Chang QQ, Chen CY, Chen Z, Chang S. LncRNA PVT1 promotes proliferation and invasion through enhancing Smad3 expression by sponging miR-140-5p in cervical cancer. *Radiol Oncol*. (2019) 53(4):443-52. doi: 10.2478/raon-2019-0048.
262. Zhang J, Gao Y. Long non-coding RNA MEG3 inhibits cervical cancer cell growth by promoting degradation of P-STAT3 protein via ubiquitination. *Cancer Cell Int*. (2019) 19:175. doi: 10.1186/s12935-019-0893-z.
263. Yang X, Xie Z, Lei X, Gan R. Long non-coding RNA GAS5 in human cancer. *Oncol Lett*. (2020) 20(3):2587-94. doi: 10.3892/ol.2020.11809.
264. Yuan D, Qian H, Guo T, Ye J, Jin C, Liu X, et al. LncRNA-ATB Promotes the Tumorigenesis of Ovarian Cancer via Targeting miR-204-3p. *Onco Targets Ther*. (2020) 13:573-83. doi: 10.2147/ott.S230552.
265. Hu X, Li Y, Kong D, Hu L, Liu D, Wu J. Long noncoding RNA CASC9 promotes LIN7A expression via miR-758-3p to facilitate the malignancy of ovarian cancer. *J Cell Physiol*. (2019) 234(7):10800-8. doi: 10.1002/jcp.27903.
266. Cao Y, Shi H, Ren F, Jia Y, Zhang R. Long non-coding RNA CCAT1 promotes metastasis and poor prognosis in epithelial ovarian cancer. *Exp Cell Res*. (2017) 359(1):185-94. doi: 10.1016/j.yexcr.2017.07.030.
267. Hua F, Li CH, Chen XG, Liu XP. Long Noncoding RNA CCAT2 Knockdown Suppresses Tumorous Progression by Sponging miR-424 in Epithelial Ovarian Cancer. *Oncol Res*. (2018) 26(2):241-7. doi: 10.3727/096504017x14953948675412.
268. Wang Y, Huang Y, Liu H, Su D, Luo F, Zhou F. Long noncoding RNA CDKN2B-AS1 interacts with miR-411-3p to regulate ovarian cancer in vitro and in vivo through HIF-1a/VEGF/P38 pathway. *Biochem Biophys Res Commun*. (2019) 514(1):44-50. doi: 10.1016/j.bbrc.2019.03.141.
269. Lin X, Yang F, Qi X, Li Q, Wang D, Yi T, et al. LncRNA DANCR promotes tumor growth and angiogenesis in ovarian cancer through direct targeting of miR-145. *Mol Carcinog*. (2019) 58(12):2286-96. doi: 10.1002/mc.23117.

270. Wang LL, Sun KX, Wu DD, Xiu YL, Chen X, Chen S, et al. DLEU1 contributes to ovarian carcinoma tumourigenesis and development by interacting with miR-490-3p and altering CDK1 expression. *J Cell Mol Med*. (2017) 21(11):3055-65. doi: 10.1111/jcmm.13217.
271. Kong L, Zhang C. LncRNA DLX6-AS1 aggravates the development of ovarian cancer via modulating FHL2 by sponging miR-195-5p. *Cancer Cell Int*. (2020) 20(1):370. doi: 10.1186/s12935-020-01452-z.
272. Yan H, Silva MA, Li H, Zhu L, Li P, Li X, et al. Long noncoding RNA DQ786243 interacts with miR-506 and promotes progression of ovarian cancer through targeting cAMP responsive element binding protein 1. *J Cell Biochem*. (2018) 119(12):9764-80. doi: 10.1002/jcb.27295.
273. Chen ZH, Cui MY, Zhang HM. EMX2OS Delays Wilms'Tumor Progression via Targeting miR-654-3p. *Ann Clin Lab Sci*. (2022) 52(1):12-20.
274. Yan H, Li H, Silva MA, Guan Y, Yang L, Zhu L, et al. LncRNA FLVCR1-AS1 mediates miR-513/YAP1 signaling to promote cell progression, migration, invasion and EMT process in ovarian cancer. *Journal of Experimental & Clinical Cancer Research*. (2019) 38(1):356. doi: 10.1186/s13046-019-1356-z.
275. Yao N, Yu L, Zhu B, Gan HY, Guo BQ. LncRNA GIHCG promotes development of ovarian cancer by regulating microRNA-429. *Eur Rev Med Pharmacol Sci*. (2018) 22(23):8127-34. doi: 10.26355/eurrev_201812_16504.
276. Li J, Huang Y, Deng X, Luo M, Wang X, Hu H, et al. Long noncoding RNA H19 promotes transforming growth factor-β-induced epithelial-mesenchymal transition by acting as a competing endogenous RNA of miR-370-3p in ovarian cancer cells. *Onco Targets Ther*. (2018) 11:427-40. doi: 10.2147/ott.S149908.
277. Yang M, Zhai Z, Zhang Y, Wang Y. Clinical significance and oncogene function of long noncoding RNA HAGLROS overexpression in ovarian cancer. *Arch Gynecol Obstet*. (2019) 300(3):703-10. doi: 10.1007/s00404-019-05218-5.
278. Tong L, Wang Y, Ao Y, Sun X. CREB1 induced lncRNA HAS2-AS1 promotes epithelial ovarian cancer proliferation and invasion via the miR-466/RUNX2 axis. *Biomed Pharmacother*. (2019) 115:108891. doi: 10.1016/j.biopha.2019.108891.
279. Gao Y, Meng H, Liu S, Hu J, Zhang Y, Jiao T, et al. LncRNA-HOST2 regulates cell biological behaviors in epithelial ovarian cancer through a mechanism involving microRNA let-7b. *Hum Mol Genet*. (2015) 24(3):841-52. doi: 10.1093/hmg/ddu502.
280. Dong L, Hui L. HOTAIR Promotes Proliferation, Migration, and Invasion of Ovarian Cancer SKOV3 Cells Through Regulating PIK3R3. *Med Sci Monit*. (2016) 22:325-31. doi: 10.12659/msm.894913.
281. Wang Y, Zhang W, Wang Y, Wang S. HOXD-AS1 promotes cell proliferation, migration and invasion through miR-608/FZD4 axis in ovarian cancer. *Am J Cancer Res*. (2018) 8(1):170-82.
282. Ma T, Chen H, Wang P, Yang N, Bao J. Downregulation of lncRNA ZEB1-AS1 Represses Cell Proliferation, Migration, and Invasion Through Mediating PI3K/AKT/mTOR Signaling by miR-342-3p/CUL4B Axis in Prostate Cancer. *Cancer Biother Radiopharm*. (2020) 35(9):661-72. doi: 10.1089/cbr.2019.3123.
283. Liu H, Chen R, Kang F, Lai H, Wang Y. KCNQ1OT1 promotes ovarian cancer progression via modulating MIR-142-5p/CAPN10 axis. *Mol Genet Genomic Med*. (2020) 8(2):e1077. doi: 10.1002/mgg3.1077.

284. Zhang Y, Ruan F. LncRNA LEF1-AS1 Promotes Ovarian Cancer Development Through Interacting with miR-1285-3p. *Cancer Manag Res*. (2020) 12:687-94. doi: 10.2147/cmar.S227652.
285. Chen P, Fang X, Xia B, Zhao Y, Li Q, Wu X. Long noncoding RNA LINC00152 promotes cell proliferation through competitively binding endogenous miR-125b with MCL-1 by regulating mitochondrial apoptosis pathways in ovarian cancer. *Cancer Med*. (2018) 7(9):4530-41. doi: 10.1002/cam4.1547.
286. Xu M, Zhou K, Wu Y, Wang L, Lu S. Linc00161 regulated the drug resistance of ovarian cancer by sponging microRNA-128 and modulating MAPK1. *Mol Carcinog*. (2019) 58(4):577-87. doi: 10.1002/mc.22952.
287. Du W, Feng Z, Sun Q. LncRNA LINC00319 accelerates ovarian cancer progression through miR-423-5p/NACC1 pathway. *Biochem Biophys Res Commun*. (2018) 507(1-4):198-202. doi: 10.1016/j.bbrc.2018.11.006.
288. Pan L, Meng Q, Li H, Liang K, Li B. LINC00339 promotes cell proliferation, migration, and invasion of ovarian cancer cells via miR-148a-3p/ROCK1 axes. *Biomed Pharmacother*. (2019) 120:109423. doi: 10.1016/j.biopha.2019.109423.
289. Zhang C, Li Y, Zhao W, Liu G, Yang Q. Circ-PGAM1 promotes malignant progression of epithelial ovarian cancer through regulation of the miR-542-3p/CDC5L/PEAK1 pathway. *Cancer Med*. (2020) 9(10):3500-21. doi: 10.1002/cam4.2929.
290. Wang K, Zhu G, Bao S, Chen S. Long Non-Coding RNA LINC00511 Mediates the Effects of ESR1 on Proliferation and Invasion of Ovarian Cancer Through miR-424-5p and miR-370-5p. *Cancer Manag Res*. (2019) 11:10807-19. doi: 10.2147/cmar.S232140.
291. Wu Y, Cong L, Chen W, Wang X, Qiu F. lncRNA LINC00963 downregulation regulates colorectal cancer tumorigenesis and progression via the miR-10b/FGF13 axis. *Mol Med Rep*. (2021) 23(3). doi: 10.3892/mmr.2021.11850.
292. Shi C, Wang M. LINC01118 Modulates Paclitaxel Resistance of Epithelial Ovarian Cancer by Regulating miR-134/ABCC1. *Med Sci Monit*. (2018) 24:8831-9. doi: 10.12659/msm.910932.
293. Yu H, Xu Y, Zhang D, Liu G. Long noncoding RNA LUCAT1 promotes malignancy of ovarian cancer through regulation of miR-612/HOXA13 pathway. *Biochem Biophys Res Commun*. (2018) 503(3):2095-100. doi: 10.1016/j.bbrc.2018.07.165.
294. Lei R, Xue M, Zhang L, Lin Z. Long noncoding RNA MALAT1-regulated microRNA 506 modulates ovarian cancer growth by targeting iASPP. *Onco Targets Ther*. (2017) 10:35-46. doi: 10.2147/ott.S112686.
295. Zheng C, Chu M, Chen Q, Chen C, Wang ZW, Chen X. The role of lncRNA OIP5-AS1 in cancer development and progression. *Apoptosis*. (2022) 27(5-6):311-21. doi: 10.1007/s10495-022-01722-3.
296. Zhu L, Wang A, Gao M, Duan X, Li Z. LncRNA MIR4435-2HG triggers ovarian cancer progression by regulating miR-128-3p/CKD14 axis. *Cancer Cell Int*. (2020) 20:145. doi: 10.1186/s12935-020-01227-6.
297. Chang H, Li B, Zhang X, Meng X. NCK1-AS1 promotes NCK1 expression to facilitate tumorigenesis and chemo-resistance in ovarian cancer. *Biochem Biophys Res Commun*. (2020) 522(2):292-9. doi: 10.1016/j.bbrc.2019.11.014.
298. Chai Y, Liu J, Zhang Z, Liu L. HuR-regulated lncRNA NEAT1 stability in tumorigenesis and progression of ovarian cancer. *Cancer Med*. (2016) 5(7):1588-98. doi: 10.1002/cam4.710.

299. Liu Y, Wang Y, Fu X, Lu Z. Long non-coding RNA NEAT1 promoted ovarian cancer cells' metastasis through regulation of miR-382-3p/ROCK1 axial. *Cancer Sci*. (2018) 109(7):2188-98. doi: 10.1111/cas.13647.
300. Yong W, Yu D, Jun Z, Yachen D, Weiwei W, Midie X, et al. Long noncoding RNA NEAT1, regulated by LIN28B, promotes cell proliferation and migration through sponging miR-506 in high-grade serous ovarian cancer. *Cell Death Dis*. (2018) 9(9):861. doi: 10.1038/s41419-018-0908-z.
301. Xu C, Zhu LX, Sun DM, Yao H, Han DX. Regulatory mechanism of lncRNA NORAD on proliferation and invasion of ovarian cancer cells through miR-199a-3p. *Eur Rev Med Pharmacol Sci*. (2020) 24(4):1672-81. doi: 10.26355/eurrev_202002_20341.
302. Tao F, Tian X, Lu M, Zhang Z. A novel lncRNA, Lnc-OC1, promotes ovarian cancer cell proliferation and migration by sponging miR-34a and miR-34c. *J Genet Genomics*. (2018) 45(3):137-45. doi: 10.1016/j.jgg.2018.03.001.
303. Guo L, Chen J, Liu D, Liu L. OIP5-AS1/miR-137/ZNF217 Axis Promotes Malignant Behaviors in Epithelial Ovarian Cancer. *Cancer Manag Res*. (2020) 12:6707-17. doi: 10.2147/cmar.S237726.
304. Gu LP, Jin S, Xu RC, Zhang J, Geng YC, Shao XY, et al. Long non-coding RNA PCAT-1 promotes tumor progression by inhibiting miR-129-5p in human ovarian cancer. *Arch Med Sci*. (2019) 15(2):513-21. doi: 10.5114/aoms.2018.75534.
305. Xiong T, Wang Y, Zhang Y, Yuan J, Zhu C, Jiang W. lncRNA AC005224.4/miR-140-3p/SNAI2 regulating axis facilitates the invasion and metastasis of ovarian cancer through epithelial-mesenchymal transition. *Chin Med J (Engl)*. (2023) 136(9):1098-110. doi: 10.1097/cm9.0000000000002201.
306. Liang H, Yu T, Han Y, Jiang H, Wang C, You T, et al. LncRNA PTAR promotes EMT and invasion-metastasis in serous ovarian cancer by competitively binding miR-101-3p to regulate ZEB1 expression. *Mol Cancer*. (2018) 17(1):119. doi: 10.1186/s12943-018-0870-5.
307. Liang H, Yu M, Yang R, Zhang L, Zhang L, Zhu D, et al. A PTAL-miR-101-FN1 Axis Promotes EMT and Invasion-Metastasis in Serous Ovarian Cancer. *Mol Ther Oncolytics*. (2020) 16:53-62. doi: 10.1016/j.omto.2019.12.002.
308. Yang Q, Yu Y, Sun Z, Pan Y. Long non-coding RNA PVT1 promotes cell proliferation and invasion through regulating miR-133a in ovarian cancer. *Biomed Pharmacother*. (2018) 106:61-7. doi: 10.1016/j.biopha.2018.06.112.
309. Zhao L, Liu T, Zhang X, Zuo D, Liu C. lncRNA RHPN1-AS1 Promotes Ovarian Cancer Growth and Invasiveness Through Inhibiting miR-1299. *Onco Targets Ther*. (2020) 13:5337-44. doi: 10.2147/ott.S248050.
310. Li J, Zhang S, Wu L, Pei M. Interaction between LncRNA-ROR and miR-145 contributes to epithelial-mesenchymal transition of ovarian cancer cells. *Gen Physiol Biophys*. (2019) 38(6):461-71. doi: 10.4149/gpb_2019028.
311. Song R, Liu Z, Lu L, Liu F, Zhang B. Long Noncoding RNA SCAMP1 Targets miR-137/CXCL12 Axis to Boost Cell Invasion and Angiogenesis in Ovarian Cancer. *DNA Cell Biol*. (2020) 39(6):1041-50. doi: 10.1089/dna.2019.5312.
312. Sun D, Fan XH. LncRNA SNHG12 accelerates the progression of ovarian cancer via absorbing miRNA-129 to upregulate SOX4. *Eur Rev Med Pharmacol Sci*. (2019) 23(6):2345-52. doi: 10.26355/eurrev_201903_17378.

313. Chen S, Wang LL, Sun KX, Xiu YL, Zong ZH, Chen X, et al. The role of the long non-coding RNA TDRG1 in epithelial ovarian carcinoma tumorigenesis and progression through miR-93/RhoC pathway. *Mol Carcinog*. (2018) 57(2):225-34. doi: 10.1002/mc.22749.
314. Li H, Zhou Y, Cheng H, Tian J, Yang S. Roles of a TMPO-AS1/microRNA-200c/TMEFF2 ceRNA network in the malignant behaviors and 5-FU resistance of ovarian cancer cells. *Exp Mol Pathol*. (2020) 115:104481. doi: 10.1016/j.yexmp.2020.104481.
315. Miao S, Wang J, Xuan L, Liu X. LncRNA TTN-AS1 acts as sponge for miR-15b-5p to regulate FBXW7 expression in ovarian cancer. *Biofactors*. (2020) 46(4):600-7. doi: 10.1002/biof.1622.
316. Liu M, Zhang H, Li Y, Wang S. Noncoding RNAs Interplay in Ovarian Cancer Therapy and Drug Resistance. *Cancer Biother Radiopharm*. (2022) 37(3):186-98. doi: 10.1089/cbr.2021.0339.
317. Wang J, Ye C, Liu J, Hu Y. UCA1 confers paclitaxel resistance to ovarian cancer through miR-129/ABCB1 axis. *Biochem Biophys Res Commun*. (2018) 501(4):1034-40. doi: 10.1016/j.bbrc.2018.05.104.
318. Xia B, Hou Y, Chen H, Yang S, Liu T, Lin M, et al. Long non-coding RNA ZFAS1 interacts with miR-150-5p to regulate Sp1 expression and ovarian cancer cell malignancy. *Oncotarget*. (2017) 8(12):19534-46. doi: 10.18632/oncotarget.14663.
319. Wang A, Jin C, Li H, Qin Q, Li L. LncRNA ADAMTS9-AS2 regulates ovarian cancer progression by targeting miR-182-5p/FOXF2 signaling pathway. *Int J Biol Macromol*. (2018) 120(Pt B):1705-13. doi: 10.1016/j.ijbiomac.2018.09.179.
320. Sun T, Yang P, Gao Y. Long non-coding RNA EPB41L4A-AS2 suppresses progression of ovarian cancer by sequestering microRNA-103a to upregulate transcription factor RUNX1T1. *Exp Physiol*. (2020) 105(1):75-87. doi: 10.1113/ep087847.
321. Ma N, Li S, Zhang Q, Wang H, Qin H, Wang S. Long non-coding RNA GAS5 inhibits ovarian cancer cell proliferation via the control of microRNA-21 and SPRY2 expression. *Exp Ther Med*. (2018) 16(1):73-82. doi: 10.3892/etm.2018.6188.
322. Gokulnath P, de Cristofaro T, Manipur I, Di Palma T, Soriano AA, Guarracino MR, et al. Long Non-Coding RNA HAND2-AS1 Acts as a Tumor Suppressor in High-Grade Serous Ovarian Carcinoma. *Int J Mol Sci*. (2020) 21(11):4059.
323. Chao H, Zhang M, Hou H, Zhang Z, Li N. HOTAIRM1 suppresses cell proliferation and invasion in ovarian cancer through facilitating ARHGAP24 expression by sponging miR-106a-5p. *Life Sci*. (2020) 243:117296. doi: 10.1016/j.lfs.2020.117296.
324. Zhang W, Fei J, Yu S, Shen J, Zhu X, Sadhukhan A, et al. LINC01088 inhibits tumorigenesis of ovarian epithelial cells by targeting miR-24-1-5p. *Sci Rep*. (2018) 8(1):2876. doi: 10.1038/s41598-018-21164-9.
325. Guo J, Pan H. Long Noncoding RNA LINC01125 Enhances Cisplatin Sensitivity of Ovarian Cancer via miR-1972. *Med Sci Monit*. (2019) 25:9844-54. doi: 10.12659/msm.916820.
326. Liu M, Shen C, Wang C. Long Noncoding RNA LINC01133 Confers Tumor-Suppressive Functions in Ovarian Cancer by Regulating Leucine-Rich Repeat Kinase 2 as an miR-205 Sponge. *Am J Pathol*. (2019) 189(11):2323-39. doi: 10.1016/j.ajpath.2019.07.020.
327. Chang H, Zhang X, Li B, Meng X. MAGI2-AS3 suppresses MYC signaling to inhibit cell proliferation and migration in ovarian cancer through targeting miR-525-5p/MXD1 axis. *Cancer Med*. (2020) 9(17):6377-86. doi: 10.1002/cam4.3126.
328. Xu H, Ding Y, Yang X. Overexpression of Long Noncoding RNA H19 Downregulates miR-140-5p and Activates PI3K/AKT Signaling Pathway to Promote Invasion, Migration and

Epithelial-Mesenchymal Transition of Ovarian Cancer Cells. *Biomed Res Int*. (2021) 2021:6619730. doi: 10.1155/2021/6619730.
329. Chen X, Wu W, Cao X, Zhao X, Li W, Deng C, et al. lncRNA mortal obligate RNA transcript was downregulated in ovarian carcinoma and inhibits cancer cell proliferation by downregulating miRNA-21. *J Cell Biochem*. (2019) 120(7):11949-54. doi: 10.1002/jcb.28478.
330. Li W, Ma S, Bai X, Pan W, Ai L, Tan W. Long noncoding RNA WDFY3-AS2 suppresses tumor progression by acting as a competing endogenous RNA of microRNA-18a in ovarian cancer. *J Cell Physiol*. (2020) 235(2):1141-54. doi: 10.1002/jcp.29028.
331. Agiannitopoulos K, Samara P, Papadopoulou M, Efthymiadou A, Papadopoulou E, Tsaousis GN, et al. miRNA polymorphisms and risk of premature coronary artery disease. *Hellenic J Cardiol*. (2021) 62(4):278-84. doi: 10.1016/j.hjc.2020.01.005.
332. Wang G, Pan J, Zhang L, Wei Y, Wang C. Long non-coding RNA CRNDE sponges miR-384 to promote proliferation and metastasis of pancreatic cancer cells through upregulating IRS1. *Cell Prolif*. (2017) 50(6). doi: 10.1111/cpr.12389.
333. Deng S, Wang J, Zhang L, Li J, Jin Y. LncRNA HOTAIR Promotes Cancer Stem-Like Cells Properties by Sponging miR-34a to Activate the JAK2/STAT3 Pathway in Pancreatic Ductal Adenocarcinoma. *Onco Targets Ther*. (2021) 14:1883-93. doi: 10.2147/ott.S286666.
334. Cheng Y, Jutooru I, Chadalapaka G, Corton JC, Safe S. The long non-coding RNA HOTTIP enhances pancreatic cancer cell proliferation, survival and migration. *Oncotarget*. (2015) 6(13):10840-52. doi: 10.18632/oncotarget.3450.
335. Lu J, Wei JH, Feng ZH, Chen ZH, Wang YQ, Huang Y, et al. miR-106b-5p promotes renal cell carcinoma aggressiveness and stem-cell-like phenotype by activating Wnt/β-catenin signalling. *Oncotarget*. (2017) 8(13):21461-71. doi: 10.18632/oncotarget.15591.
336. Lei S, He Z, Chen T, Guo X, Zeng Z, Shen Y, et al. Long noncoding RNA 00976 promotes pancreatic cancer progression through OTUD7B by sponging miR-137 involving EGFR/MAPK pathway. *J Exp Clin Cancer Res*. (2019) 38(1):470. doi: 10.1186/s13046-019-1388-4.
337. Zhou B, Guo W, Sun C, Zhang B, Zheng F. Linc00462 promotes pancreatic cancer invasiveness through the miR-665/TGFBR1-TGFBR2/SMAD2/3 pathway. *Cell Death Dis*. (2018) 9(6):706. doi: 10.1038/s41419-018-0724-5.
338. Chen X, Wang J, Xie F, Mou T, Zhong P, Hua H, et al. Long noncoding RNA LINC01559 promotes pancreatic cancer progression by acting as a competing endogenous RNA of miR-1343-3p to upregulate RAF1 expression. *Aging*. (2020) 12(14):14452-66. doi: 10.18632/aging.103487.
339. Yuan ZJ, Yu C, Hu XF, He Y, Chen P, Ouyang SX. LINC00152 promotes pancreatic cancer cell proliferation, migration and invasion via targeting miR-150. *Am J Transl Res*. (2020) 12(5):2241-56.
340. Chen S, Chen JZ, Zhang JQ, Chen HX, Qiu FN, Yan ML, et al. Silencing of long noncoding RNA LINC00958 prevents tumor initiation of pancreatic cancer by acting as a sponge of microRNA-330-5p to down-regulate PAX8. *Cancer Lett*. (2019) 446:49-61. doi: 10.1016/j.canlet.2018.12.017.
341. Arinjita B, Tanujit C, Shesh NR. Stochastic forecasting of COVID-19 daily new cases across countries with a novel hybrid time series model. *medRxiv*. (2021):2020.10.01.20205021. doi: 10.1101/2020.10.01.20205021.
342. Zhu X, Niu X, Ge C. Inhibition of LINC00994 represses malignant behaviors of pancreatic cancer cells: interacting with miR-765-3p/RUNX2 axis. *Cancer Biol Ther*. (2019) 20(6):799-811. doi: 10.1080/15384047.2018.1564566.

343. Liu C, Wang JO, Zhou WY, Chang XY, Zhang MM, Zhang Y, et al. Long non-coding RNA LINC01207 silencing suppresses AGR2 expression to facilitate autophagy and apoptosis of pancreatic cancer cells by sponging miR-143-5p. *Mol Cell Endocrinol*. (2019) 493:110424. doi: 10.1016/j.mce.2019.04.004.
344. Tong H, Liu X, Li T, Qiu W, Peng C, Shen B, et al. MACC1-AS1 promotes hepatocellular carcinoma cell invasion and proliferation by regulating PAX8. *Aging*. (2020) 12(1):70-9. doi: 10.18632/aging.102585.
345. Wu L, Liu Y, Guo C, Shao Y. LncRNA OIP5-AS1 promotes the malignancy of pancreatic ductal adenocarcinoma via regulating miR-429/FOXD1/ERK pathway. *Cancer Cell Int*. (2020) 20:296. doi: 10.1186/s12935-020-01366-w.
346. Sun J, Zhang P, Yin T, Zhang F, Wang W. Upregulation of LncRNA PVT1 Facilitates Pancreatic Ductal Adenocarcinoma Cell Progression and Glycolysis by Regulating MiR-519d-3p and HIF-1A. *J Cancer*. (2020) 11(9):2572-9. doi: 10.7150/jca.37959.
347. Yin Z, Zhou Y, Ma T, Chen S, Shi N, Zou Y, et al. Down-regulated lncRNA SBF2-AS1 in M2 macrophage-derived exosomes elevates miR-122-5p to restrict XIAP, thereby limiting pancreatic cancer development. *J Cell Mol Med*. (2020) 24(9):5028-38. doi: 10.1111/jcmm.15125.
348. Jian Y, Fan Q. Long non-coding RNA SNHG7 facilitates pancreatic cancer progression by regulating the miR-146b-5p/Robo1 axis. *Exp Ther Med*. (2021) 21(4):398. doi: 10.3892/etm.2021.9829.
349. Cao W, Zhou G. LncRNA SNHG12 contributes proliferation, invasion and epithelial-mesenchymal transition of pancreatic cancer cells by absorbing miRNA-320b. *Biosci Rep*. (2020) 40(6). doi: 10.1042/bsr20200805.
350. Deng PC, Chen WB, Cai HH, An Y, Wu XQ, Chen XM, et al. LncRNA SNHG14 potentiates pancreatic cancer progression via modulation of annexin A2 expression by acting as a competing endogenous RNA for miR-613. *J Cell Mol Med*. (2019) 23(11):7222-32. doi: 10.1111/jcmm.14467.
351. Al-Kafaji G, Al-Mahroos G, Abdulla Al-Muhtaresh H, Sabry MA, Abdul Razzak R, Salem AH. Circulating endothelium-enriched microRNA-126 as a potential biomarker for coronary artery disease in type 2 diabetes mellitus patients. *Biomarkers*. (2017) 22(3-4):268-78. doi: 10.1080/1354750x.2016.1204004.
352. Guo W, Zhong K, Wei H, Nie C, Yuan Z. Long non-coding RNA SPRY4-IT1 promotes cell proliferation and invasion by regulation of Cdc20 in pancreatic cancer cells. *PLoS One*. (2018) 13(2):e0193483. doi: 10.1371/journal.pone.0193483.
353. Cui XP, Wang CX, Wang ZY, Li J, Tan YW, Gu ST, et al. LncRNA TP73-AS1 sponges miR-141-3p to promote the migration and invasion of pancreatic cancer cells through the up-regulation of BDH2. *Biosci Rep*. (2019) 39(3). doi: 10.1042/bsr20181937.
354. Duan Q, Xu M, Wu M, Zhang X, Gan M, Jiang H. Long noncoding RNA UCA1 promotes cell growth, migration, and invasion by targeting miR-143-3p in oral squamous cell carcinoma. *Cancer Med*. (2020) 9(9):3115-29. doi: 10.1002/cam4.2808.
355. Wei W, Liu Y, Lu Y, Yang B, Tang L. LncRNA XIST Promotes Pancreatic Cancer Proliferation Through miR-133a/EGFR. *J Cell Biochem*. (2017) 118(10):3349-58. doi: 10.1002/jcb.25988.
356. Gao H, Gong N, Ma Z, Miao X, Chen J, Cao Y, et al. LncRNA ZEB2-AS1 promotes pancreatic cancer cell growth and invasion through regulating the miR-204/HMGB1 axis. *Int J Biol Macromol*. (2018) 116:545-51. doi: 10.1016/j.ijbiomac.2018.05.044.

357. Sun YW, Chen YF, Li J, Huo YM, Liu DJ, Hua R, et al. A novel long non-coding RNA ENST00000480739 suppresses tumour cell invasion by regulating OS-9 and HIF-1α in pancreatic ductal adenocarcinoma. *Br J Cancer*. (2014) 111(11):2131-41. doi: 10.1038/bjc.2014.520.
358. Pan S, Shen M, Zhou M, Shi X, He R, Yin T, et al. Long noncoding RNA LINC01111 suppresses pancreatic cancer aggressiveness by regulating DUSP1 expression via microRNA-3924. *Cell Death Dis*. (2019) 10(12):883. doi: 10.1038/s41419-019-2123-y.
359. Li K, Han H, Gu W, Cao C, Zheng P. Long non-coding RNA LINC01963 inhibits progression of pancreatic carcinoma by targeting miR-641/TMEFF2. *Biomed Pharmacother*. (2020) 129:110346. doi: 10.1016/j.biopha.2020.110346.
360. Li X, Zhou S, Fan T, Feng X. lncRNA DGCR 5/miR-27a-3p/BNIP3 promotes cell apoptosis in pancreatic cancer by regulating the p38 MAPK pathway. *Int J Mol Med*. (2020) 46(2):729-39. doi: 10.3892/ijmm.2020.4632.
361. Gu L, Zhang J, Shi M, Zhan Q, Shen B, Peng C. lncRNA MEG3 had anti-cancer effects to suppress pancreatic cancer activity. *Biomed Pharmacother*. (2017) 89:1269-76. doi: 10.1016/j.biopha.2017.02.041.
362. Gao ZQ, Wang JF, Chen DH, Ma XS, Wu Y, Tang Z, et al. Long non-coding RNA GAS5 suppresses pancreatic cancer metastasis through modulating miR-32-5p/PTEN axis. *Cell Biosci*. (2017) 7:66. doi: 10.1186/s13578-017-0192-0.
363. Wang X, Gao X, Tian J, Zhang R, Qiao Y, Hua X, et al. LINC00261 inhibits progression of pancreatic cancer by down-regulating miR-23a-3p. *Arch Biochem Biophys*. (2020) 689:108469. doi: 10.1016/j.abb.2020.108469.
